# Reliability of task‐evoked neural activation during face‐emotion paradigms: Effects of scanner and psychological processes

**DOI:** 10.1002/hbm.25723

**Published:** 2022-02-15

**Authors:** Simone P. Haller, Gang Chen, Elizabeth R. Kitt, Ashley R. Smith, Joel Stoddard, Rany Abend, Sofia I. Cardenas, Olga Revzina, Daniel Coppersmith, Ellen Leibenluft, Melissa A. Brotman, Daniel S. Pine, David Pagliaccio

**Affiliations:** ^1^ Emotion and Development Branch National Institute of Mental Health, National Institutes of Health Bethesda Maryland USA; ^2^ Scientific and Statistical Computing Core National Institute of Mental Health, National Institutes of Health Bethesda Maryland USA; ^3^ Pediatric Mental Health Institute, Children's Hospital Colorado, Department of Psychiatry & Neuroscience Program University of Colorado, Anschutz Medical Campus Aurora Colorado USA; ^4^ Division of Child and Adolescent Psychiatry, Department of Psychiatry Vagelos College of Physicians and Surgeons New York State Psychiatric Institute, Columbia University New York New York USA

**Keywords:** face‐emotion, fMRI, intraclass correlation coefficient, reliability, test–retest

## Abstract

Assessing and improving test–retest reliability is critical to efforts to address concerns about replicability of task‐based functional magnetic resonance imaging. The current study uses two statistical approaches to examine how scanner and task‐related factors influence reliability of neural response to face‐emotion viewing. Forty healthy adult participants completed two face‐emotion paradigms at up to three scanning sessions across two scanners of the same build over approximately 2 months. We examined reliability across the main task contrasts using Bayesian linear mixed‐effects models performed voxel‐wise across the brain. We also used a novel Bayesian hierarchical model across a predefined whole‐brain parcellation scheme and subcortical anatomical regions. Scanner differences accounted for minimal variance in temporal signal‐to‐noise ratio and task contrast maps. Regions activated during task at the group level showed higher reliability relative to regions not activated significantly at the group level. Greater reliability was found for contrasts involving conditions with clearly distinct visual stimuli and associated cognitive demands (e.g., face vs. nonface discrimination) compared to conditions with more similar demands (e.g., angry vs. happy face discrimination). Voxel‐wise reliability estimates tended to be higher than those based on predefined anatomical regions. This work informs attempts to improve reliability in the context of task activation patterns and specific task contrasts. Our study provides a new method to estimate reliability across a large number of regions of interest and can inform researchers' selection of task conditions and analytic contrasts.

## Graphical abstract

The current study uses two statistical approaches to examine how scanner and task‐related factors influence reliability of fMRI response to face‐emotion viewing. Forty healthy adult participants completed two face‐emotion paradigms at up to three scanning sessions across two scanners of the same build over approximately 2 months. We examined reliability across the main task contrasts using Bayesian linear mixed‐effects models performed voxel‐wise across the brain. We also used a novel Bayesian hierarchical model across a predefined whole‐brain parcellation scheme and subcortical anatomical regions.

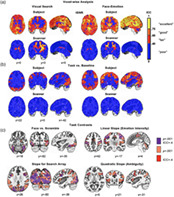

## 1 INTRODUCTION

Concerns about replicability (Open Science Collaboration, 2015) in functional magnetic resonance imaging (fMRI) work are growing (e.g., Poldrack et al., 2017). Improving test–retest reliability is a cornerstone of addressing these concerns. A recent meta‐analysis (Elliott et al., 2019) suggests that test–retest reliability of fMRI task contrasts is often relatively poor (e.g., intra‐class correlation coefficients [ICCs] < .4). The current study uses two statistical approaches to examine how scanner effects and task‐related factors influence reliability. The study focuses specifically on task‐evoked activation during two face‐emotion‐viewing paradigms.

Across experimental paradigms, several factors are known to influence fMRI reliability. These include scanner‐ or site‐related factors, participant‐related factors, and time‐related change. Several studies have shown that only small proportions of variance tend to be affected by scanner differences (e.g., Gountouna et al., 2010; Gradin et al., 2010; Yendiki et al., 2010). However, as such studies can often confound scanner and practice effects, we use a pseudo‐random assignment to two scanners across three time points, thereby separating scanner‐ and time‐related variance.

Face‐emotion paradigms are often used in studies of individual differences as affect‐evoking stimuli. In prior studies, the reliability of fMRI face‐emotion paradigms varied by task condition. For example, prior work typically finds moderate reliability for face vs. baseline contrasts, but poor reliability for contrasts between specific face‐emotion types, for example, angry vs. neutral (Haller et al., 2018; Plichta et al., 2012; Sauder, Hajcak, Angstadt, & Phan, 2013; van den Bulk et al., 2013; White et al., 2016). The current study utilizes two tasks that differ in their cognitive demands. One task involves implicit face‐emotion processing, such that face‐emotion monitoring is irrelevant to task performance; the other involves explicit face‐emotion judgments.

Many earlier reliability studies focused on a priori regions‐of‐interest (ROIs), whereas newer statistical methods have become available for whole‐brain reliability analyses. That said, common approaches to multiple comparisons correction for whole‐brain analyses, for example, cluster‐correction, rely on profound data reduction that may reduce reliability (Chen et al., 2019; Woo, Krishnan, & Wager, 2014). Significance tests are conducted independently per voxel; this massive multiplicity is accounted for by estimating the probability of a number of contiguous voxels all exhibiting significant effects. A complementary approach is to leverage the substantial information present in fMRI scans by using rational, Bayesian principles that mitigate data reduction by accounting for uncertainty (Chen, Taylor, Cox, & Pessoa, 2020). Therefore, this study includes a recent translation of Bayesian methods for group‐level fMRI analysis, measuring reliability through two approaches. First, we examined a conventional, voxel‐wise linear mixed‐effects model with cluster‐based correction. Second, we used a hierarchical Bayesian approach that examines ROIs across the whole brain, defined independently of the study data. For this second approach, results are reported based on an open‐source, publicly available Bayesian hierarchical model developed for fMRI (Chen et al., 2019). This method enables test–retest analyses that incorporate all ROIs into one model to mitigate the issue of multiple testing over many units.

The current study examines 40 healthy adult participants using two face‐emotion paradigms, one requiring explicit face‐emotion labeling and one involving implicit, task‐irrelevant face‐emotion processing. Participants completed up to three scanning sessions over approximately 2 months. We examine reliability using Bayesian linear mixed‐effects models performed voxel‐wise across the brain and a novel Bayesian hierarchical model in predefined ROIs. Participants were pseudo‐randomized and scanned across two comparable 3T GE MRIs, as would be common in single‐site or harmonized multi‐site studies. We expect scanner to account for minimal variance in temporal signal‐to‐noise ratio (tSNR) and fMRI task contrast maps. Moreover, we expect higher reliability among regions activated during the task at the group level (i.e., regions showing significant task contrast activity at the first scan session) relative to regions not activated significantly at the group level. Finally, we expect to see greater reliability for contrasts involving conditions with clearly distinct visual stimuli and associated cognitive demands (e.g., face vs. nonface discrimination) compared to conditions with more similar demands (e.g., angry vs. happy discrimination).

## 2 METHODS

### 2.1 Participants

Forty‐five participants enrolled in an institutional review board‐approved protocol at the National Institute of Mental Health in Bethesda, MD. Participants provided written informed consent. All participants were >18 years old (Age: *M* = 31.95 years, *SD* = 9.39; 58% female). Participants were excluded for any current psychiatric conditions, as determined by the Structured Clinical Interview for *DSM‐IV* Disorders (Spitzer, Williams, Gibbon, & First, 1992). Further exclusionary criteria included IQ < 70 on the Wechsler Abbreviated Scale of Intelligence (WASI; Wechsler, 1999), alcohol or substance abuse within the last 3 months, significant medical illness, head trauma, neurological disorder, current psychotropic medication use, or contraindications to MRI.

Of the 45 individuals who enrolled in the study, 40 participants (Age: *M* = 31.76, *SD* = 9.43; 58% female; IQ: *M* = 114.70, *SD* = 11.16; 38% White, 33% Black or African American, 13% Asian, 16% other) provided useable behavioral and imaging data for at least two sessions for at least one of the two task paradigms. Participant data with excessive head motion or poor task performance were excluded (Section 2.5.3). Participants with only one useable scan after scan quality assessment were excluded. The final number of participants included in all analyses for the visual search task was *N*[Session 1–3] = 40, 35, 33 and for the emotion labeling task: *N*[Session 1–3] = 29, 33, 30 (Figure S1).

### 2.2 Task paradigms

Participants completed up to three MRI sessions, across two scanners in an ABA or BAB order, pseudo‐randomized across participants. Scanning sessions were 2–6 weeks apart (S1–S2: *M* = 22.39 days, *SD* = 8.49; S2–S3: *M* = 28.83 days, *SD* = 12.80) and participants were compensated for each session.

During each scan, participants completed two tasks, a visual search with emotional distractors, and an explicit labeling emotional face task. The order of task completion was counterbalanced across participants (but consistent within‐participant across session).

#### 2.2.1 Visual search task

**FIGURE 1:**
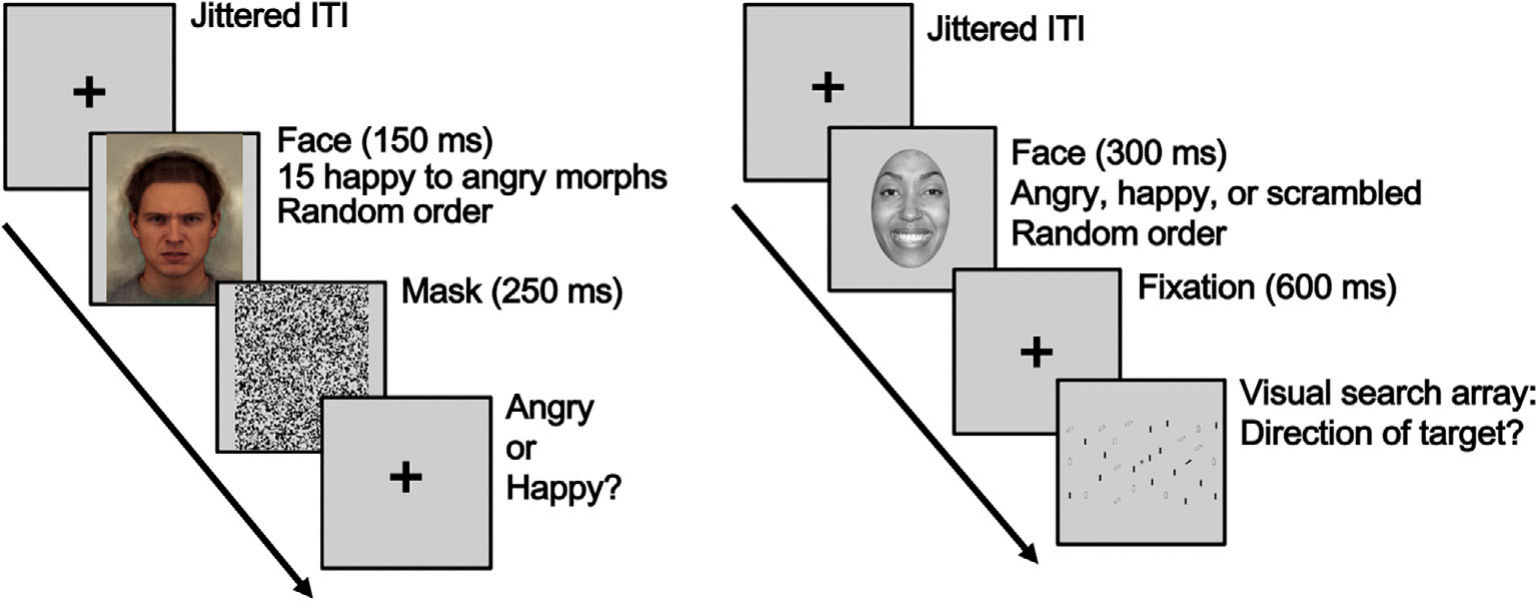
Schematics of in‐scanner tasks. *Left panel*: Visual search task. *Right panel*: Face‐emotion labeling task

This task, which was modified and adapted for fMRI from a previously used paradigm (Haas, Amso, & Fox, 2017), required participants to find a target stimulus in search array following an emotional face image. Each trial consisted of a grayscale face stimulus (angry, happy, or scrambled control) presented for 300 ms, then a 600 ms fixation cross, followed by a visual search array with one black bar target slanted left or right and 0, 4, or 29 distractors (slanted or vertical white bars and vertical black bars) displayed for a 2,000 ms response window (Figure 1). Participants were required to find the target bar and indicate the direction that it was slanted (left or right) via a response box button press. Emotional face stimuli were images from 16 actors displaying angry or happy expression drawn from an available stimulus set (Tottenham et al., 2009). Face stimuli were cropped to a face‐shaped oval and set to grayscale. The pixels of a face stimulus were scrambled to create a control stimulus matched on visual properties but without any face properties. A fixation cross was presented between trials for a jittered inter‐trial interval (ITI; min = 500 ms, ITI distribution followed an exponential decay curve). Stimulus order and ITI jitter were optimized using AFNI's make_random_timing.py program; 200 different optimizations were selected and randomized across scan sessions. Participants completed a total of 243 trials (27 instances of 9 trial types: 3 emotions [angry, happy, scrambled] × 3 search array sizes [1, 5, 30 bars]) across three runs. Each run was 365 s long, including ~10 s of fixation at the beginning and end of each run.

#### 2.2.2 Face‐emotion labeling task

This task was adapted for fMRI from a previously used behavioral paradigm (Stoddard et al., 2016). Participants were required to judge the emotion of a composite male face drawn from the Karolinska Directed Emotional Faces (Lundqvist & Litton, 1998). Stimuli were 15 face‐emotion expressions equally spaced/morphed on a continuum from prototypically angry to prototypically happy. On each trial, a face morph was presented for 150 ms followed by a 250 ms white noise mask, and then a response screen with a fixation cross for 2,000 ms (Figure 1). Participants had to indicate whether the briefly presented face displayed an angry or happy expression via a button box press. A fixation cross was presented for a jittered ITI between trials (min 500 ms, ITI distribution followed an exponential decay curve). Stimulus presentation and jitter orders were optimized and pseudo‐randomized using AFNI's make_random_timing.py program. Participants completed a total of 540 trials across four runs, including 90 fixation trials (i.e., each morph was presented 30 times). Each run was 412 s long with ~10 s of fixation at the beginning and end of each run.

### 2.3 Behavioral data

Accuracy and reaction time data were examined for each task, see details by task below.

#### 2.3.1 Visual search task

Accuracy and mean reaction time (to identify the slant of the target bar) were calculated as a function of condition: face‐emotion (angry, happy, scrambled control) and search array size (1, 5, and 30 bars). Sessions with accuracy <70% and/or >15% nonresponses were excluded (2 sessions for 1 participant). The effect of emotion, search array size, and their interaction were of interest here.

#### 2.3.2 Face‐emotion labeling task

As in prior work (Stoddard et al., 2016), a four‐parameter logistic curve was fit to each participant's choice‐response data (parameters included: upper limit, lower limit, slope, and inflection point of the logistic curve, that is, the morph/emotional intensity where judgments switch from predominantly happy to angry, adjusted for the maximum probability of either judgment). An inflection point of 8 indicates no bias (middle of morphs 1–15), whereas a lower inflection point indicates a hostile interpretation bias, that is, a tendency to judge ambiguous faces as angry, rather than happy. We examined both inflection point and slope from the logistic regressor for the behavioral data. Reaction time was examined as linear slope (coding emotion intensity from angry to happy) and quadratic slope (coding ambiguity from ambiguous to overt) across face morphs. Additional reaction time indices (i.e., reaction time difference scores) are presented in the Supporting Information. Data from participants who failed to correctly identify at least 70% of the emotional expression of the overtly angry and happy facial expressions or had more than 15% of missed responses were excluded (8 sessions across 6 participants).

### 2.4 Test–retest assessment

#### 2.4.1 Behavioral data

Test–retest reliability of task behavior across three scanning sessions was tested in a Bayesian framework using linear mixed‐effects models in R v3.5.0 (R Core Team, 2015) using the *blme* package (Chung, Rabe‐Hesketh, Dorie, Gelman, & Liu, 2013). This included a random effect for participant modeled with Gamma priors (shape = 2, rate = 0.5) and three fixed effects, one for scanner, one for visit and one for the order of task acquisition in the scanner. Intraclass correlation coefficients were estimated as the proportion of participant‐specific variance out of total variance (Bartko, 1966; Shrout & Fleiss, 1979). This approach mirrored that used at the voxel‐level in the fMRI analyses described below. ICCs were calculated for task contrasts of interest (see below).

#### 2.4.2 Visual search task

ICCs were calculated for two reaction time contrasts: faces vs. scramble control difference scores and log‐transformed slope across search array size for each emotion and scramble control stimuli. Supporting Information additionally contains ICCs for array size 30 vs. 1 and happy versus angry differences scores and log‐transformed slope across all emotions, as well as the ICC for the difference in accuracy for search array size 30 vs. 1.

#### 2.4.3 Face‐emotion labeling task

ICCs were calculated for inflection point and slope of the choice response data as well as for linear and quadratic slopes of reaction time data across face emotion morphs. Refer to Supporting Information for additional contrasts (e.g., ambiguous vs. overt and happy vs. angry faces).

### 2.5 Imaging data

#### 2.5.1 Acquisition

Neuroimaging data were collected on two 3T General Electric Signa 750 scanners each using a 32‐channel head coil with identical acquisition sequences. After a sagittal localizer scan, an automated shim calibrated the magnetic field to reduce signal dropout due to susceptibility artifact. BOLD signal was measured by T2*‐weighted echo‐planar imaging at a voxel resolution of 2.5 × 2.5 × 3.0 mm (repetition time = 2,300 ms, field of view = 24.0 mm, frequency × phase: 96 × 96; face‐emotion labeling: 179 volumes, flip angle = 70°, echo time = 30 ms, visual search: 151 volumes, flip angle = 75°, echo time = 25 ms). To reach longitudinal magnetization equilibrium, the four initial images from each run were discarded. A structural MPRAGE scan (echo time = min full; inversion time = 425; field of view = 25.6; frequency × phase = 256 × 256; flip angle = 7°; 1 mm isotropic voxels) was acquired for co‐registration with the functional data.

#### 2.5.2 Imaging preprocessing

Neuroimaging data were analyzed using Analysis of Functional NeuroImages (AFNI; http://afni.nimh.nih.gov/afni/; Cox, 1996) v18.3.03 with standard preprocessing, including despiking, slice‐timing correction, distortion correction, alignment of all volumes to a base volume (MIN_OUTLIER), nonlinear registration to the MNI template, spatial smoothing to 6.5 mm FWHM kernel (using blur_to_fwhm flag), masking, and intensity scaling. Spatial smoothing to a desired blur size assures that a similar smoothness is achieved across scanners and sessions, rather than adding a set blur kernel to acquired images that may vary in initial smoothness. First‐level models were created with generalized least squares time series fit with restricted maximum likelihood estimation of the temporal autocorrelation structure (3dREMLfit). This work utilized the computational resources of the NIH HPC Biowulf cluster (http://hpc.nih.gov).

This processing and first‐level general linear models (GLM) controlled for head motion. Specifically, we regressed any pair of successive TRs where the sum head displacement (Euclidean norm of the derivative of the translation and rotation parameters) between those TRs exceeded 0.5 mm. TRs, where more than 10% of voxels were timeseries signal outliers, were also excluded. Sessions were excluded if the average motion per TR after censoring was >0.25 mm or if >15% of TRs were censored for motion/outliers. Additionally, six head motion parameters were included as nuisance regressors in individual‐level models. Temporal signal‐to‐noise ratio (tSNR = average signal/standard deviation of noise [GLM residuals]) maps were created from the first‐level model output.

##### Visual search task

Regressors for nine trial types of interest (3 emotion by 3 search array size) and error trials were included in first‐level GLMs. These were modeled with a block hemodynamic response function (BLOCK (2.9,1)). Four first‐level contrasts were created for each participant to examine: task vs. fixation, faces vs. scrambled control, search array 30 vs. 1, and a log‐linear slope across search array size. Figure S2 displays additional contrasts of angry versus happy, a log slope per emotion, and 30 versus 1 search array.

##### Face‐emotion labeling task

Fifteen regressors of interest were included to represent the 15 face emotion morphs, modeled with a block hemodynamic response function (BLOCK (0.15,1)). Separately, two amplitude‐modulated regressors as a function of face morph weighed in a linear and quadratic fashion (AM2), as well as error trials modeled without amplitude modulation, were coded. Three first‐level contrasts were created for each participant: task (15 face‐emotion regressors) vs. fixation, amplitude‐modulated linear slope across morphs (coding emotion intensity from angry to happy), amplitude modulated quadratic slope across morphs (coding ambiguity from ambiguous to overt). Additional contrasts of subtraction values: ambiguous vs. overt faces and angry vs. happy faces are presented in the Supporting Information.

#### 2.5.3 Imaging analysis

##### Activation at Session 1

Linear mixed‐effects models (3dLME; Chen et al., 2013) with participant as a random effect were computed for the first scan session to examine group average activity for each task condition. Models included scanner and task order (to indicate which behavioral task was performed first in the scanner) as fixed‐effects covariates. Monte Carlo simulations were performed using AFNI's 3dClustSim to correct for multiple comparisons. All analyses were restricted to a whole‐brain mask of 98,386 voxels where 90% of participants (completing either/both tasks) had useable data at Session 1. Smoothness of the residuals was estimated based on a Gaussian plus mono‐exponential spatial autocorrelation function (3dFWHMx with ‐acf flag) for all participants and averaged yielding an effective smoothness of FWHM = 9.14 mm (ACF parameters, a = 0.61, b = 3.37, c = 10.88). Two‐sided thresholding was examined for whole‐brain tests with first‐nearest neighbor clustering (NN = 1). To obtain a whole‐brain family‐wise error correction of *p* < .05, all results were thresholded at a voxel‐wise *p* < .001 and a cluster extent of *k* = 20 voxels.

##### Voxel‐wise test–retest assessment

Bayesian linear mixed‐effects models (3dLME; Chen et al., 2018) were used to compute voxel‐wise ICC of BOLD activation across the three MRI sessions. The Bayesian ICC approach has been demonstrated to address potential issues in traditional ICC estimates (e.g., negative ICC values, missing data, confounding effects). Linear mixed‐effects models included a fixed effect for task order and visit and random effects with Gamma priors (Chen, Saad, Britton, Pine, & Cox, 2013) for participant and scanner, to estimate of the proportion of participant, scanner, and residual error variance per voxel for tSNR and task versus baseline (ICCs with absolute agreement (ICC[2,1]; Shrout & Fleiss, 1979). For task contrasts, we used ICCs with consistency formulation (ICC[3,1]; examining the consistency in rank rather than absolute value, which accounts for systematic changes over time, such as practice effects) with participant as a random effect and fixed effects for scanner, task order, and visit. For display purposes, ICC maps of participant‐specific variance were binned into color schemes representing “poor” (ICC < 0.4), “fair” (ICC = .4–.6), “good” (ICC = .6–.75), and “excellent” (ICC > .75) test–retest reliability.

Conjunction maps (Figure S2) were created for display purposes to illustrate the overlap in brain regions that were robustly activated by the task (at the first scanning session; cluster‐corrected) and reliably activated across scanner and time (ICC > 0.4). To statistically test whether more active regions are also more reliable, we use AFNI's 3ddot function to examine whole‐brain voxel‐wise correlations between first scanning session tSNR or task activation and their associated ICC maps. Additionally, we examined associations between mean tSNR at the first scanning session and the task versus baseline ICC maps to assess how tSNR may influence task reliability. AFNI's 3ddot provides a single correlation coefficient describing the association between two voxel‐wise maps.

##### ROI‐based test–retest assessment

As an alternative to voxel‐wise testing with cluster‐based multiple comparisons correction, we conducted ICC analyses across 214 ROIs covering the whole brain (defined independently of our reliability estimates). These included 200 parcels from a published cortical parcellation (Schaefer et al., 2018) and 14 subcortical ROIs from the Harvard–Oxford probabilistic atlas (75% probability for defining the hippocampus; 50% probability for defining other regions). Contrast activity was extracted from all ROIs across the three scanning sessions for both tasks and used in these analyses. ICCs at the ROI level was inferred through a Bayesian multilevel model that integrated all regions (Chen et al., 2019, 2020). Specifically, each effect was decomposed into three components that are associated with the variability across subjects, visits, and regions while the scanner and task effects were modeled as covariates with the following Bayesian multi‐level formulation,

$$y_{\mathrm{ijk}}=a_{0}+a_{1}+a_{2}+\xi_{0i}+\xi_{1i}+\xi_{2i}+\eta_{j}+\zeta_{0k}+\zeta_{1k}+\zeta_{2k}+\gamma_{\mathrm{ij}}+\mu_{0\mathrm{ik}}+\mu_{1\mathrm{ik}}+\mu_{2\mathrm{ik}}+\nu_{0\mathrm{jk}}+\nu_{1\mathrm{jk}}+\nu_{2\mathrm{jk}}+\varepsilon_{\mathrm{ijk}},$$

where $a_{0}$, $a_{1}$, and $a_{2}$ code the intercept, scanner, and task effects, respectively; $\;\xi_{0i}$, $\;\xi_{1i}$, and $\;\xi_{2i}$ represent the intercept, scanner, and task effects during the *i*th session (visit); $\eta_{j}$ models the effect of *j*th subject; $\gamma_{\mathrm{ij}}$ characterizes the effect of *j*th subject during *i*th session; $\zeta_{0k}$, $\zeta_{0k}$, and $\zeta_{0k}$ are the intercept, scanner, and task effects at the *k*th ROI; the μ and μ terms are the intercept, scanner, and task effects of the *i*th session at the *k*th ROI and the *j*th subject at the *k*th ROI, respectively; finally, ε is the residual term. With a Gaussian assumption for cross‐session, cross‐participant, cross‐ROI effects, their interactions, and residuals, the Bayesian model is numerically solved through Markov Chain Monte Carlo simulations using the R package *brms* (Bürkner, 2017) in Stan (Carpenter, 2017). The ICC at the *k*th ROI was assessed through the mean, standard error, and quantile interval based on the variances ($\sigma^{2}$) of the corresponding posterior density:

$${\mathrm{ICC}}_{k}=\frac{\sigma_{\eta }^{2}+\sigma_{\zeta_{0k}}^{2}+\sigma_{\nu_{0k}}^{2}}{\sigma_{\eta }^{2}+\sigma_{\zeta_{0k}}^{2}+\sigma_{\nu_{0k}}^{2}+\sigma_{\xi_{0}}^{2}+\sigma_{\gamma }^{2}+\sigma_{\mu_{0k}}^{2}+\sigma_{\nu_{0k}}^{2}+\sigma_{\varepsilon }^{2}}\;\left(k=1,2,\ldots ,214\right)$$

The integrative Bayesian model has two main advantages for ICC computation over the conventional approach through linear mixed‐effects modeling (Chen et al., 2018). One is to dissolve multiple testing by incorporating all spatial units (regions) into one model and relying on “global calibration” across units; in contrast, conventional voxel‐wise ICC analysis typically requires cluster‐based correction for multiple comparisons. The other advantage is the availability of uncertainty estimates and standard deviations based on the posterior sample draws, compared to the difficulty of assigning adequate degrees of freedom in a linear mixed‐effects model. For additional details on the Bayesian multilevel formulation and tables comparing the Bayesian model to the conventional linear mixed‐effects ICCs for each of the 200 cortical parcels and 14 subcortical ROIs, see Supporting Information.

## 3 RESULTS

### 3.1 Behavioral data

#### 3.1.1 Visual search task

At the first scan session, a significant effect of search array size (*F*[1.78, 69.24] = 13.94, *p* < .001) indicated lower accuracy in the 30 search array condition compared to both the 0 (*t*[78] = 5.16, *p* < .001) and 5 search array condition (*t*[78] = 3.55, *p* = .002). For reaction time, both an effect of search array (*F*[1.72, 67.16] = 270.33, *p* < .001) and emotion emerged (*F*[1.81, 70.70] = 3.28, *p* = .048), with slower reaction times with increasing distractors (0 vs. 4: *t*(78) = −8.89, *p* < .001, 4 vs. 29: *t*(78) = −14.16, *p* < .001, 0 vs. 29: *t*(78) = −23.05, *p* < .001) and following angry faces compared to scramble control stimuli (*t*(78) = −2.53, *p* < .04). No search array by emotion interaction was noted (all *p*s > .05). Test–retest reliability for reaction time was poor for the faces vs. scramble control difference score (ICC = .12), but moderate–good for the log‐transformed slope by search array (angry: ICC = .57, happy: ICC = .64, scramble control: ICC = .68, average log‐transformed slope across emotions: ICC = .83). Refer to Table S1 for a summary of ICCs for behavioral metrics.

#### 3.1.2 Face‐emotion labeling task

A significant quadratic trend emerged for reaction time (*F*[1, 404] = 147.04, *p* < .001) with markedly slower responses to ambiguous relative to overt happy or angry faces (*t*[404] = −12.13, *p* < .001). No significant effect was noted for the linear trend (angry to happy) on reaction time (p > .05). ICCs for the contrasts of interest were ICC = .55 (inflection point of the logistic regressor), ICC = .46 (slope of the logistic regressor), ICC = .55 (linear slope for reaction time), and ICC = .59 (quadratic slope for reaction time). Refer to Table S2 for a summary of ICCs for behavioral metrics.

### 3.2 Imaging data

#### 3.2.1 Scanner effects on tSNR

We first investigated scanner effects on tSNR using both voxel‐wise analyses and ROIs covering the whole brain.

##### Voxel‐wise analysis

**FIGURE 2:**
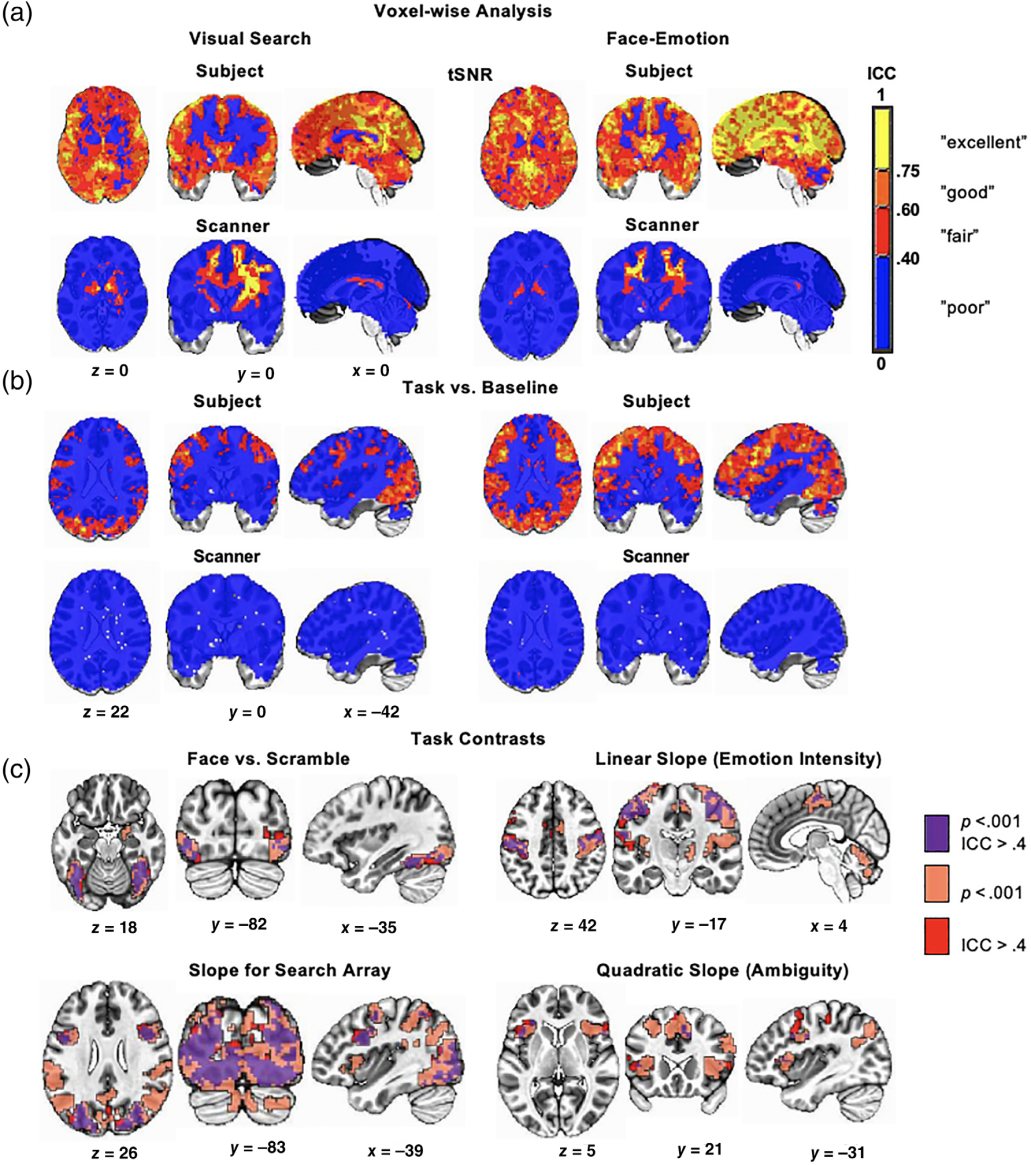
Voxel‐wise analysis. (a) Scanner effects on temporal signal‐to‐noise ratio (tSNR) and (b) task versus baseline contrasts. For both tasks, participant‐specific variance in tSNR was highly reliable over time. Higher ICCs for scanner‐specific relative to participant‐specific effects were only seen in white matter. (c) Conjunction maps between the first group‐level activation for main task contrast at a corrected significance level of .05 based on voxel‐wise *p* < .001 and ICC maps at a threshold of ICC > 0.4

Average tSNR (at the first scan) for the visual search task was *M* = 212.84 (*SD* = 28.58) and for the face‐emotion labeling task *M* = 223.33 (*SD* = 28.01). For both tasks, we found participant‐specific variance in tSNR to be highly reliable across the three scanning sessions (Figure 2a). Higher ICCs for scanner‐specific relative to participant‐specific effects were only seen in white matter. The mean tSNR map (at the first session) was highly correlated with voxel‐wise participant‐specific ICCs for both paradigms (visual search: *r* = .92; face‐emotion labeling: *r* = .89), that is, voxels with higher tSNR were more reliable over scans.

##### ROI‐based analysis

**FIGURE 3:**
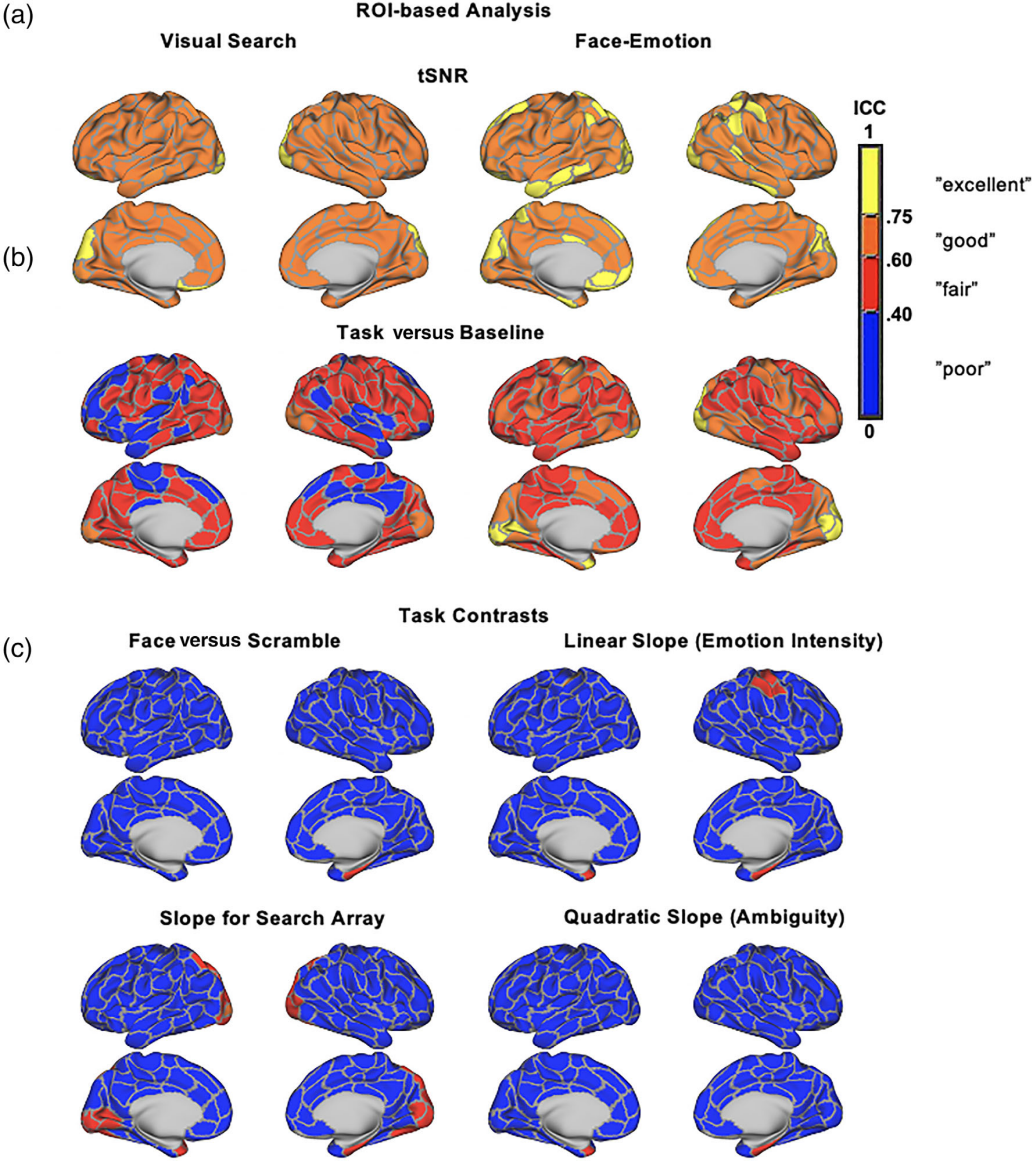
Surface renderings of unthresholded maps of ROI ICCs for both tasks using a Bayesian hierarchical model. (a) tSNR and (b) task versus baseline contrasts showed reliable participant‐specific variance, while reliability of main task contrasts faces versus scrambled contrast signal and log‐transformed slope exhibited patterns of largely “poor” reliability

Figure 3a displays a surface rendering of participant‐specific ICCs of cortical parcels for tSNR in the Bayesian multi‐level ROI analyses. Refer to Tables S11 and S13 for a list of participant‐specific ICCs for each of the 200 cortical parcels and 14 subcortical ROIs for each task, presented alongside the conventional linear mixed‐effects approach for each parcel.

#### 3.2.2 Reliability of task contrasts

We next investigated reliability of the task contrasts for each paradigm utilizing both voxel‐wise and ROI analyses. Table 1 contains “at‐a‐glance” summaries of reliability estimates of the main behavioral indices and fMRI contrasts for each task; detailed tables can be found in Tables S3–S6.

**TABLE 1:** Summary of reliability estimates for main behavioral indices and fMRI task contrasts

Face‐emotion labeling task		
*Behavioral indices*	ICC (3,1)	
Choice response: Inflection point	0.55	
Choice response: Slope	0.46	
Mean RT: Linear slope	0.55	
Mean RT: Quadratic slope	0.59	
*fMRI contrasts*	ICC (3,1) > .4	Activation *p* < .001
Linear slope	R & L postcentral gyrus, R & L precentral gyrus, R & L SMA, R & L cerebellum, R supramarginal gyrus, L paracentral lobule, L middle frontal gyrus, R superior parietal lobule	R & L postcentral gyrus, R & L cerebellum, R & L SMA, R & L Rolandic operculum, R & L thalamus, L putamen
Quadratic scope	R superior medial gyrus, R & L inferior frontal gyrus, L & R precentral gyrus, L SMA, R superior parietal lobule, L postcentral gyrus, L insula lobe	L SMA, R inferior frontal gyrus, R & L angular gyrus, R & L insula lobe, L middle frontal gyrus, L precentral gyrus, L mid orbital gyrus, L middle cingulate cortex, L superior frontal gyrus, L precuneus, R inferior occipital gyrus, R middle occipital gyrus

Visual search task		
*Behavioral indices*	ICC (3,1)	
Accuracy: 30–1	0.47	
Mean RT face versus scramble	0.12	
Mean RT log‐transformed slope	0.83	
*fMRI contrasts*	ICC (3,1) > .4	Activation *p* < .001
Face versus scramble	L middle occipital gyrus, R & L precentral gyrus, R inferior frontal gyrus, R inferior temporal gyrus, L middle temporal gyrus, L cuneus	R & L fusiform gyrus, R inferior occipital gyrus, R middle temporal gyrus, R hippocampus
Log‐transformed slope	R & L fusiform gyrus and R superior occipital gyrus	L middle occipital gyrus, L precentral gyrus, L precuneus, R & L superior frontal gyrus, R inferior frontal gyrus, L SMA, R thalamus, R calcarine gyrus, R supramarginal gyrus, R & L insula lobe, R & L parahippocampal gyrus, R cerebellum, R putamen, L superior temporal gyrus, R postcentral gyrus, cerebellar vermis

*Note*: This table provides an “at‐a‐glance” summary of behavioral and neural reliability findings alongside group‐level voxel‐wise activation patterns at the first scan session for the visual search and emotion labeling tasks. The test–retest reliability of main behavioral indices is noted, that is, the intra‐class correlation coefficient (ICC) of participant‐specific variance. Full behavioral reliability results are presented in Tables S1 and S2. A brief descriptive summary of regions exhibiting at least “fair” reliability (ICC > .4) in voxel‐wise analyses for main tasks contrasts is also presented, full results are presented in Tables S3–S6.

##### Voxel‐wise analysis of visual search task

First, for the task vs. baseline contrast, scanner‐associated variance was minimal, with no scanner‐associated variance surpassing the threshold of ICC > .4 (Figure 2b). Reliable participant‐specific variance was observed in visual, parietal, and prefrontal cortices, including the inferior‐frontal and middle frontal gyri. ICCs for the task vs. baseline contrast correlated positively with both mean tSNR (*r* = .82) and task vs. baseline activity (*r* = .60) at the voxel‐wise level.

Next, two task contrasts of interest were examined (Figure 2c). Faces vs. scrambled contrast signal in visual cortex/fusiform gyrus was both active at the first session and reliable at ICC > .4. Note that the right amygdala was also active at the first session but was not reliable at a threshold of ICC > .4. Average faces vs. scrambled contrast activity at the first session was weakly correlated with the associated reliability map (*r* = .29) at the voxel‐wise level.

The log‐transformed slope analysis revealed contrast signal in visual, parietal, and bilateral dorsal lateral prefrontal cortex (dlPFC) regions, signal that was also reliable across time. The anterior insular showed significant activation at the first session, but was not reliable at ICC > .4. Average log‐transformed slope activity at the first session was correlated with the associated reliability map (*r* = .55). Additional contrasts (angry vs. happy and log slopes per emotion) and tables detailing group‐level activation at the first scanning session and clusters of ICC > .4 are presented in Figure S2.

##### Voxel‐wise analysis of face‐emotion labeling task

As above, scanner‐associated variance was minimal for the task vs. baseline contrast, and reliable participant‐specific variance was observed in visual, motor, parietal, and prefrontal cortices (Figure 2b). Task vs. baseline ICCs positively correlated with both mean tSNR (*r* = .84) and the task versus baseline activity at the first session (*r* = .65).

Next, two task contrasts of interest were examined (Figure 2c). The linear slope across face‐morphs (coding emotion intensity from angry to happy) reliably tracked motor response in the bilateral motor cortex. Average linear slope activity at the first session was correlated with the associated reliability map (*r* = .61).

For the quadratic slope across morphs (coding ambiguity from ambiguous to overt), reliable signal (ICC > .4) overlapped with regions of significant activation at the first session in the bilateral dlPFC/anterior insula, supplementary motor area/anterior cingulate cortex (ACC). Average quadratic slope activity at the first session was correlated with the associated reliability map (*r* = .48).

Additional contrasts (i.e., difference value: ambiguous vs. overt faces, happy vs. angry faces) and tables detailing group‐level activation at the first scanning session and clusters of ICC > .4 are presented in Figure S2.

##### ROI‐based analysis

Similar test‐restest results were observed in Bayesian multi‐level analyses of ROIs covering the whole‐brain for both tasks. Figure 3b,c displays surface renderings of maps of ROI ICC for each contrast. Refer to Tables S12 and S14 for the full list of ICCs for each of the 200 cortical parcels and 14 subcortical ROIs.

## 4 DISCUSSION

This study examined test–retest reliability of neural responses during two face‐emotion paradigms, one requiring explicit, task‐directed face‐emotion labeling, and one involving implicit, task‐irrelevant face‐emotion processing. Three key findings emerged. First, scanner effects accounted for minimal variance in temporal signal‐to‐noise ratio (tSNR) and fMRI activity maps. Second, regions showing significant task‐contrast activity showed higher reliability than regions that did not show strong task‐related activity at the group level. Finally, across both tasks, we found greater reliability for task contrasts involving conditions with clearly distinct visual stimuli and associated cognitive demands (e.g., face vs. non‐face discrimination) compared to conditions with more similar demands (e.g., angry vs. happy discrimination).

Variability in tSNR and activation across scanners is undesirable for multi‐scanner/multi‐site studies. Previous work has generally reported relatively little systematic variability in fMRI signal across scanners (Noble et al., 2017), specifically in subtraction contrasts (Nielson et al., 2018). However, some studies combining data across scanners of different field strength and/or from different vendors or models find larger scanner effects (Friedman, Glover, Krenz, Magnotta, & First, 2006). Although we found substantial white matter variance to be scanner‐specific, we found little variance accounted for by scanner in gray matter. Our study employed scanners from the same vendor as is typical for single‐site or harmonized multi‐site studies; nonetheless, it is likely that effects would be larger for studies with less consistent hardware. Continuing to examine possible systematic scanner differences is important as differences may also be vendor‐specific. Different software solutions can be adopted to harmonize systematic scanner differences without removing other variance of interest. Alternatively, including scanner as a covariate can help partition scanner‐associated variance.

Past studies have typically found reliability estimates of task‐based imaging to be relatively poor across commonly used tasks (e.g., ICCs < .4; Elliott et al., 2019), despite robust group‐level activation. Using two face‐emotion studies, we partially confirmed patterns of low reliability in some of the key regions for face‐emotion studies (e.g., amygdala, subgenual ACC). Though not always the case, regions that are robustly activated at the group‐average level tend to exhibit less between‐individual variability, and problematically, this variability is not stable within an individual over time (i.e., activity is robust but not reliable). In contrast, other regions may show stronger between‐individual differences (leading to lower/less robust mean signal) that allows for variability that is consistent across time (Chen et al., 2021b; Hedge, Powell, & Sumner, 2018). Hence, sub‐optimal levels of reliability may, for some tasks, derive from design features that aim to maximize group‐level activation (thereby implicitly minimizing individual differences; Hedge et al., 2018; Lissek, Pine, & Grillon, 2006).

Herein, we do see moderate positive voxel‐wise correlations between reliability and group‐average task activation. In particular, contrasts with more dissimilar visual and cognitive demands (face vs. scramble control, ambiguous vs. overt faces) elicited more extensive and reliable activation. Note that activity–reliability correlations are in part driven by voxels that are neither activated nor reliable in any given contrast. Also, we identified some regions that did not show strong group‐level activation but did show participant‐level reliability; such regions may represent new candidates for individual differences research.

Low reliability estimates may also reflect analytic decisions, like contrast construction. Previous work has shown that constructing first‐level subtraction contrasts/difference scores can significantly impact reliability. Typically, there are high correlations between brain activation to different task conditions, often in key face‐emotion regions, such as the amygdala (activation to face and shape conditions correlating at >.9; Infantolino, Luking, Sauder, Curtin, & Hajcak, 2018). Thus, these contrasts can subtract out reliable participant variance shared among conditions. Although we focus on fMRI outcomes, this issue also impacts the reliability of behavioral difference score, such as RT. In our data, RT difference scores (e.g., for face vs. scramble control) showed poor stability, while most other measures (slopes and logistic regressors) showed moderate stability. The magnitude of the ICC for the behavioral metric did not appear to be indicative of ICC values of associated fMRI contrast activity.

The current study included two approaches to calculating reliability estimates for the whole brain: voxel‐wise linear mixed‐effects models and Bayesian hierarchical modeling of ROIs across the whole brain. The latter approach has several noteworthy advantages. First, we leverage Bayesian hierarchical modeling to examine many ROIs in one model, allowing for whole‐brain coverage while mitigating multiple testing. This is an advance from previous studies that often focused on a smaller number of a priori regions to minimize correction for multiple comparisons. Second, the Bayesian approach generates uncertainty estimates and standard deviations based on the posterior sample draws in contrast to frequentist linear mixed‐effects models, with which it can be difficult to assign adequate degrees of freedom. Although using an a priori whole‐brain atlas allows us to define ROIs independent of the current data and avoid circularity, a predefined anatomical or parcellation atlas may not best capture the most reliable functional units for a given task (and estimates may vary based on the chosen atlas). Nonetheless, our two statistical approaches largely converged, but ICCs were generally higher in the voxel‐wise approach. This is in part due to a “global calibration” across spatial units in the hierarchical model. Leveraging the distribution of estimates across ROIs helps to minimize outlier values and ideally yields a better estimate of true reliability. In our data, this partial pooling/shrinkage generally decreased regional ICC estimates given overall low reliability estimates across the brain.

Studying individual differences in a resource‐intensive setting, such as fMRI, can be challenging. Previous work has shown how first‐level model specifications (e.g., hemodynamic response function and noise correction methods; Fournier, Chase, Almeida, & Phillips, 2014) can affect reliability estimates. Herein, we suggest some additional measurement and statistical approaches that may help increase reliability of extracted metrics in existing paradigms. First, though many studies rely on subtraction contrasts (often to minimize statistical complexity and computational requirements), these can remove reliable participant variance. Instead, modeling conditions as a factor with multiple levels within a repeated‐measures ANOVA or a linear mixed‐effects framework can help retain reliable condition‐shared variance. Furthermore, trial‐wise modeling approaches using amplitude modulation or hierarchical models (Chen et al., 2021) can also be helpful in modeling cognitive processes precisely and circumvent subtraction contrasts. Additional modeling approaches, including structural equation (Cooper, Jackson, Barch, & Braver, 2019) and computational modeling, are growing and hold promise to increase reliability. For new data collection, it will be important to make design choices to improve reliability, for example by increasing the potency of stimuli or making conditions more distinct (i.e., less correlated), while still isolating the cognitive process of interest. Additionally, selecting sequences with improved tSNR will result in increased statistical power; similarly, increasing the number of trials can optimize the statistical efficiency of designs (Chen et al., 2021c).

This study provides strong data on reliability in healthy adults and has several strengths. These include assessing reliability across three points in time, which allowed us to separate scanner‐ and time‐related variance components. We also examine two different face‐emotion tasks requiring different attentional demands, and we compare two statistical approaches to reliability estimates. However, the current study also has several limitations. First, the generalizability of these estimates will need to be tested in individuals with psychopathology and pediatric samples. Second, there was variability in the time between sessions across participants, though this was constrained to 2–6 weeks. This timeframe would be similar to pre‐/post‐scanning for many psychiatric treatment trials, but reliability over longer time frames would still need to be examined, for example, to match developmental studies over years. Third, our sample size was larger than many reliability studies but is still relatively limited, especially given only moderately reliable behavioral effects. Fourth, scanners were of the same build and tasks were run with identical sequences across scanners to minimize scanner‐related variance. This reflects an ideal scenario and may not be the case for many large multi‐site projects.

The current report adds to the small but growing corpus of work on test–retest reliability of task‐based fMRI activation by examining the influence of specific scanners and task‐related factors on estimates of reliability. Greater reliability was found in regions activated during the task at the group level and for contrasts involving conditions with clearly distinct cognitive demands. This work highlights the importance of assessing reliability in the context of task activation patterns and specific task contrasts.

## CONFLICT OF INTEREST

The authors have no commercial or financial involvements that might present a conflict of interest.

## ETHICS STATEMENT

This research was conducted in compliance with the Declaration of Helsinki code of ethics and the standards established by the National Institute of Mental Health's Institutional Review Board. Informed consent was obtained from all participants.

## Supporting information


**Appendix S1** Supplementary Information

## Data Availability

The data that support the findings of this study are available from the corresponding author upon reasonable request.
