# Supplementary material for: Reliability of task‐evoked neural activation during face‐emotion paradigms: Effects of scanner and psychological processes
Source: Hum Brain Mapp. 2022 Feb 15;43(7):2109–20. doi: 10.1002/hbm.25723 (PMC8996353; doi:10.1002/hbm.25723)
Supplement: Supplementary file 1 — Appendix S1 Supplementary Information [file HBM-43-2109-s001.docx]

Supplementary Materials for

**Reliability of Task-Evoked Neural Activation during Face-Emotion Paradigms: Effects of Scanner and Psychological Processes**


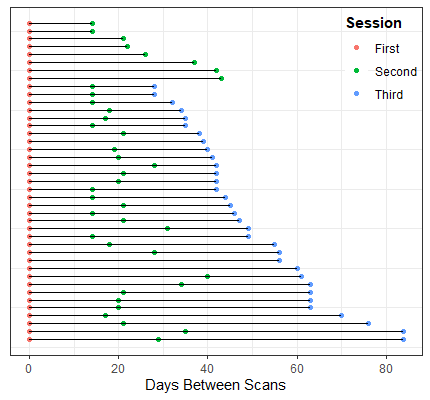


*Figure S1.* Timing of scanning sessions across participants.

Scanning sessions were 2-6 weeks apart. The final number of participants included in all analyses for the visual search task was *N*[Session 1-3]=40-35-33 and for the emotion labeling task: *N*[Session 1-3]=29-33-30. Here, we depict the timing in days between scan sessions for all participants, sorted by the number of scan sessions completed and the overall time between scans.

*Table S1.* Reliability of visual search task behavior.

| **Visual Search Contrasts** | **ICC** |
| --- | --- |
| Mean Reaction Time: 30-1 | 0.80 |
| Mean Reaction Time: Face-Scramble | 0.12 |
| Mean Reaction Time: Angry-Happy | 0.15 |
| Mean Reaction Time: Angry-Happy 30-1 | 0.08 |
| Mean Reaction Time: Face-Scramble 30-1 | 0.41 |
| Accuracy 30-1 | 0.47 |
| Mean Reaction Time: Slope Angry | 0.51 |
| Mean Reaction Time: Slope Happy | 0.63 |
| Mean Reaction Time: Slope Scramble | 0.63 |
| Mean Reaction Time: Slope Overall | 0.77 |
| Mean Reaction Time: Slope Angry log | 0.57 |
| Mean Reaction Time: Slope Happy log | 0.64 |
| Mean Reaction Time: Slope Scramble log | 0.68 |
| Mean Reaction Time: Slope Overall log | 0.83 |
| Mean Overall Reaction Time | 0.84 |

*Note*. 30-1: Search Array 30 distractors vs Search Array 1 distractor; ICC = intra-class correlation coefficient (participant-specific variance)

*Table S2.* Reliability of face-emotion labeling task behavior.

| **Face-Emotion Labeling Contrasts** | **ICC** |
| --- | --- |
| Linear Slope | 0.55 |
| Quadratic Slope | 0.59 |
| Logistic curve: Inflection Point | 0.55 |
| Logistic curve: Slope | 0.46 |
| Mean Reaction Time: Happy-Angry Faces | 0.37 |
| Mean Reaction Time: Ambiguous-Overt Faces | 0.51 |
| Mean Reaction Time: Angry Faces | 0.71 |
| Mean Reaction Time: Happy Faces | 0.84 |
| Mean Reaction Time: Ambiguous Faces | 0.76 |
| Mean Reaction Time: Overtly Emotional Faces | 0.84 |

Note. Angry faces: two most angry morphs; Happy faces: two most happy morphs; Ambiguous faces: middle three most ambiguous morphs; Overt faces: both most angry and most happy faces (i.e., four morphs); ICC = intra-class correlation coefficient (participant-specific variance)


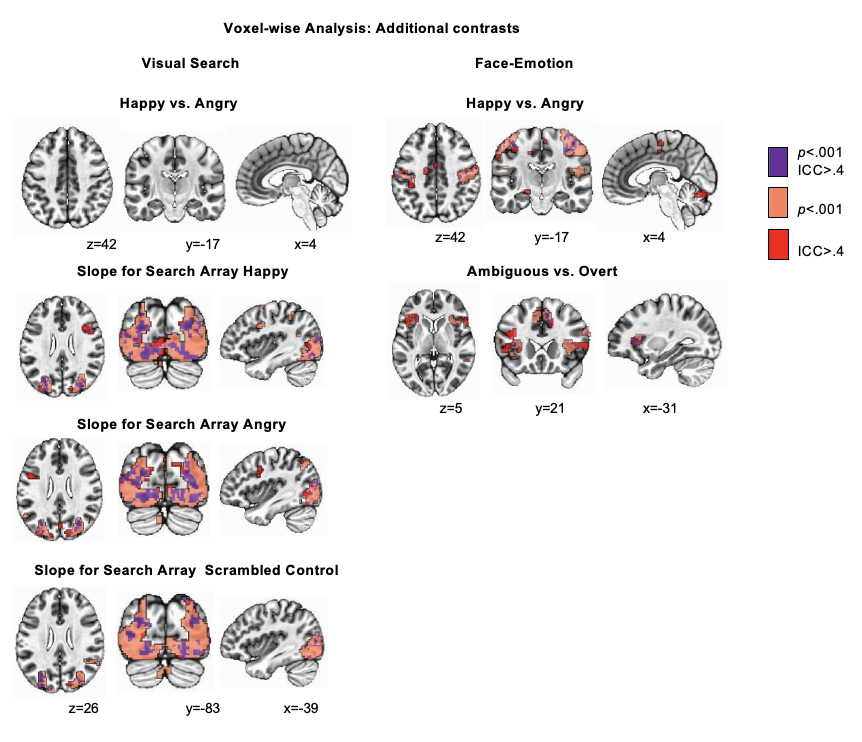


*Figure S2.* Voxel-wise conjunction maps of active and reliable regions.

These conjunction maps display the spatial overlap (purple) between reliable regions at an ICC>.4 threshold (red), regions activated at the first session corrected at voxel-wise *p*<.001, k>20 (peach).

*Table S3.* Summary of intraclass correlation coefficients (ICC>.4) for the linear slope for the face-emotion labeling task.

| Linear Slope Contrast | | | | | | | | | | | |
| --- | --- | --- | --- | --- | --- | --- | --- | --- | --- | --- | --- |
|  | Cluster Size | | Coordinates (center of mass) | | | Coordinates (at peak) | | |  |  |  |
| Region | k | mm3 | CM LR | CM  PA | CM  IS | MI LR | MI PA | MI  IS | Mean ICC | SEM  ICC | Max  ICC |
| L Postcentral Gyrus | 915 | 14297 | -40.9 | -24.1 | 55.3 | -32.5 | -26.8 | 48 | 0.5314 | 0.0032 | 0.7935 |
| R Precentral Gyrus | 766 | 11969 | 41.1 | -18.4 | 56 | 55 | -19.2 | 55.5 | 0.5033 | 0.0027 | 0.8079 |
| R SMA | 149 | 2328 | 1.7 | -8.8 | 56 | 10 | -9.2 | 58 | 0.4812 | 0.0049 | 0.6407 |
| L Postcentral Gyrus | 107 | 1672 | -54.5 | -19.1 | 20.1 | -55 | -19.2 | 23 | 0.5056 | 0.0077 | 0.7291 |
| R Cerebellum | 93 | 1453 | 18.9 | -58.7 | -19 | 22.5 | -59.2 | -22 | 0.4496 | 0.0038 | 0.5644 |
| L Cerebellum | 75 | 1172 | -10.2 | -63.2 | -15.6 | -12.5 | -61.8 | -17 | 0.4995 | 0.0082 | 0.6991 |
| L Postcentral Gyrus | 39 | 609 | -27.3 | -42.5 | 66.6 | -27.5 | -44.2 | 70.5 | 0.46 | 0.0093 | 0.6536 |
| L SMA | 34 | 531 | -5.6 | 0.2 | 45.9 | -10 | 3.2 | 45.5 | 0.4684 | 0.0089 | 0.5805 |
| R Postcentral Gyrus | 29 | 453 | 30.8 | -44.5 | 63.8 | 32.5 | -44.2 | 65.5 | 0.4692 | 0.0079 | 0.5791 |
| R Supramarginal Gyrus | 28 | 438 | 46.7 | -31.2 | 23.9 | 50 | -34.2 | 20.5 | 0.4598 | 0.007 | 0.543 |
| L Paracentral Lobule | 28 | 438 | -5.1 | -22 | 56.6 | -5 | -19.2 | 60.5 | 0.4725 | 0.0102 | 0.6241 |
| L Middle Frontal Gyrus | 27 | 422 | -48.1 | 12.6 | 40.6 | -47.5 | 10.8 | 40.5 | 0.4426 | 0.0075 | 0.5307 |
| L Precentral Gyrus | 21 | 328 | -52.9 | 5.2 | 41.3 | -55 | 5.8 | 43 | 0.4594 | 0.0106 | 0.6253 |
| R Superior Parietal Lobule | 20 | 313 | 31.2 | -54.9 | 66.6 | 35 | -54.2 | 68 | 0.4698 | 0.0112 | 0.5648 |

*Note*: Cluster-corrected voxel-wise linear mixed-effects model results are presented here summarizing regions with intra-class correlation coefficients (ICC>.4) for participant-specific variance, i.e., at least “fair” test-retest reliability. k=number of voxels in cluster, mm3=cluster volume, CM=center of mass of cluster, MI=max intensity (peak), SEM=standard error of the mean, LR=left-right (x), PA=posterior-anterior (y), IS=inferior-superior (z)

*Table S4.* Summary of intraclass correlation coefficients (ICC>.4) for task contrasts for quadratic slope contrast in the face-emotion labeling task.

| Quadratic Slope Contrast | | | | | | | | | | | |
| --- | --- | --- | --- | --- | --- | --- | --- | --- | --- | --- | --- |
|  | Cluster Size | | Coordinates (center of mass) | | | Coordinates (at peak) | | |  |  |  |
| Region | k | mm3 | CM LR | CM  PA | CM  IS | MI LR | MI  PA | MI  IS | Mean ICC | SEM ICC | Max ICC |
| R Superior Medial Gyrus | 87 | 1359 | 6.9 | 24.3 | 42.2 | 7.5 | 23.2 | 45.5 | 0.4618 | 0.0048 | 0.5855 |
| R Inferior Frontal Gyrus | 79 | 1234 | 48.2 | 25.6 | 5.2 | 55 | 18.2 | 5.5 | 0.4618 | 0.0048 | 0.5981 |
| R Inferior Frontal Gyrus | 70 | 1094 | 39.5 | 12.8 | 31.6 | 35 | 13.2 | 30.5 | 0.4578 | 0.0057 | 0.5949 |
| L Precentral Gyrus | 61 | 953 | -41.1 | 8.4 | 46 | -42.5 | 10.8 | 40.5 | 0.4629 | 0.0064 | 0.6446 |
| L SMA | 58 | 906 | -5.6 | 19.1 | 53.2 | -5 | 13.2 | 58 | 0.4554 | 0.005 | 0.534 |
| R Superior Parietal Lobule | 49 | 766 | 27.5 | -59.6 | 48.9 | 27.5 | -59.2 | 48 | 0.4819 | 0.0089 | 0.6548 |
| L Inferior Frontal Gyrus | 44 | 688 | -44.9 | 18.7 | 5.2 | -45 | 15.8 | 3 | 0.4601 | 0.0077 | 0.5653 |
| L Postcentral Gyrus | 44 | 688 | -37.2 | -26.2 | 52 | -35 | -29.2 | 55.5 | 0.4742 | 0.0072 | 0.5617 |
| L Insula Lobe | 43 | 672 | -31.2 | 27.8 | 3.7 | -32.5 | 28.2 | 8 | 0.4684 | 0.0065 | 0.6011 |
| L Inferior Frontal Gyrus | 39 | 609 | -55.2 | 16.8 | 14.7 | -60 | 15.8 | 18 | 0.4597 | 0.0084 | 0.6432 |
| L Precentral Gyrus | 36 | 563 | -39.9 | 4.5 | 32.6 | -42.5 | -6.8 | 30.5 | 0.4382 | 0.0051 | 0.546 |
| R Superior Medial Gyrus | 34 | 531 | 7.1 | 30.3 | 58.5 | 5 | 30.8 | 58 | 0.4651 | 0.0113 | 0.6577 |
| R Precentral Gyrus | 21 | 328 | 36.4 | -1.4 | 49.3 | 37.5 | -1.8 | 50.5 | 0.4562 | 0.0091 | 0.5621 |

*Note*: Cluster-corrected voxel-wise linear mixed-effects model results are presented here summarizing regions with intra-class correlation coefficients (ICC>.4) for participant-specific variance, i.e., at least “fair” test-retest reliability. k=number of voxels in cluster, mm3=cluster volume, CM=center of mass of cluster, MI=max intensity (peak), SEM=standard error of the mean, LR=left-right (x), PA=posterior-anterior (y), IS=inferior-superior (z)

*Table S5.* Summary of intraclass correlation coefficients (ICC>.4) for the log-transformed slope for the visual search task.

| Log-transformed Slope | | | | | | | | | | | |
| --- | --- | --- | --- | --- | --- | --- | --- | --- | --- | --- | --- |
|  | Cluster Size | | Coordinates (Center of Mass) | | | Coordinates (at peak) | | |  |  |  |
| Region | k | mm3 | CM LR | CM  PA | CM  IS | MI  LR | MI  PA | MI  IS | Mean ICC | SEM ICC | Max ICC |
| L Middle Occipital Gyrus | 6535 | 102109 | -1.2 | -77.1 | 14.7 | -25 | -64.2 | 33 | 0.5427 | 0.0012 | 0.8295 |
| L Precentral Gyrus | 380 | 5938 | -41.9 | 2.7 | 39.6 | -45 | 3.2 | 25.5 | 0.501 | 0.0036 | 0.6908 |
| R Inferior Frontal Gyrus | 237 | 3703 | 42.2 | 11.4 | 29.1 | 45 | 8.2 | 23 | 0.5115 | 0.0048 | 0.7244 |
| R Precentral Gyrus | 73 | 1141 | 39 | -1.9 | 50.5 | 35 | -1.8 | 48 | 0.4855 | 0.007 | 0.6526 |
| R Inferior Temporal Gyrus | 37 | 578 | 50 | -58.7 | -9 | 47.5 | -59.2 | -12 | 0.4439 | 0.0051 | 0.5056 |
| L Middle Temporal Gyrus | 25 | 391 | -54.1 | -64.8 | 19.8 | -55 | -64.2 | 18 | 0.4641 | 0.0112 | 0.5813 |
| L Cuneus | 22 | 344 | 1.6 | -71.6 | 24.1 | 2.5 | -71.8 | 25.5 | 0.4748 | 0.013 | 0.6507 |

*Note*: Cluster-corrected voxel-wise linear mixed-effects model results are presented here summarizing regions with intra-class correlation coefficients (ICC>.4) for participant-specific variance, i.e., at least “fair” test-retest reliability. k=number of voxels in cluster, mm3=cluster volume, CM=center of mass of cluster, MI=max intensity (peak), SEM=standard error of the mean, LR=left-right (x), PA=posterior-anterior (y), IS=inferior-superior (z)

*Table S6.* Summary of intraclass correlation coefficients (ICC>.4) for the Face vs. Scramble control contrast for the visual search task.

| Face vs. Scramble Contrast | | | | | | | | | | | |
| --- | --- | --- | --- | --- | --- | --- | --- | --- | --- | --- | --- |
|  | Cluster Size | | Coordinates (Center of Mass) | | | Coordinates (at peak) | | |  |  |  |
| Region | k | mm3 | CM  LR | CM  PA | CM  IS | MI LR | MI PA | MI IS | Mean ICC | SEM | Max ICC |
| R Fusiform Gyrus | 609 | 9516 | 40.8 | -64.3 | -11 | 42.5 | -54.2 | -14.5 | 0.5218 | 0.004 | 0.8163 |
| L Fusiform Gyrus | 395 | 6172 | -40.1 | -65.6 | -14.2 | -37.5 | -56.8 | -17 | 0.5148 | 0.0038 | 0.7719 |
| R Superior Occipital Gyrus | 20 | 313 | 25.9 | -92.8 | 17.1 | 25 | -94.2 | 18 | 0.4614 | 0.0099 | 0.5766 |

*Note*: Cluster-corrected voxel-wise linear mixed-effects model results are presented here summarizing regions with intra-class correlation coefficients (ICC>.4) for participant-specific variance, i.e., at least “fair” test-retest reliability. k=number of voxels in cluster, mm3=cluster volume, CM=center of mass of cluster, MI=max intensity (peak), SEM=standard error of the mean, LR=left-right (x), PA=posterior-anterior (y), IS=inferior-superior (z)

*Table S7.* Summary of group-level activation for the linear slope for the face-emotion labeling task.

| Linear Slope Contrast | | | | | | | | | | | |
| --- | --- | --- | --- | --- | --- | --- | --- | --- | --- | --- | --- |
|  | Cluster Size | | Coordinates (Center of Mass) | | | Coordinates (at peak) | | |  |  |  |
| Region | k | mm3 | CM LR | CM PA | CM IS | MI  LR | MI PA | MI  IS | Mean F | SEM F | Max Intensity |
| R Postcentral Gyrus | 2246 | 35094 | 41.9 | -20.9 | 52.6 | 40 | -19.2 | 53 | 52.838 | 1.0382 | 245.45 |
| L Postcentral Gyrus | 1602 | 25031 | -40.6 | -24.6 | 56.9 | -40 | -21.8 | 55.5 | 46.843 | 1.0502 | 239.34 |
| L Cerebellum | 622 | 9719 | -16.4 | -59.4 | -19.5 | -17.5 | -56.8 | -19.5 | 37.979 | 1.147 | 156.62 |
| R Cerebellum | 439 | 6859 | 17.3 | -56.9 | -19 | 15 | -56.8 | -19.5 | 38.295 | 1.4063 | 154.83 |
| R SMA | 241 | 3766 | 6.7 | -8.4 | 52.6 | 5 | -4.2 | 53 | 17.82 | 0.3782 | 39.932 |
| L Rolandic Operculum | 136 | 2125 | -48.3 | -20.4 | 21.3 | -52.5 | -19.2 | 23 | 21.389 | 0.8783 | 56.042 |
| L SMA | 119 | 1859 | -6.6 | -14.7 | 50.7 | -5 | -6.8 | 53 | 16.359 | 0.3552 | 28.037 |
| R Cerebellum | 99 | 1547 | 15.5 | -67.4 | -40.8 | 12.5 | -69.2 | -42 | 22.454 | 1.053 | 57.024 |
| R Thalamus | 95 | 1484 | 15 | -21.3 | 4.2 | 15 | -21.8 | 5.5 | 20.344 | 0.9463 | 45.591 |
| R Rolandic Operculum | 70 | 1094 | 40.8 | -0.5 | 14.3 | 42.5 | 0.8 | 15.5 | 20.671 | 1.0201 | 40.954 |
| L Putamen | 21 | 328 | -32.4 | -11.9 | 1.8 | -32.5 | -11.8 | 0.5 | 14.234 | 0.5525 | 19.687 |
| L Thalamus | 20 | 313 | -16.5 | -24 | 3.4 | -17.5 | -24.2 | 0.5 | 15.965 | 0.9018 | 24.359 |

*Note*: Cluster-corrected voxel-wise results are presented here, summarizing regions that are significantly activated at the first scan session across all participants. k=number of voxels in cluster, mm3=cluster volume, CM=center of mass of cluster, MI=max intensity (peak), SEM=standard error of the mean, LR=left-right (x), PA=posterior-anterior (y), IS=inferior-superior (z)

*Table S8.* Summary of group-level activation for the quadratic slope for the face-emotion labeling task.

| Quadratic Slope Contrast | | | | | | | | | | | |
| --- | --- | --- | --- | --- | --- | --- | --- | --- | --- | --- | --- |
|  | Cluster Size | | Coordinates (Center of Mass) | | | Coordinates (at peak) | | |  |  |  |
| Region | k | mm3 | CM LR | CM PA | CM IS | MI LR | MI PA | MI IS | Mean F | SEM F | Max Intensity |
| L SMA | 646 | 10094 | 1.3 | 22.8 | 46.8 | 7.5 | 20.8 | 45.5 | 21.246 | 0.3463 | 63.674 |
| R Inferior Frontal Gyrus | 457 | 7141 | 47 | 18.2 | 27.6 | 47.5 | 10.8 | 25.5 | 16.454 | 0.1986 | 32.165 |
| L Angular Gyrus | 337 | 5266 | -45.3 | -67.8 | 31.7 | -40 | -74.2 | 38 | 15.469 | 0.2036 | 28.916 |
| R Insula Lobe | 327 | 5109 | 37 | 22.6 | 5 | 32.5 | 23.2 | 10.5 | 20.985 | 0.4326 | 43.887 |
| L Insula Lobe | 256 | 4000 | -34.2 | 22.5 | 5.1 | -30 | 20.8 | 10.5 | 18.971 | 0.4396 | 45.326 |
| L Middle Frontal Gyrus | 164 | 2563 | -24.7 | 24.8 | 43.9 | -27.5 | 20.8 | 40.5 | 14.878 | 0.2299 | 26.278 |
| L Precentral Gyrus | 83 | 1297 | -42.7 | 6.1 | 31.8 | -42.5 | 0.8 | 30.5 | 14.021 | 0.2879 | 22.659 |
| L Mid Orbital Gyrus | 72 | 1125 | -6 | 50.5 | -4.9 | -5 | 50.8 | -4.5 | 14.344 | 0.3265 | 21.531 |
| R Angular Gyrus | 58 | 906 | 48.1 | -60 | 22.9 | 42.5 | -59.2 | 23 | 14.112 | 0.313 | 21.671 |
| L Middle Cingulate Cortex | 52 | 813 | -9 | -42.4 | 37.8 | -12.5 | -44.2 | 38 | 14.87 | 0.4119 | 21.586 |
| L Superior Frontal Gyrus | 41 | 641 | -13.9 | 69.8 | 10.3 | -15 | 68.2 | 8 | 14.419 | 0.3933 | 18.613 |
| L Precuneus | 40 | 625 | -6.9 | -57.1 | 30.9 | -7.5 | -56.8 | 33 | 13.182 | 0.2838 | 18.903 |
| R Inferior Occipital Gyrus | 32 | 500 | 50.5 | -74.1 | -3.6 | 50 | -74.2 | -4.5 | 14.748 | 0.4968 | 21.885 |
| R Middle Occipital Gyrus | 26 | 406 | 39.6 | -80.4 | 13 | 40 | -79.2 | 10.5 | 12.452 | 0.2432 | 15.486 |
| R Middle Frontal Gyrus | 26 | 406 | 23.7 | 28 | 41.3 | 22.5 | 25.8 | 38 | 12.415 | 0.2064 | 15.197 |
| R Middle Occipital Gyrus | 20 | 313 | 44.7 | -74 | 31.6 | 45 | -74.2 | 33 | 12.564 | 0.358 | 16.29 |

*Note*: Cluster-corrected voxel-wise results are presented here summarizing regions that are significantly activated at the first scan session across all participants. k=number of voxels in cluster, mm3=cluster volume, CM=center of mass of cluster, MI=max intensity (peak), SEM=standard error of the mean, LR=left-right (x), PA=posterior-anterior (y), IS=inferior-superior (z)

*Table S9.* Summary of group-level activation for the Face vs. Scramble contrast for the visual search task.

| Face vs. Scramble Contrast | | | | | | | | | | | |
| --- | --- | --- | --- | --- | --- | --- | --- | --- | --- | --- | --- |
|  | Cluster Size | | Coordinates (Center of Mass) | | | Coordinates (at peak) | | |  |  |  |
| Region | k | mm3 | CM LR | CM PA | CM IS | MI LR | MI PA | MI IS | Mean F | SEM F | Max Intensity |
| R Inferior Occipital Gyrus | 830 | 12969 | 40.9 | -65.8 | -13.4 | 42.5 | -56.8 | -19.5 | 25.301 | 0.5924 | 89.248 |
| L Fusiform Gyrus | 617 | 9641 | -41.7 | -69.3 | -12.9 | -42.5 | -64.2 | -17 | 19.072 | 0.4053 | 49.724 |
| R Hippocampus | 49 | 766 | 20 | -6.2 | -14.9 | 20 | -9.2 | -14.5 | 9.7335 | 0.2769 | 14.655 |
| R Middle Temporal Gyrus | 26 | 406 | 61.8 | -53.6 | 11.7 | 62.5 | -49.2 | 13 | 9.1371 | 0.3388 | 13.29 |

*Note*: Cluster-corrected voxel-wise results are presented here, summarizing regions that are significantly activated at the first scan session across all participants. k=number of voxels in cluster, mm3=cluster volume, CM=center of mass of cluster, MI=max intensity (peak), SEM=standard error of the mean, LR=left-right (x), PA=posterior-anterior (y), IS=inferior-superior (z)

*Table S10.* Summary of group-level activation for the log-transformed slope for the visual search task.

| Log-transformed Slope Contrast | | | | | | | | | | | |
| --- | --- | --- | --- | --- | --- | --- | --- | --- | --- | --- | --- |
|  | Cluster Size | | Coordinates (Center of Mass) | | | Coordinates (at peak) | | |  |  |  |
| Region | k | mm3 | CM LR | CM PA | CM IS | MI LR | MI PA | MI IS | Mean F | SEM F | Max Intensity |
| L Middle Occipital Gyrus | 13858 | 216531 | 0.3 | -79.2 | 8.4 | 25 | -81.8 | -9.5 | 62.234 | 0.6271 | 550.01 |
| L Precentral Gyrus | 888 | 13875 | -38.3 | 0 | 44.5 | -42.5 | 3.2 | 30.5 | 20.023 | 0.3858 | 61.913 |
| L Precuneus | 517 | 8078 | -1.2 | -64.1 | 30.5 | 5 | -56.8 | 28 | 9.702 | 0.0911 | 18.347 |
| R Superior Frontal Gyrus | 517 | 8078 | 33 | -1.5 | 54.3 | 40 | -1.8 | 50.5 | 24.144 | 0.6732 | 74.574 |
| R Inferior Frontal Gyrus | 386 | 6031 | 46.1 | 9.5 | 30.5 | 47.5 | 10.8 | 28 | 17.793 | 0.467 | 50.184 |
| L SMA | 375 | 5859 | 1.7 | 12.9 | 49.4 | 2.5 | 13.2 | 50.5 | 13.272 | 0.2844 | 32.384 |
| R Thalamus | 310 | 4844 | 4 | -29.3 | -2.3 | 20 | -29.2 | -2 | 15.204 | 0.5411 | 70.013 |
| R Calcarine Gyrus | 261 | 4078 | 26.6 | -48.4 | 12 | 22.5 | -46.8 | 13 | 10.802 | 0.2336 | 30.926 |
| R SupraMarginal Gyrus | 186 | 2906 | 55.6 | -27.6 | 23.4 | 47.5 | -34.2 | 23 | 9.0825 | 0.1219 | 14.779 |
| R Insula Lobe | 151 | 2359 | 35.1 | 21.8 | 4.5 | 32.5 | 23.2 | 8 | 12.134 | 0.3487 | 24.773 |
| R ParaHippocampal Gyrus | 100 | 1563 | 17.3 | -23.7 | -16.7 | 15 | -21.8 | -22 | 10.304 | 0.2467 | 17.88 |
| R Cerebellum | 88 | 1375 | 26.1 | -82.4 | -33.7 | 32.5 | -81.8 | -34.5 | 9.2271 | 0.162 | 13.611 |
| R Putamen | 68 | 1063 | 30.2 | 6.8 | -2.6 | 32.5 | 5.8 | -4.5 | 10.11 | 0.383 | 21.656 |
| L ParaHippocampal Gyrus | 64 | 1000 | -17.1 | -27.6 | -17.1 | -17.5 | -24.2 | -19.5 | 11.367 | 0.3943 | 20.959 |
| L Insula Lobe | 50 | 781 | -31 | 24.2 | 4 | -30 | 25.8 | 5.5 | 11.12 | 0.4635 | 19.454 |
| L Superior Temporal Gyrus | 47 | 734 | -43.6 | -4.5 | -10.7 | -42.5 | 0.8 | -14.5 | 9.6277 | 0.3024 | 14.198 |
| R Postcentral Gyrus | 46 | 719 | 24.9 | -43.9 | 70.1 | 25 | -44.2 | 70.5 | 10.273 | 0.3858 | 17.972 |
| R Superior Frontal Gyrus | 40 | 625 | 17.2 | 35.3 | 51.4 | 17.5 | 35.8 | 50.5 | 9.3017 | 0.2662 | 13.016 |
| Cerebellar Vermis | 30 | 469 | 0.2 | -60.8 | -35.7 | 0 | -56.8 | -34.5 | 10.469 | 0.5966 | 17.568 |
| L Superior Frontal Gyrus | 23 | 359 | -12 | 32.2 | 58.6 | -12.5 | 35.8 | 58 | 9.3358 | 0.3345 | 13.106 |

*Note*: Cluster-corrected voxel-wise results are presented here, summarizing regions that are significantly activated at the first scan session across all participants. k=number of voxels in cluster, mm3=cluster volume, CM=center of mass of cluster, MI=max intensity (peak), SEM=standard error of the mean, LR=left-right (x), PA=posterior-anterior (y), IS=inferior-superior (z)

*Table S11.* ROI-level reliability for the face-emotion tSNR and Task vs. Baseline: Bayesian multilevel model (BMM) and conventional linear mixed-effects model (ICC) for 200 cortical parcels and 14 subcortical ROIs (ICC: blue=<.4, red=.4-.6, orange=.6-.75, yellow=.75-1)

|  | **tSNR** | | | | | | | | **Task vs. Baseline** | | | | | | | |
| --- | --- | --- | --- | --- | --- | --- | --- | --- | --- | --- | --- | --- | --- | --- | --- | --- |
| **Label** | **BMM** | **SD** | **2.50%** | **5%** | **50%** | **95%** | **97.50%** | **ICC** | **BBM** | **SD** | **2.50%** | **5%** | **50%** | **95%** | **97.50%** | **ICC** |
| L VisCent ExStr 1 | 0.725 | 0.022 | 0.676 | 0.686 | 0.727 | 0.758 | 0.763 | 0.636 | 0.741 | 0.016 | 0.706 | 0.712 | 0.742 | 0.766 | 0.769 | 0.675 |
| L VisCent ExStr 2 | 0.767 | 0.019 | 0.727 | 0.734 | 0.769 | 0.795 | 0.8 | 0.517 | 0.772 | 0.015 | 0.74 | 0.746 | 0.773 | 0.794 | 0.798 | 0.676 |
| L VisCent Striate 1 | 0.746 | 0.02 | 0.701 | 0.71 | 0.747 | 0.775 | 0.779 | 0.695 | 0.892 | 0.006 | 0.878 | 0.881 | 0.892 | 0.901 | 0.903 | 0.794 |
| L VisCent ExStr 3 | 0.795 | 0.016 | 0.757 | 0.765 | 0.797 | 0.82 | 0.824 | 0.462 | 0.722 | 0.018 | 0.683 | 0.691 | 0.723 | 0.749 | 0.753 | 0.636 |
| L VisCent ExStr 4 | 0.751 | 0.02 | 0.709 | 0.716 | 0.752 | 0.779 | 0.784 | 0.338 | 0.604 | 0.024 | 0.554 | 0.561 | 0.605 | 0.641 | 0.647 | 0.558 |
| L VisCent ExStr 5 | 0.758 | 0.019 | 0.714 | 0.723 | 0.759 | 0.786 | 0.79 | 0.451 | 0.632 | 0.021 | 0.587 | 0.596 | 0.634 | 0.666 | 0.671 | 0.71 |
| L VisPeri ExStrInf 1 | 0.695 | 0.024 | 0.641 | 0.653 | 0.697 | 0.731 | 0.736 | 0.621 | 0.589 | 0.023 | 0.537 | 0.549 | 0.591 | 0.623 | 0.63 | 0.418 |
| L VisPeri ExStrInf 2 | 0.684 | 0.025 | 0.63 | 0.639 | 0.685 | 0.722 | 0.728 | 0.655 | 0.692 | 0.019 | 0.651 | 0.659 | 0.694 | 0.722 | 0.726 | 0.55 |
| L VisPeri ExStrInf 3 | 0.728 | 0.021 | 0.68 | 0.689 | 0.73 | 0.76 | 0.766 | 0.768 | 0.616 | 0.022 | 0.57 | 0.577 | 0.618 | 0.651 | 0.658 | 0.215 |
| L VisPeri StriCal 1 | 0.702 | 0.023 | 0.651 | 0.661 | 0.704 | 0.738 | 0.744 | 0.629 | 0.816 | 0.011 | 0.792 | 0.796 | 0.817 | 0.834 | 0.837 | 0.716 |
| L VisPeri ExStrSup 1 | 0.758 | 0.019 | 0.714 | 0.721 | 0.759 | 0.785 | 0.791 | 0.686 | 0.673 | 0.02 | 0.629 | 0.638 | 0.674 | 0.704 | 0.708 | 0.606 |
| L VisPeri ExStrSup 2 | 0.783 | 0.017 | 0.744 | 0.752 | 0.784 | 0.808 | 0.812 | 0.67 | 0.71 | 0.018 | 0.67 | 0.678 | 0.711 | 0.737 | 0.742 | 0.699 |
| L SomMotA 1 | 0.693 | 0.024 | 0.638 | 0.649 | 0.694 | 0.729 | 0.734 | 0.689 | 0.599 | 0.022 | 0.551 | 0.559 | 0.6 | 0.633 | 0.639 | 0.591 |
| L SomMotA 2 | 0.737 | 0.021 | 0.691 | 0.7 | 0.738 | 0.768 | 0.772 | 0.555 | 0.657 | 0.021 | 0.611 | 0.621 | 0.658 | 0.688 | 0.693 | 0.524 |
| L SomMotA 3 | 0.706 | 0.023 | 0.654 | 0.663 | 0.707 | 0.741 | 0.747 | 0.607 | 0.76 | 0.015 | 0.726 | 0.734 | 0.761 | 0.784 | 0.788 | 0.595 |
| L SomMotA 4 | 0.709 | 0.023 | 0.659 | 0.67 | 0.711 | 0.743 | 0.749 | 0.599 | 0.661 | 0.02 | 0.615 | 0.624 | 0.662 | 0.692 | 0.697 | 0.608 |
| L SomMotA 5 | 0.739 | 0.021 | 0.692 | 0.702 | 0.741 | 0.77 | 0.775 | 0.571 | 0.563 | 0.024 | 0.512 | 0.522 | 0.564 | 0.6 | 0.606 | 0.526 |
| L SomMotA 6 | 0.731 | 0.021 | 0.683 | 0.694 | 0.732 | 0.763 | 0.768 | 0.735 | 0.618 | 0.023 | 0.568 | 0.578 | 0.62 | 0.652 | 0.658 | 0.655 |
| L SomMotA 7 | 0.737 | 0.021 | 0.69 | 0.698 | 0.738 | 0.768 | 0.772 | 0.694 | 0.541 | 0.023 | 0.489 | 0.499 | 0.542 | 0.577 | 0.583 | 0.388 |
| L SomMotA 8 | 0.739 | 0.02 | 0.695 | 0.702 | 0.74 | 0.769 | 0.773 | 0.699 | 0.557 | 0.024 | 0.505 | 0.515 | 0.558 | 0.593 | 0.599 | 0.424 |
| L SomMotB Aud 1 | 0.728 | 0.021 | 0.68 | 0.689 | 0.73 | 0.761 | 0.766 | 0.777 | 0.563 | 0.024 | 0.514 | 0.523 | 0.565 | 0.6 | 0.607 | 0.339 |
| L SomMotB Aud 2 | 0.715 | 0.022 | 0.664 | 0.674 | 0.717 | 0.748 | 0.754 | 0.694 | 0.53 | 0.022 | 0.48 | 0.489 | 0.531 | 0.563 | 0.568 | 0.134 |
| L SomMotB S2 1 | 0.693 | 0.024 | 0.64 | 0.652 | 0.695 | 0.73 | 0.736 | 0.64 | 0.528 | 0.022 | 0.478 | 0.487 | 0.529 | 0.561 | 0.566 | 0.203 |
| L SomMotB S2 2 | 0.711 | 0.023 | 0.66 | 0.672 | 0.712 | 0.745 | 0.75 | 0.793 | 0.569 | 0.024 | 0.518 | 0.528 | 0.57 | 0.606 | 0.611 | 0.414 |
| L SomMotB Aud 3 | 0.712 | 0.023 | 0.663 | 0.672 | 0.714 | 0.746 | 0.752 | 0.659 | 0.56 | 0.023 | 0.512 | 0.52 | 0.561 | 0.597 | 0.602 | 0.315 |
| L SomMotB S2 3 | 0.739 | 0.021 | 0.693 | 0.701 | 0.741 | 0.77 | 0.775 | 0.765 | 0.589 | 0.023 | 0.539 | 0.55 | 0.589 | 0.624 | 0.63 | 0.345 |
| L SomMotB Cent 1 | 0.713 | 0.022 | 0.665 | 0.674 | 0.715 | 0.747 | 0.752 | 0.718 | 0.566 | 0.023 | 0.516 | 0.525 | 0.568 | 0.602 | 0.607 | 0.412 |
| L SomMotB Cent 2 | 0.697 | 0.024 | 0.645 | 0.655 | 0.699 | 0.733 | 0.739 | 0.64 | 0.57 | 0.024 | 0.517 | 0.527 | 0.571 | 0.607 | 0.613 | 0.485 |
| L DorsAttnA TempOcc 1 | 0.736 | 0.021 | 0.69 | 0.699 | 0.738 | 0.768 | 0.773 | 0.595 | 0.669 | 0.021 | 0.625 | 0.633 | 0.67 | 0.701 | 0.706 | 0.644 |
| L DorsAttnA TempOcc 2 | 0.737 | 0.021 | 0.691 | 0.7 | 0.739 | 0.767 | 0.772 | 0.445 | 0.633 | 0.022 | 0.585 | 0.593 | 0.634 | 0.668 | 0.673 | 0.612 |
| L DorsAttnA ParOcc 1 | 0.735 | 0.021 | 0.689 | 0.698 | 0.737 | 0.766 | 0.771 | 0.444 | 0.575 | 0.024 | 0.524 | 0.533 | 0.576 | 0.612 | 0.619 | 0.554 |
| L DorsAttnA SPL 1 | 0.731 | 0.021 | 0.684 | 0.694 | 0.733 | 0.764 | 0.769 | 0.518 | 0.581 | 0.024 | 0.529 | 0.539 | 0.583 | 0.618 | 0.623 | 0.621 |
| L DorsAttnA SPL 2 | 0.753 | 0.019 | 0.709 | 0.719 | 0.755 | 0.782 | 0.786 | 0.52 | 0.586 | 0.024 | 0.534 | 0.544 | 0.588 | 0.621 | 0.628 | 0.538 |
| L DorsAttnA SPL 3 | 0.761 | 0.019 | 0.721 | 0.727 | 0.762 | 0.79 | 0.794 | 0.529 | 0.65 | 0.021 | 0.607 | 0.614 | 0.651 | 0.683 | 0.689 | 0.635 |
| L DorsAttnB PostC 1 | 0.735 | 0.021 | 0.689 | 0.698 | 0.737 | 0.765 | 0.771 | 0.713 | 0.616 | 0.023 | 0.566 | 0.576 | 0.618 | 0.65 | 0.655 | 0.472 |
| L DorsAttnB PostC 2 | 0.734 | 0.021 | 0.687 | 0.697 | 0.736 | 0.765 | 0.77 | 0.581 | 0.612 | 0.022 | 0.564 | 0.574 | 0.613 | 0.647 | 0.653 | 0.455 |
| L DorsAttnB PostC 3 | 0.736 | 0.021 | 0.69 | 0.699 | 0.738 | 0.768 | 0.772 | 0.545 | 0.599 | 0.023 | 0.549 | 0.558 | 0.601 | 0.635 | 0.641 | 0.647 |
| L DorsAttnB PostC 4 | 0.771 | 0.018 | 0.73 | 0.738 | 0.773 | 0.798 | 0.802 | 0.609 | 0.542 | 0.023 | 0.491 | 0.501 | 0.543 | 0.577 | 0.583 | 0.44 |
| L DorsAttnB FEF 1 | 0.713 | 0.022 | 0.663 | 0.672 | 0.714 | 0.746 | 0.751 | 0.666 | 0.565 | 0.023 | 0.516 | 0.523 | 0.567 | 0.601 | 0.607 | 0.389 |
| L SalVentAttnA ParOper1 | 0.711 | 0.023 | 0.66 | 0.669 | 0.712 | 0.745 | 0.75 | 0.732 | 0.6 | 0.023 | 0.55 | 0.56 | 0.602 | 0.635 | 0.64 | 0.407 |
| L SalVentAttnA Ins 1 | 0.694 | 0.024 | 0.64 | 0.651 | 0.696 | 0.73 | 0.735 | 0.664 | 0.529 | 0.022 | 0.478 | 0.488 | 0.531 | 0.563 | 0.568 | 0.333 |
| L SalVentAttnA FrOper 1 | 0.725 | 0.022 | 0.677 | 0.687 | 0.727 | 0.758 | 0.763 | 0.774 | 0.57 | 0.023 | 0.521 | 0.527 | 0.571 | 0.606 | 0.611 | 0.373 |
| L SalVentAttnA FrOper 2 | 0.706 | 0.023 | 0.657 | 0.666 | 0.708 | 0.742 | 0.748 | 0.783 | 0.606 | 0.023 | 0.558 | 0.567 | 0.608 | 0.642 | 0.649 | 0.531 |
| L SalVentAttnA ParMed 1 | 0.709 | 0.023 | 0.659 | 0.668 | 0.711 | 0.743 | 0.747 | 0.693 | 0.529 | 0.022 | 0.479 | 0.488 | 0.531 | 0.562 | 0.567 | 0.325 |
| L SalVentAttnA FrMed 1 | 0.69 | 0.024 | 0.637 | 0.647 | 0.691 | 0.726 | 0.732 | 0.691 | 0.603 | 0.023 | 0.553 | 0.562 | 0.605 | 0.639 | 0.646 | 0.369 |
| L SalVentAttnA FrMed 2 | 0.734 | 0.021 | 0.689 | 0.698 | 0.736 | 0.766 | 0.771 | 0.761 | 0.678 | 0.021 | 0.634 | 0.642 | 0.679 | 0.71 | 0.715 | 0.537 |
| L SalVentAttnB IPL 1 | 0.747 | 0.02 | 0.704 | 0.712 | 0.748 | 0.777 | 0.781 | 0.691 | 0.584 | 0.023 | 0.534 | 0.543 | 0.585 | 0.619 | 0.626 | 0.571 |
| L SalVentAttnB PFCl 1 | 0.717 | 0.022 | 0.668 | 0.677 | 0.718 | 0.75 | 0.755 | 0.772 | 0.549 | 0.024 | 0.497 | 0.506 | 0.551 | 0.586 | 0.592 | 0.591 |
| L SalVentAttnB Ins 1 | 0.694 | 0.024 | 0.643 | 0.652 | 0.696 | 0.731 | 0.737 | 0.777 | 0.589 | 0.024 | 0.539 | 0.547 | 0.59 | 0.625 | 0.631 | 0.488 |
| L SalVentAttnB PFCmp 1 | 0.722 | 0.022 | 0.673 | 0.682 | 0.724 | 0.754 | 0.76 | 0.761 | 0.567 | 0.024 | 0.518 | 0.526 | 0.569 | 0.603 | 0.609 | 0.429 |
| L LimbicB OFC 1 | 0.764 | 0.018 | 0.723 | 0.731 | 0.765 | 0.791 | 0.796 | 0.85 | 0.563 | 0.024 | 0.511 | 0.521 | 0.564 | 0.599 | 0.605 | 0.488 |
| L LimbicB OFC 2 | 0.868 | 0.01 | 0.844 | 0.848 | 0.869 | 0.883 | 0.886 | 0.794 | 0.616 | 0.023 | 0.566 | 0.576 | 0.618 | 0.652 | 0.659 | 0.339 |
| L LimbicA TempPole 1 | 0.868 | 0.01 | 0.844 | 0.848 | 0.869 | 0.883 | 0.886 | 0.727 | 0.616 | 0.023 | 0.566 | 0.576 | 0.618 | 0.652 | 0.659 | 0.433 |
| L LimbicA TempPole 2 | 0.815 | 0.015 | 0.781 | 0.788 | 0.816 | 0.836 | 0.839 | 0.764 | 0.563 | 0.024 | 0.512 | 0.522 | 0.565 | 0.601 | 0.608 | 0.495 |
| L LimbicA TempPole 3 | 0.735 | 0.021 | 0.689 | 0.697 | 0.737 | 0.767 | 0.771 | 0.614 | 0.751 | 0.016 | 0.717 | 0.723 | 0.752 | 0.777 | 0.78 | 0.239 |
| L LimbicA TempPole 4 | 0.748 | 0.02 | 0.703 | 0.711 | 0.749 | 0.777 | 0.783 | 0.75 | 0.669 | 0.021 | 0.624 | 0.633 | 0.67 | 0.701 | 0.707 | 0.156 |
| L ContA Temp 1 | 0.727 | 0.021 | 0.679 | 0.69 | 0.728 | 0.758 | 0.763 | 0.464 | 0.631 | 0.022 | 0.585 | 0.592 | 0.632 | 0.664 | 0.671 | 0.583 |
| L ContA IPS 1 | 0.737 | 0.021 | 0.691 | 0.7 | 0.738 | 0.768 | 0.772 | 0.745 | 0.598 | 0.023 | 0.549 | 0.557 | 0.599 | 0.633 | 0.639 | 0.617 |
| L ContA IPS 2 | 0.733 | 0.021 | 0.687 | 0.696 | 0.734 | 0.766 | 0.77 | 0.623 | 0.594 | 0.023 | 0.545 | 0.552 | 0.596 | 0.629 | 0.635 | 0.547 |
| L ContA IPS 3 | 0.708 | 0.023 | 0.66 | 0.668 | 0.71 | 0.743 | 0.747 | 0.525 | 0.605 | 0.023 | 0.556 | 0.564 | 0.606 | 0.639 | 0.646 | 0.68 |
| L ContA PFCd 1 | 0.71 | 0.023 | 0.66 | 0.67 | 0.712 | 0.745 | 0.75 | 0.579 | 0.576 | 0.024 | 0.527 | 0.535 | 0.577 | 0.614 | 0.619 | 0.423 |
| L ContA PFClv 1 | 0.706 | 0.023 | 0.655 | 0.665 | 0.708 | 0.74 | 0.745 | 0.708 | 0.602 | 0.023 | 0.553 | 0.562 | 0.603 | 0.637 | 0.643 | 0.589 |
| L ContA PFCl 1 | 0.736 | 0.021 | 0.689 | 0.697 | 0.737 | 0.768 | 0.772 | 0.772 | 0.585 | 0.024 | 0.535 | 0.543 | 0.586 | 0.623 | 0.629 | 0.534 |
| L ContA PFCl 2 | 0.702 | 0.023 | 0.649 | 0.66 | 0.704 | 0.737 | 0.742 | 0.736 | 0.659 | 0.02 | 0.614 | 0.623 | 0.66 | 0.69 | 0.695 | 0.532 |
| L ContA PFCl 3 | 0.707 | 0.023 | 0.654 | 0.667 | 0.709 | 0.742 | 0.747 | 0.768 | 0.634 | 0.022 | 0.586 | 0.595 | 0.635 | 0.669 | 0.675 | 0.708 |
| L ContA Cingm 1 | 0.757 | 0.019 | 0.714 | 0.723 | 0.759 | 0.786 | 0.789 | 0.611 | 0.573 | 0.023 | 0.522 | 0.531 | 0.574 | 0.608 | 0.614 | 0.31 |
| L ContB Temp 1 | 0.764 | 0.019 | 0.723 | 0.731 | 0.766 | 0.791 | 0.796 | 0.584 | 0.57 | 0.024 | 0.519 | 0.527 | 0.572 | 0.606 | 0.613 | 0.373 |
| L ContB IPL 1 | 0.752 | 0.019 | 0.709 | 0.717 | 0.753 | 0.781 | 0.785 | 0.736 | 0.572 | 0.024 | 0.521 | 0.528 | 0.574 | 0.609 | 0.614 | 0.566 |
| L ContB PFCl 1 | 0.706 | 0.023 | 0.655 | 0.665 | 0.708 | 0.741 | 0.747 | 0.743 | 0.548 | 0.023 | 0.499 | 0.508 | 0.549 | 0.584 | 0.589 | 0.444 |
| L ContB PFClv 1 | 0.747 | 0.02 | 0.703 | 0.71 | 0.749 | 0.777 | 0.782 | 0.611 | 0.576 | 0.024 | 0.524 | 0.534 | 0.578 | 0.614 | 0.619 | 0.393 |
| L ContB PFClv 2 | 0.739 | 0.021 | 0.694 | 0.702 | 0.741 | 0.77 | 0.774 | 0.717 | 0.56 | 0.024 | 0.508 | 0.518 | 0.562 | 0.598 | 0.604 | 0.429 |
| L ContC pCun 1 | 0.713 | 0.022 | 0.663 | 0.674 | 0.715 | 0.747 | 0.753 | 0.746 | 0.603 | 0.022 | 0.556 | 0.565 | 0.604 | 0.638 | 0.644 | 0.485 |
| L ContC pCun 2 | 0.755 | 0.019 | 0.714 | 0.721 | 0.757 | 0.785 | 0.788 | 0.763 | 0.532 | 0.023 | 0.481 | 0.49 | 0.533 | 0.567 | 0.573 | 0.475 |
| L ContC Cingp 1 | 0.698 | 0.024 | 0.646 | 0.656 | 0.7 | 0.733 | 0.739 | 0.702 | 0.571 | 0.024 | 0.519 | 0.528 | 0.572 | 0.608 | 0.613 | 0.331 |
| L DefaultA IPL 1 | 0.727 | 0.022 | 0.679 | 0.69 | 0.728 | 0.76 | 0.766 | 0.649 | 0.59 | 0.024 | 0.54 | 0.548 | 0.592 | 0.627 | 0.632 | 0.554 |
| L DefaultA PFCd 1 | 0.761 | 0.019 | 0.72 | 0.727 | 0.762 | 0.789 | 0.793 | 0.788 | 0.544 | 0.024 | 0.492 | 0.503 | 0.545 | 0.581 | 0.588 | 0.416 |
| L DefaultA pCunPCC 1 | 0.704 | 0.023 | 0.654 | 0.663 | 0.706 | 0.739 | 0.745 | 0.703 | 0.585 | 0.024 | 0.536 | 0.543 | 0.586 | 0.621 | 0.628 | 0.405 |
| L DefaultA pCunPCC 2 | 0.708 | 0.023 | 0.657 | 0.669 | 0.71 | 0.743 | 0.749 | 0.704 | 0.591 | 0.023 | 0.543 | 0.55 | 0.593 | 0.626 | 0.633 | 0.309 |
| L DefaultA pCunPCC 3 | 0.696 | 0.024 | 0.643 | 0.653 | 0.698 | 0.732 | 0.738 | 0.706 | 0.549 | 0.023 | 0.499 | 0.509 | 0.549 | 0.585 | 0.592 | 0.342 |
| L DefaultA PFCm 1 | 0.758 | 0.019 | 0.715 | 0.724 | 0.76 | 0.786 | 0.791 | 0.707 | 0.585 | 0.024 | 0.537 | 0.544 | 0.587 | 0.622 | 0.63 | 0.292 |
| L DefaultA PFCm 2 | 0.761 | 0.019 | 0.717 | 0.726 | 0.763 | 0.79 | 0.793 | 0.597 | 0.594 | 0.023 | 0.545 | 0.552 | 0.595 | 0.63 | 0.637 | 0.481 |
| L DefaultA PFCm 3 | 0.708 | 0.023 | 0.656 | 0.666 | 0.709 | 0.743 | 0.747 | 0.772 | 0.545 | 0.023 | 0.495 | 0.504 | 0.546 | 0.58 | 0.586 | 0.374 |
| L DefaultB Temp 1 | 0.789 | 0.016 | 0.752 | 0.759 | 0.79 | 0.813 | 0.816 | 0.752 | 0.535 | 0.023 | 0.483 | 0.492 | 0.536 | 0.572 | 0.578 | 0.295 |
| L DefaultB Temp 2 | 0.806 | 0.016 | 0.771 | 0.777 | 0.807 | 0.829 | 0.833 | 0.526 | 0.602 | 0.026 | 0.548 | 0.558 | 0.604 | 0.642 | 0.648 | 0.545 |
| L DefaultB Temp 3 | 0.722 | 0.022 | 0.672 | 0.684 | 0.723 | 0.755 | 0.759 | 0.511 | 0.537 | 0.024 | 0.485 | 0.495 | 0.539 | 0.574 | 0.578 | 0.236 |
| L DefaultB Temp 4 | 0.709 | 0.023 | 0.657 | 0.668 | 0.71 | 0.744 | 0.75 | 0.594 | 0.541 | 0.023 | 0.49 | 0.5 | 0.542 | 0.578 | 0.583 | 0.28 |
| L DefaultB IPL 1 | 0.728 | 0.021 | 0.68 | 0.69 | 0.729 | 0.759 | 0.765 | 0.652 | 0.583 | 0.023 | 0.534 | 0.543 | 0.585 | 0.619 | 0.626 | 0.463 |
| L DefaultB PFCd 1 | 0.71 | 0.023 | 0.661 | 0.671 | 0.712 | 0.744 | 0.749 | 0.769 | 0.557 | 0.023 | 0.507 | 0.516 | 0.558 | 0.592 | 0.601 | 0.537 |
| L DefaultB PFCd 2 | 0.78 | 0.017 | 0.741 | 0.749 | 0.781 | 0.805 | 0.809 | 0.818 | 0.572 | 0.024 | 0.521 | 0.531 | 0.573 | 0.608 | 0.614 | 0.514 |
| L DefaultB PFCd 3 | 0.72 | 0.022 | 0.672 | 0.681 | 0.722 | 0.752 | 0.758 | 0.869 | 0.614 | 0.023 | 0.567 | 0.576 | 0.615 | 0.649 | 0.654 | 0.518 |
| L DefaultB PFCd 4 | 0.738 | 0.02 | 0.692 | 0.701 | 0.74 | 0.769 | 0.773 | 0.741 | 0.578 | 0.023 | 0.527 | 0.536 | 0.579 | 0.614 | 0.619 | 0.376 |
| L DefaultB PFCv 1 | 0.706 | 0.023 | 0.655 | 0.665 | 0.707 | 0.741 | 0.746 | 0.72 | 0.544 | 0.024 | 0.488 | 0.501 | 0.546 | 0.58 | 0.587 | 0.457 |
| L DefaultB PFCv 2 | 0.751 | 0.02 | 0.706 | 0.716 | 0.752 | 0.779 | 0.784 | 0.674 | 0.571 | 0.023 | 0.521 | 0.532 | 0.572 | 0.606 | 0.613 | 0.516 |
| L DefaultB PFCv 3 | 0.714 | 0.022 | 0.667 | 0.675 | 0.715 | 0.748 | 0.752 | 0.816 | 0.569 | 0.024 | 0.519 | 0.527 | 0.57 | 0.605 | 0.61 | 0.487 |
| L DefaultB PFCv 4 | 0.704 | 0.023 | 0.652 | 0.661 | 0.705 | 0.739 | 0.745 | 0.819 | 0.562 | 0.024 | 0.508 | 0.519 | 0.564 | 0.6 | 0.605 | 0.505 |
| L DefaultC IPL 1 | 0.741 | 0.02 | 0.695 | 0.704 | 0.743 | 0.772 | 0.777 | 0.545 | 0.593 | 0.023 | 0.543 | 0.552 | 0.595 | 0.628 | 0.633 | 0.435 |
| L DefaultC Rsp 1 | 0.7 | 0.023 | 0.648 | 0.659 | 0.701 | 0.735 | 0.742 | 0.749 | 0.624 | 0.022 | 0.577 | 0.585 | 0.625 | 0.656 | 0.662 | 0.402 |
| L DefaultC PHC 1 | 0.706 | 0.023 | 0.653 | 0.664 | 0.707 | 0.74 | 0.745 | 0.741 | 0.565 | 0.024 | 0.513 | 0.523 | 0.566 | 0.602 | 0.607 | 0.172 |
| L TempPar 1 | 0.726 | 0.022 | 0.677 | 0.686 | 0.728 | 0.759 | 0.765 | 0.574 | 0.591 | 0.023 | 0.544 | 0.552 | 0.592 | 0.627 | 0.633 | 0.534 |
| L TempPar 2 | 0.735 | 0.021 | 0.689 | 0.697 | 0.736 | 0.767 | 0.771 | 0.562 | 0.665 | 0.02 | 0.622 | 0.63 | 0.667 | 0.697 | 0.702 | 0.623 |
| R VisCent ExStr 1 | 0.713 | 0.022 | 0.666 | 0.673 | 0.715 | 0.747 | 0.752 | 0.605 | 0.692 | 0.019 | 0.653 | 0.66 | 0.693 | 0.722 | 0.727 | 0.597 |
| R VisCent ExStr 2 | 0.742 | 0.021 | 0.697 | 0.706 | 0.744 | 0.773 | 0.778 | 0.434 | 0.678 | 0.02 | 0.635 | 0.643 | 0.68 | 0.709 | 0.714 | 0.569 |
| R VisCent Striate 1 | 0.731 | 0.021 | 0.683 | 0.691 | 0.732 | 0.763 | 0.766 | 0.549 | 0.891 | 0.006 | 0.878 | 0.88 | 0.891 | 0.901 | 0.902 | 0.834 |
| R VisCent ExStr 3 | 0.797 | 0.016 | 0.76 | 0.769 | 0.798 | 0.821 | 0.826 | 0.489 | 0.789 | 0.013 | 0.761 | 0.766 | 0.79 | 0.809 | 0.812 | 0.803 |
| R VisCent ExStr 4 | 0.741 | 0.021 | 0.693 | 0.703 | 0.742 | 0.771 | 0.776 | 0.278 | 0.673 | 0.021 | 0.627 | 0.636 | 0.674 | 0.706 | 0.71 | 0.71 |
| R VisCent ExStr 5 | 0.774 | 0.018 | 0.735 | 0.742 | 0.776 | 0.8 | 0.805 | 0.566 | 0.753 | 0.016 | 0.719 | 0.724 | 0.754 | 0.777 | 0.781 | 0.821 |
| R VisPeri ExStrInf 1 | 0.691 | 0.024 | 0.637 | 0.65 | 0.693 | 0.727 | 0.733 | 0.586 | 0.621 | 0.022 | 0.573 | 0.583 | 0.623 | 0.655 | 0.662 | 0.418 |
| R VisPeri ExStrInf 2 | 0.701 | 0.023 | 0.648 | 0.66 | 0.703 | 0.737 | 0.742 | 0.665 | 0.667 | 0.02 | 0.624 | 0.632 | 0.669 | 0.698 | 0.704 | 0.549 |
| R VisPeri StriCal 1 | 0.706 | 0.023 | 0.656 | 0.664 | 0.707 | 0.741 | 0.745 | 0.587 | 0.816 | 0.011 | 0.793 | 0.797 | 0.817 | 0.834 | 0.836 | 0.713 |
| R VisPeri ExStrSup 1 | 0.739 | 0.02 | 0.693 | 0.704 | 0.74 | 0.769 | 0.775 | 0.667 | 0.74 | 0.016 | 0.706 | 0.712 | 0.741 | 0.765 | 0.769 | 0.715 |
| R VisPeri ExStrSup 2 | 0.758 | 0.019 | 0.716 | 0.724 | 0.759 | 0.786 | 0.791 | 0.617 | 0.663 | 0.02 | 0.621 | 0.628 | 0.663 | 0.695 | 0.7 | 0.692 |
| R VisPeri ExStrSup 3 | 0.774 | 0.018 | 0.735 | 0.742 | 0.776 | 0.801 | 0.805 | 0.606 | 0.687 | 0.02 | 0.646 | 0.652 | 0.688 | 0.717 | 0.723 | 0.719 |
| R SomMotA 1 | 0.783 | 0.017 | 0.744 | 0.753 | 0.784 | 0.807 | 0.812 | 0.648 | 0.62 | 0.023 | 0.57 | 0.58 | 0.621 | 0.654 | 0.66 | 0.445 |
| R SomMotA 2 | 0.717 | 0.022 | 0.667 | 0.677 | 0.718 | 0.75 | 0.755 | 0.679 | 0.63 | 0.022 | 0.584 | 0.591 | 0.631 | 0.663 | 0.669 | 0.551 |
| R SomMotA 3 | 0.71 | 0.023 | 0.66 | 0.669 | 0.712 | 0.744 | 0.751 | 0.756 | 0.624 | 0.023 | 0.577 | 0.584 | 0.626 | 0.659 | 0.666 | 0.629 |
| R SomMotA 4 | 0.753 | 0.019 | 0.711 | 0.719 | 0.755 | 0.782 | 0.786 | 0.602 | 0.754 | 0.016 | 0.72 | 0.725 | 0.755 | 0.778 | 0.781 | 0.557 |
| R SomMotA 5 | 0.779 | 0.017 | 0.74 | 0.749 | 0.781 | 0.805 | 0.809 | 0.659 | 0.599 | 0.023 | 0.549 | 0.559 | 0.6 | 0.635 | 0.641 | 0.474 |
| R SomMotA 6 | 0.728 | 0.021 | 0.68 | 0.691 | 0.73 | 0.761 | 0.766 | 0.591 | 0.698 | 0.019 | 0.657 | 0.664 | 0.699 | 0.726 | 0.73 | 0.646 |
| R SomMotA 7 | 0.733 | 0.021 | 0.688 | 0.696 | 0.735 | 0.765 | 0.77 | 0.604 | 0.582 | 0.023 | 0.533 | 0.541 | 0.584 | 0.617 | 0.623 | 0.528 |
| R SomMotA 8 | 0.712 | 0.023 | 0.662 | 0.673 | 0.714 | 0.747 | 0.751 | 0.732 | 0.566 | 0.023 | 0.516 | 0.525 | 0.567 | 0.601 | 0.606 | 0.486 |
| R SomMotA 9 | 0.747 | 0.02 | 0.702 | 0.711 | 0.748 | 0.777 | 0.781 | 0.652 | 0.544 | 0.024 | 0.493 | 0.503 | 0.545 | 0.581 | 0.588 | 0.418 |
| R SomMotA 10 | 0.729 | 0.021 | 0.681 | 0.691 | 0.73 | 0.761 | 0.765 | 0.652 | 0.57 | 0.023 | 0.52 | 0.531 | 0.572 | 0.607 | 0.612 | 0.489 |
| R SomMotA 11 | 0.735 | 0.021 | 0.688 | 0.7 | 0.737 | 0.766 | 0.771 | 0.562 | 0.61 | 0.023 | 0.563 | 0.569 | 0.611 | 0.645 | 0.651 | 0.55 |
| R SomMotB Aud 1 | 0.712 | 0.022 | 0.663 | 0.671 | 0.713 | 0.746 | 0.752 | 0.66 | 0.574 | 0.024 | 0.523 | 0.532 | 0.576 | 0.61 | 0.616 | 0.39 |
| R SomMotB Aud 2 | 0.734 | 0.021 | 0.688 | 0.697 | 0.736 | 0.765 | 0.771 | 0.654 | 0.572 | 0.024 | 0.52 | 0.529 | 0.573 | 0.611 | 0.616 | 0.365 |
| R SomMotB S2 1 | 0.699 | 0.023 | 0.647 | 0.658 | 0.7 | 0.734 | 0.739 | 0.701 | 0.541 | 0.024 | 0.488 | 0.499 | 0.542 | 0.578 | 0.583 | 0.385 |
| R SomMotB S2 2 | 0.711 | 0.023 | 0.66 | 0.669 | 0.712 | 0.745 | 0.75 | 0.696 | 0.552 | 0.025 | 0.502 | 0.509 | 0.553 | 0.591 | 0.599 | 0.278 |
| R SomMotB S2 3 | 0.736 | 0.021 | 0.689 | 0.698 | 0.738 | 0.768 | 0.772 | 0.781 | 0.628 | 0.022 | 0.584 | 0.59 | 0.629 | 0.661 | 0.667 | 0.421 |
| R SomMotB S2 4 | 0.739 | 0.02 | 0.695 | 0.702 | 0.741 | 0.77 | 0.776 | 0.819 | 0.645 | 0.022 | 0.598 | 0.607 | 0.646 | 0.678 | 0.683 | 0.468 |
| R SomMotB Cent 1 | 0.707 | 0.023 | 0.657 | 0.664 | 0.709 | 0.741 | 0.748 | 0.689 | 0.609 | 0.023 | 0.561 | 0.569 | 0.61 | 0.643 | 0.649 | 0.413 |
| R DorsAttnA TempOcc 1 | 0.714 | 0.022 | 0.663 | 0.672 | 0.715 | 0.748 | 0.753 | 0.597 | 0.628 | 0.023 | 0.578 | 0.588 | 0.63 | 0.664 | 0.67 | 0.562 |
| R DorsAttnA ParOcc 1 | 0.715 | 0.022 | 0.667 | 0.675 | 0.717 | 0.747 | 0.753 | 0.512 | 0.607 | 0.023 | 0.556 | 0.566 | 0.608 | 0.644 | 0.649 | 0.587 |
| R DorsAttnA SPL 1 | 0.713 | 0.022 | 0.664 | 0.673 | 0.715 | 0.748 | 0.752 | 0.495 | 0.619 | 0.023 | 0.569 | 0.58 | 0.62 | 0.654 | 0.66 | 0.648 |
| R DorsAttnA SPL 2 | 0.741 | 0.021 | 0.697 | 0.705 | 0.743 | 0.773 | 0.777 | 0.638 | 0.6 | 0.024 | 0.55 | 0.559 | 0.602 | 0.636 | 0.643 | 0.467 |
| R DorsAttnA SPL 3 | 0.735 | 0.021 | 0.69 | 0.698 | 0.736 | 0.766 | 0.772 | 0.666 | 0.596 | 0.023 | 0.545 | 0.554 | 0.597 | 0.631 | 0.637 | 0.598 |
| R DorsAttnA SPL 4 | 0.725 | 0.022 | 0.676 | 0.686 | 0.727 | 0.758 | 0.763 | 0.486 | 0.582 | 0.023 | 0.532 | 0.542 | 0.583 | 0.617 | 0.624 | 0.629 |
| R DorsAttnB PostC 1 | 0.721 | 0.022 | 0.672 | 0.682 | 0.722 | 0.754 | 0.76 | 0.64 | 0.598 | 0.023 | 0.551 | 0.558 | 0.599 | 0.634 | 0.641 | 0.341 |
| R DorsAttnB PostC 2 | 0.723 | 0.022 | 0.675 | 0.683 | 0.724 | 0.755 | 0.76 | 0.633 | 0.587 | 0.023 | 0.537 | 0.544 | 0.589 | 0.622 | 0.629 | 0.542 |
| R DorsAttnB PostC 3 | 0.747 | 0.02 | 0.703 | 0.712 | 0.748 | 0.776 | 0.782 | 0.75 | 0.551 | 0.024 | 0.501 | 0.508 | 0.553 | 0.588 | 0.594 | 0.384 |
| R DorsAttnB PostC 4 | 0.781 | 0.017 | 0.744 | 0.75 | 0.783 | 0.807 | 0.811 | 0.68 | 0.556 | 0.023 | 0.503 | 0.515 | 0.557 | 0.59 | 0.596 | 0.314 |
| R DorsAttnB FEF 1 | 0.718 | 0.022 | 0.668 | 0.679 | 0.719 | 0.75 | 0.756 | 0.685 | 0.554 | 0.023 | 0.503 | 0.513 | 0.555 | 0.589 | 0.596 | 0.379 |
| R SalVentAttnA ParOper 1 | 0.727 | 0.021 | 0.68 | 0.689 | 0.728 | 0.76 | 0.765 | 0.765 | 0.569 | 0.024 | 0.519 | 0.529 | 0.57 | 0.607 | 0.613 | 0.236 |
| R SalVentAttnA PrC 1 | 0.717 | 0.022 | 0.668 | 0.677 | 0.718 | 0.75 | 0.756 | 0.707 | 0.693 | 0.019 | 0.653 | 0.66 | 0.695 | 0.723 | 0.728 | 0.652 |
| R SalVentAttnA Ins 1 | 0.733 | 0.021 | 0.688 | 0.696 | 0.735 | 0.765 | 0.77 | 0.667 | 0.582 | 0.025 | 0.53 | 0.538 | 0.584 | 0.62 | 0.626 | 0.029 |
| R SalVentAttnA Ins 2 | 0.711 | 0.023 | 0.661 | 0.672 | 0.713 | 0.746 | 0.75 | 0.761 | 0.551 | 0.024 | 0.501 | 0.51 | 0.553 | 0.588 | 0.593 | 0.392 |
| R SalVentAttnA FrOper 1 | 0.691 | 0.024 | 0.638 | 0.649 | 0.693 | 0.728 | 0.734 | 0.747 | 0.548 | 0.024 | 0.497 | 0.507 | 0.549 | 0.585 | 0.59 | 0.421 |
| R SalVentAttnA FrMed 1 | 0.703 | 0.023 | 0.653 | 0.662 | 0.704 | 0.738 | 0.743 | 0.74 | 0.575 | 0.024 | 0.525 | 0.533 | 0.576 | 0.611 | 0.618 | 0.453 |
| R SalVentAttnA ParMed 1 | 0.712 | 0.022 | 0.664 | 0.672 | 0.714 | 0.746 | 0.752 | 0.688 | 0.533 | 0.023 | 0.482 | 0.492 | 0.534 | 0.568 | 0.573 | 0.418 |
| R SalVentAttnA ParMed 2 | 0.699 | 0.023 | 0.649 | 0.658 | 0.701 | 0.735 | 0.739 | 0.636 | 0.532 | 0.023 | 0.481 | 0.492 | 0.534 | 0.567 | 0.572 | 0.372 |
| R SalVentAttnA FrMed 2 | 0.729 | 0.021 | 0.682 | 0.69 | 0.73 | 0.76 | 0.766 | 0.805 | 0.614 | 0.023 | 0.565 | 0.573 | 0.615 | 0.649 | 0.657 | 0.424 |
| R SalVentAttnB IPL 1 | 0.758 | 0.019 | 0.715 | 0.724 | 0.759 | 0.786 | 0.79 | 0.756 | 0.594 | 0.023 | 0.545 | 0.552 | 0.595 | 0.629 | 0.635 | 0.574 |
| R SalVentAttnB PFClv 1 | 0.71 | 0.023 | 0.658 | 0.67 | 0.712 | 0.744 | 0.749 | 0.656 | 0.605 | 0.023 | 0.555 | 0.565 | 0.605 | 0.64 | 0.645 | 0.621 |
| R SalVentAttnB PFCl 1 | 0.739 | 0.02 | 0.693 | 0.702 | 0.741 | 0.77 | 0.775 | 0.754 | 0.529 | 0.022 | 0.48 | 0.488 | 0.531 | 0.563 | 0.568 | 0.42 |
| R SalVentAttnB Ins 1 | 0.705 | 0.023 | 0.654 | 0.664 | 0.707 | 0.741 | 0.746 | 0.735 | 0.567 | 0.023 | 0.516 | 0.526 | 0.568 | 0.603 | 0.61 | 0.495 |
| R SalVentAttnB Ins 2 | 0.702 | 0.023 | 0.649 | 0.659 | 0.703 | 0.737 | 0.742 | 0.718 | 0.65 | 0.022 | 0.601 | 0.611 | 0.652 | 0.683 | 0.69 | 0.598 |
| R SalVentAttnB PFCmp 1 | 0.707 | 0.023 | 0.658 | 0.666 | 0.709 | 0.742 | 0.747 | 0.759 | 0.578 | 0.023 | 0.526 | 0.538 | 0.579 | 0.615 | 0.621 | 0.491 |
| R LimbicB OFC 2 | 0.731 | 0.021 | 0.684 | 0.692 | 0.732 | 0.762 | 0.767 | 0.776 | 0.56 | 0.023 | 0.511 | 0.522 | 0.562 | 0.596 | 0.602 | 0.375 |
| R LimbicB OFC 3 | 0.719 | 0.022 | 0.67 | 0.68 | 0.721 | 0.751 | 0.758 | 0.807 | 0.56 | 0.024 | 0.508 | 0.516 | 0.561 | 0.596 | 0.604 | 0.234 |
| R LimbicB OFC 4 | 0.764 | 0.019 | 0.721 | 0.73 | 0.765 | 0.791 | 0.796 | 0.588 | 0.597 | 0.023 | 0.548 | 0.556 | 0.598 | 0.632 | 0.639 | 0.485 |
| R LimbicA TempPole 1 | 0.725 | 0.021 | 0.678 | 0.688 | 0.727 | 0.757 | 0.763 | 0.691 | 0.591 | 0.024 | 0.539 | 0.549 | 0.592 | 0.627 | 0.635 | 0 |
| R LimbicA TempPole 2 | 0.808 | 0.015 | 0.773 | 0.78 | 0.809 | 0.83 | 0.834 | 0.793 | 0.539 | 0.023 | 0.488 | 0.497 | 0.541 | 0.574 | 0.581 | 0.358 |
| R LimbicA TempPole 3 | 0.737 | 0.021 | 0.691 | 0.698 | 0.738 | 0.767 | 0.773 | 0.696 | 0.697 | 0.02 | 0.654 | 0.663 | 0.698 | 0.728 | 0.734 | 0.222 |
| R LimbicA TempPole 4 | 0.754 | 0.019 | 0.712 | 0.719 | 0.755 | 0.783 | 0.788 | 0.735 | 0.686 | 0.02 | 0.644 | 0.651 | 0.687 | 0.717 | 0.721 | 0.615 |
| R ContA IPS 1 | 0.752 | 0.019 | 0.709 | 0.717 | 0.753 | 0.78 | 0.786 | 0.716 | 0.619 | 0.023 | 0.57 | 0.579 | 0.621 | 0.654 | 0.66 | 0.628 |
| R ContA IPS 2 | 0.762 | 0.019 | 0.721 | 0.729 | 0.764 | 0.79 | 0.795 | 0.735 | 0.572 | 0.024 | 0.52 | 0.531 | 0.574 | 0.61 | 0.616 | 0.472 |
| R ContA PFCd 1 | 0.727 | 0.022 | 0.68 | 0.689 | 0.728 | 0.759 | 0.763 | 0.721 | 0.575 | 0.023 | 0.523 | 0.533 | 0.576 | 0.611 | 0.616 | 0.521 |
| R ContA PFCl 1 | 0.709 | 0.023 | 0.657 | 0.669 | 0.71 | 0.744 | 0.749 | 0.742 | 0.649 | 0.021 | 0.604 | 0.612 | 0.65 | 0.681 | 0.687 | 0.659 |
| R ContA PFCl 2 | 0.706 | 0.023 | 0.655 | 0.665 | 0.708 | 0.74 | 0.747 | 0.676 | 0.59 | 0.023 | 0.54 | 0.551 | 0.591 | 0.625 | 0.631 | 0.519 |
| R ContA Cingm 1 | 0.737 | 0.02 | 0.692 | 0.701 | 0.738 | 0.768 | 0.772 | 0.62 | 0.531 | 0.023 | 0.48 | 0.491 | 0.532 | 0.566 | 0.569 | 0.383 |
| R ContB Temp 1 | 0.746 | 0.02 | 0.701 | 0.711 | 0.747 | 0.776 | 0.781 | 0.641 | 0.587 | 0.024 | 0.535 | 0.545 | 0.588 | 0.624 | 0.629 | 0.385 |
| R ContB Temp 2 | 0.721 | 0.022 | 0.673 | 0.681 | 0.722 | 0.755 | 0.758 | 0.506 | 0.561 | 0.024 | 0.51 | 0.519 | 0.563 | 0.598 | 0.604 | 0.361 |
| R ContB IPL 1 | 0.747 | 0.02 | 0.703 | 0.713 | 0.749 | 0.776 | 0.782 | 0.719 | 0.584 | 0.023 | 0.534 | 0.544 | 0.585 | 0.619 | 0.625 | 0.521 |
| R ContB IPL 2 | 0.758 | 0.019 | 0.714 | 0.724 | 0.759 | 0.786 | 0.79 | 0.792 | 0.597 | 0.022 | 0.55 | 0.557 | 0.599 | 0.631 | 0.637 | 0.495 |
| R ContB PFCld 1 | 0.745 | 0.02 | 0.701 | 0.709 | 0.746 | 0.775 | 0.78 | 0.759 | 0.601 | 0.023 | 0.554 | 0.563 | 0.602 | 0.636 | 0.644 | 0.602 |
| R ContB PFCld 2 | 0.711 | 0.023 | 0.661 | 0.671 | 0.713 | 0.745 | 0.75 | 0.737 | 0.578 | 0.024 | 0.528 | 0.536 | 0.58 | 0.616 | 0.621 | 0.484 |
| R ContB PFClv 1 | 0.733 | 0.021 | 0.689 | 0.695 | 0.734 | 0.765 | 0.77 | 0.661 | 0.598 | 0.023 | 0.551 | 0.558 | 0.599 | 0.634 | 0.64 | 0.545 |
| R ContB PFClv 2 | 0.727 | 0.022 | 0.679 | 0.688 | 0.728 | 0.759 | 0.765 | 0.539 | 0.612 | 0.023 | 0.565 | 0.572 | 0.612 | 0.646 | 0.652 | 0.561 |
| R ContB PFCmp 1 | 0.72 | 0.022 | 0.671 | 0.68 | 0.722 | 0.753 | 0.758 | 0.833 | 0.582 | 0.023 | 0.531 | 0.54 | 0.584 | 0.617 | 0.623 | 0.581 |
| R ContB PFCld 3 | 0.709 | 0.023 | 0.656 | 0.668 | 0.711 | 0.743 | 0.748 | 0.681 | 0.558 | 0.024 | 0.507 | 0.516 | 0.559 | 0.594 | 0.599 | 0.474 |
| R ContC pCun 1 | 0.712 | 0.022 | 0.661 | 0.671 | 0.713 | 0.746 | 0.752 | 0.621 | 0.611 | 0.022 | 0.565 | 0.572 | 0.612 | 0.647 | 0.652 | 0.523 |
| R ContC pCun 2 | 0.711 | 0.022 | 0.663 | 0.671 | 0.712 | 0.745 | 0.751 | 0.683 | 0.56 | 0.024 | 0.509 | 0.518 | 0.561 | 0.598 | 0.603 | 0.297 |
| R ContC Cingp 1 | 0.712 | 0.023 | 0.662 | 0.672 | 0.714 | 0.746 | 0.751 | 0.679 | 0.549 | 0.024 | 0.498 | 0.508 | 0.551 | 0.587 | 0.593 | 0.196 |
| R DefaultA IPL 1 | 0.732 | 0.021 | 0.683 | 0.693 | 0.734 | 0.764 | 0.769 | 0.732 | 0.576 | 0.024 | 0.525 | 0.535 | 0.577 | 0.612 | 0.619 | 0.394 |
| R DefaultA PFCd 1 | 0.717 | 0.022 | 0.666 | 0.677 | 0.719 | 0.75 | 0.757 | 0.73 | 0.543 | 0.023 | 0.494 | 0.502 | 0.544 | 0.579 | 0.585 | 0.549 |
| R DefaultA pCunPCC 1 | 0.696 | 0.024 | 0.644 | 0.654 | 0.698 | 0.732 | 0.738 | 0.72 | 0.56 | 0.024 | 0.508 | 0.519 | 0.561 | 0.597 | 0.604 | 0.365 |
| R DefaultA PFCm 1 | 0.704 | 0.023 | 0.652 | 0.663 | 0.706 | 0.74 | 0.746 | 0.72 | 0.531 | 0.023 | 0.481 | 0.489 | 0.532 | 0.567 | 0.573 | 0.35 |
| R DefaultA PFCm 2 | 0.73 | 0.021 | 0.682 | 0.691 | 0.731 | 0.763 | 0.768 | 0.604 | 0.548 | 0.024 | 0.495 | 0.505 | 0.549 | 0.585 | 0.593 | 0.415 |
| R DefaultA PFCm 3 | 0.73 | 0.021 | 0.682 | 0.692 | 0.732 | 0.761 | 0.766 | 0.794 | 0.531 | 0.022 | 0.48 | 0.49 | 0.532 | 0.565 | 0.571 | 0.44 |
| R DefaultB Temp 1 | 0.757 | 0.019 | 0.714 | 0.722 | 0.759 | 0.787 | 0.79 | 0.672 | 0.576 | 0.023 | 0.524 | 0.534 | 0.577 | 0.611 | 0.617 | 0.363 |
| R DefaultB AntTemp 1 | 0.748 | 0.02 | 0.702 | 0.712 | 0.75 | 0.778 | 0.783 | 0.682 | 0.537 | 0.024 | 0.483 | 0.495 | 0.538 | 0.572 | 0.578 | 0.215 |
| R DefaultB PFCd 1 | 0.751 | 0.019 | 0.709 | 0.717 | 0.753 | 0.78 | 0.785 | 0.764 | 0.548 | 0.023 | 0.498 | 0.508 | 0.549 | 0.583 | 0.59 | 0.498 |
| R DefaultB PFCv 1 | 0.693 | 0.024 | 0.641 | 0.651 | 0.695 | 0.729 | 0.734 | 0.713 | 0.586 | 0.024 | 0.536 | 0.544 | 0.588 | 0.622 | 0.628 | 0.416 |
| R DefaultC IPL 1 | 0.727 | 0.021 | 0.678 | 0.689 | 0.729 | 0.759 | 0.764 | 0.507 | 0.592 | 0.023 | 0.544 | 0.552 | 0.593 | 0.628 | 0.634 | 0.514 |
| R DefaultC Rsp 1 | 0.697 | 0.023 | 0.646 | 0.656 | 0.698 | 0.733 | 0.739 | 0.774 | 0.629 | 0.022 | 0.582 | 0.591 | 0.63 | 0.664 | 0.67 | 0.504 |
| R DefaultC PHC 1 | 0.715 | 0.022 | 0.667 | 0.676 | 0.716 | 0.748 | 0.754 | 0.672 | 0.543 | 0.023 | 0.493 | 0.501 | 0.545 | 0.579 | 0.585 | 0.273 |
| R TempPar 1 | 0.73 | 0.021 | 0.683 | 0.693 | 0.731 | 0.762 | 0.768 | 0.62 | 0.566 | 0.023 | 0.517 | 0.523 | 0.567 | 0.602 | 0.608 | 0.424 |
| R TempPar 2 | 0.717 | 0.022 | 0.667 | 0.678 | 0.719 | 0.75 | 0.755 | 0.67 | 0.625 | 0.023 | 0.576 | 0.588 | 0.627 | 0.661 | 0.667 | 0.607 |
| R TempPar 3 | 0.753 | 0.019 | 0.709 | 0.718 | 0.754 | 0.782 | 0.786 | 0.64 | 0.674 | 0.021 | 0.63 | 0.638 | 0.675 | 0.707 | 0.713 | 0.654 |
| R TempPar 4 | 0.74 | 0.02 | 0.694 | 0.703 | 0.741 | 0.771 | 0.776 | 0.715 | 0.594 | 0.024 | 0.544 | 0.553 | 0.596 | 0.631 | 0.637 | 0.512 |
| L Thalamus | 0.683 | 0.024 | 0.628 | 0.64 | 0.685 | 0.72 | 0.726 | 0.765 | 0.532 | 0.022 | 0.481 | 0.493 | 0.533 | 0.567 | 0.572 | 0.225 |
| L Caudate | 0.719 | 0.022 | 0.669 | 0.679 | 0.72 | 0.752 | 0.756 | 0.709 | 0.559 | 0.024 | 0.509 | 0.517 | 0.56 | 0.595 | 0.601 | 0.314 |
| L Putamen | 0.705 | 0.023 | 0.653 | 0.664 | 0.706 | 0.74 | 0.745 | 0.705 | 0.567 | 0.024 | 0.515 | 0.524 | 0.568 | 0.603 | 0.608 | 0.362 |
| L Pallidum | 0.722 | 0.022 | 0.675 | 0.683 | 0.724 | 0.755 | 0.761 | 0.646 | 0.538 | 0.024 | 0.487 | 0.495 | 0.539 | 0.574 | 0.579 | 0.257 |
| L Hippocampus | 0.702 | 0.023 | 0.65 | 0.661 | 0.704 | 0.738 | 0.743 | 0.744 | 0.544 | 0.024 | 0.492 | 0.502 | 0.546 | 0.581 | 0.585 | 0.311 |
| L Amygdala | 0.729 | 0.021 | 0.682 | 0.692 | 0.731 | 0.761 | 0.766 | 0.676 | 0.569 | 0.023 | 0.52 | 0.529 | 0.57 | 0.604 | 0.609 | 0.323 |
| L Accumbens | 0.747 | 0.02 | 0.701 | 0.711 | 0.748 | 0.777 | 0.781 | 0.796 | 0.554 | 0.024 | 0.503 | 0.514 | 0.554 | 0.591 | 0.596 | 0.432 |
| R Thalamus | 0.683 | 0.025 | 0.63 | 0.639 | 0.685 | 0.721 | 0.726 | 0.724 | 0.531 | 0.022 | 0.48 | 0.49 | 0.532 | 0.564 | 0.57 | 0.324 |
| R Caudate | 0.725 | 0.022 | 0.677 | 0.686 | 0.727 | 0.757 | 0.763 | 0.737 | 0.551 | 0.024 | 0.5 | 0.508 | 0.552 | 0.59 | 0.595 | 0.37 |
| R Putamen | 0.712 | 0.022 | 0.66 | 0.672 | 0.714 | 0.746 | 0.75 | 0.721 | 0.567 | 0.023 | 0.517 | 0.526 | 0.568 | 0.604 | 0.61 | 0.459 |
| R Pallidum | 0.722 | 0.022 | 0.674 | 0.682 | 0.724 | 0.755 | 0.761 | 0.638 | 0.533 | 0.022 | 0.484 | 0.492 | 0.534 | 0.567 | 0.573 | 0.303 |
| R Hippocampus | 0.7 | 0.023 | 0.649 | 0.658 | 0.702 | 0.735 | 0.741 | 0.698 | 0.537 | 0.023 | 0.486 | 0.496 | 0.538 | 0.573 | 0.579 | 0.409 |
| R Amygdala | 0.712 | 0.022 | 0.662 | 0.672 | 0.714 | 0.746 | 0.751 | 0.654 | 0.571 | 0.024 | 0.522 | 0.53 | 0.572 | 0.609 | 0.614 | 0.393 |
| R Accumbens | 0.738 | 0.021 | 0.693 | 0.701 | 0.739 | 0.77 | 0.775 | 0.727 | 0.566 | 0.025 | 0.513 | 0.522 | 0.567 | 0.604 | 0.611 | 0.264 |

*Table S12.* ROI-level reliability for the face-emotion linear and quadratic slope contrasts: Bayesian multilevel model (BMM) and conventional linear mixed-effects model (ICC) for 200 cortical parcels and 14 subcortical ROIs. (ICC: blue=<.4, red=.4-.6, orange=.6-.75, yellow=.75-1)

|  | **Linear Slope** | | | | | | | | **Quadratic Slope** | | | | | | | |
| --- | --- | --- | --- | --- | --- | --- | --- | --- | --- | --- | --- | --- | --- | --- | --- | --- |
| **Label** | **BMM** | **SD** | **2.50%** | **5%** | **50%** | **95%** | **97.50%** | **ICC** | **BMM** | **SD** | **2.50%** | **5%** | **50%** | **95%** | **97.50%** | **ICC** |
| L VisCent ExStr 1 | 0.165 | 0.031 | 0.118 | 0.122 | 0.161 | 0.222 | 0.236 | 0.056 | 0.105 | 0.043 | 0.033 | 0.04 | 0.099 | 0.182 | 0.198 | 0.104 |
| L VisCent ExStr 2 | 0.154 | 0.027 | 0.114 | 0.118 | 0.149 | 0.206 | 0.216 | 0 | 0.179 | 0.046 | 0.092 | 0.105 | 0.177 | 0.257 | 0.276 | 0.071 |
| L VisCent Striate 1 | 0.206 | 0.039 | 0.137 | 0.146 | 0.205 | 0.272 | 0.283 | 0.108 | 0.109 | 0.045 | 0.034 | 0.043 | 0.105 | 0.189 | 0.206 | 0 |
| L VisCent ExStr 3 | 0.145 | 0.021 | 0.111 | 0.115 | 0.142 | 0.184 | 0.194 | 0.007 | 0.092 | 0.04 | 0.032 | 0.037 | 0.088 | 0.17 | 0.183 | 0.138 |
| L VisCent ExStr 4 | 0.148 | 0.024 | 0.112 | 0.117 | 0.144 | 0.192 | 0.204 | 0.062 | 0.069 | 0.032 | 0.028 | 0.031 | 0.062 | 0.13 | 0.144 | 0.127 |
| L VisCent ExStr 5 | 0.151 | 0.024 | 0.113 | 0.118 | 0.147 | 0.196 | 0.205 | 0.1 | 0.077 | 0.036 | 0.029 | 0.031 | 0.07 | 0.142 | 0.159 | 0.262 |
| L VisPeri ExStrInf 1 | 0.144 | 0.021 | 0.111 | 0.116 | 0.141 | 0.184 | 0.192 | 0.026 | 0.074 | 0.035 | 0.029 | 0.031 | 0.067 | 0.143 | 0.158 | 0.147 |
| L VisPeri ExStrInf 2 | 0.171 | 0.033 | 0.119 | 0.124 | 0.167 | 0.23 | 0.242 | 0.096 | 0.172 | 0.047 | 0.081 | 0.098 | 0.171 | 0.252 | 0.269 | 0.092 |
| L VisPeri ExStrInf 3 | 0.176 | 0.036 | 0.117 | 0.124 | 0.173 | 0.242 | 0.256 | 0 | 0.107 | 0.044 | 0.032 | 0.037 | 0.105 | 0.18 | 0.194 | 0 |
| L VisPeri StriCal 1 | 0.153 | 0.025 | 0.113 | 0.118 | 0.148 | 0.199 | 0.212 | 0.001 | 0.173 | 0.047 | 0.086 | 0.098 | 0.171 | 0.252 | 0.268 | 0.045 |
| L VisPeri ExStrSup 1 | 0.148 | 0.024 | 0.112 | 0.115 | 0.144 | 0.194 | 0.206 | 0 | 0.097 | 0.042 | 0.033 | 0.038 | 0.092 | 0.173 | 0.192 | 0.235 |
| L VisPeri ExStrSup 2 | 0.152 | 0.025 | 0.113 | 0.118 | 0.148 | 0.199 | 0.211 | 0 | 0.12 | 0.045 | 0.041 | 0.051 | 0.118 | 0.196 | 0.214 | 0.166 |
| L SomMotA 1 | 0.216 | 0.043 | 0.137 | 0.147 | 0.215 | 0.289 | 0.303 | 0.03 | 0.062 | 0.03 | 0.028 | 0.029 | 0.053 | 0.12 | 0.136 | 0 |
| L SomMotA 2 | 0.363 | 0.039 | 0.286 | 0.3 | 0.363 | 0.428 | 0.438 | 0.338 | 0.065 | 0.03 | 0.028 | 0.03 | 0.057 | 0.126 | 0.136 | 0.114 |
| L SomMotA 3 | 0.676 | 0.02 | 0.635 | 0.642 | 0.676 | 0.708 | 0.713 | 0.617 | 0.192 | 0.047 | 0.106 | 0.118 | 0.191 | 0.273 | 0.287 | 0.214 |
| L SomMotA 4 | 0.394 | 0.037 | 0.322 | 0.333 | 0.393 | 0.455 | 0.466 | 0.523 | 0.067 | 0.031 | 0.029 | 0.03 | 0.059 | 0.128 | 0.142 | 0.197 |
| L SomMotA 5 | 0.21 | 0.039 | 0.134 | 0.145 | 0.209 | 0.276 | 0.287 | 0.354 | 0.062 | 0.03 | 0.028 | 0.029 | 0.054 | 0.121 | 0.136 | 0.164 |
| L SomMotA 6 | 0.17 | 0.033 | 0.118 | 0.123 | 0.166 | 0.233 | 0.244 | 0.269 | 0.062 | 0.029 | 0.028 | 0.029 | 0.054 | 0.121 | 0.134 | 0.053 |
| L SomMotA 7 | 0.152 | 0.025 | 0.113 | 0.118 | 0.148 | 0.2 | 0.21 | 0.059 | 0.065 | 0.031 | 0.028 | 0.03 | 0.058 | 0.125 | 0.14 | 0 |
| L SomMotA 8 | 0.185 | 0.037 | 0.123 | 0.13 | 0.183 | 0.25 | 0.266 | 0.151 | 0.066 | 0.031 | 0.028 | 0.03 | 0.059 | 0.125 | 0.138 | 0 |
| L SomMotB Aud 1 | 0.152 | 0.026 | 0.114 | 0.119 | 0.148 | 0.201 | 0.214 | 0.008 | 0.128 | 0.049 | 0.04 | 0.051 | 0.126 | 0.212 | 0.231 | 0.128 |
| L SomMotB Aud 2 | 0.154 | 0.027 | 0.113 | 0.117 | 0.15 | 0.202 | 0.215 | 0.128 | 0.077 | 0.036 | 0.029 | 0.031 | 0.071 | 0.146 | 0.163 | 0.064 |
| L SomMotB S2 1 | 0.192 | 0.039 | 0.128 | 0.134 | 0.19 | 0.259 | 0.275 | 0.263 | 0.063 | 0.03 | 0.028 | 0.029 | 0.055 | 0.122 | 0.137 | 0 |
| L SomMotB S2 2 | 0.18 | 0.036 | 0.123 | 0.129 | 0.177 | 0.246 | 0.261 | 0.078 | 0.083 | 0.037 | 0.03 | 0.034 | 0.077 | 0.151 | 0.168 | 0.057 |
| L SomMotB Aud 3 | 0.146 | 0.022 | 0.111 | 0.116 | 0.143 | 0.188 | 0.195 | 0 | 0.069 | 0.032 | 0.028 | 0.03 | 0.062 | 0.13 | 0.147 | 0 |
| L SomMotB S2 3 | 0.285 | 0.04 | 0.207 | 0.219 | 0.285 | 0.352 | 0.364 | 0.288 | 0.075 | 0.035 | 0.03 | 0.032 | 0.069 | 0.142 | 0.157 | 0 |
| L SomMotB Cent 1 | 0.167 | 0.031 | 0.117 | 0.123 | 0.165 | 0.223 | 0.236 | 0.143 | 0.064 | 0.031 | 0.028 | 0.03 | 0.057 | 0.124 | 0.141 | 0 |
| L SomMotB Cent 2 | 0.164 | 0.032 | 0.115 | 0.121 | 0.16 | 0.223 | 0.233 | 0.177 | 0.061 | 0.029 | 0.028 | 0.029 | 0.052 | 0.115 | 0.132 | 0.095 |
| L DorsAttnA TempOcc 1 | 0.151 | 0.026 | 0.113 | 0.117 | 0.147 | 0.2 | 0.211 | 0.065 | 0.065 | 0.031 | 0.028 | 0.03 | 0.057 | 0.125 | 0.142 | 0 |
| L DorsAttnA TempOcc 2 | 0.16 | 0.03 | 0.116 | 0.12 | 0.156 | 0.218 | 0.231 | 0.039 | 0.064 | 0.03 | 0.028 | 0.03 | 0.057 | 0.123 | 0.139 | 0.127 |
| L DorsAttnA ParOcc 1 | 0.144 | 0.021 | 0.111 | 0.116 | 0.14 | 0.181 | 0.193 | 0 | 0.062 | 0.03 | 0.028 | 0.029 | 0.055 | 0.121 | 0.134 | 0.108 |
| L DorsAttnA SPL 1 | 0.144 | 0.021 | 0.111 | 0.116 | 0.141 | 0.184 | 0.193 | 0.068 | 0.063 | 0.03 | 0.028 | 0.03 | 0.055 | 0.121 | 0.137 | 0.163 |
| L DorsAttnA SPL 2 | 0.147 | 0.022 | 0.112 | 0.116 | 0.144 | 0.189 | 0.197 | 0.016 | 0.069 | 0.033 | 0.028 | 0.03 | 0.062 | 0.134 | 0.145 | 0 |
| L DorsAttnA SPL 3 | 0.214 | 0.038 | 0.144 | 0.153 | 0.212 | 0.278 | 0.292 | 0.136 | 0.088 | 0.039 | 0.031 | 0.035 | 0.084 | 0.16 | 0.178 | 0.263 |
| L DorsAttnB PostC 1 | 0.262 | 0.04 | 0.184 | 0.197 | 0.263 | 0.325 | 0.335 | 0.304 | 0.063 | 0.03 | 0.028 | 0.029 | 0.055 | 0.121 | 0.136 | 0 |
| L DorsAttnB PostC 2 | 0.273 | 0.038 | 0.2 | 0.212 | 0.272 | 0.336 | 0.348 | 0.28 | 0.064 | 0.03 | 0.028 | 0.03 | 0.058 | 0.123 | 0.138 | 0.146 |
| L DorsAttnB PostC 3 | 0.159 | 0.03 | 0.116 | 0.12 | 0.155 | 0.214 | 0.229 | 0.171 | 0.062 | 0.03 | 0.028 | 0.029 | 0.054 | 0.121 | 0.134 | 0.075 |
| L DorsAttnB PostC 4 | 0.151 | 0.024 | 0.113 | 0.117 | 0.147 | 0.196 | 0.205 | 0.142 | 0.064 | 0.03 | 0.028 | 0.03 | 0.057 | 0.123 | 0.139 | 0.059 |
| L DorsAttnB FEF 1 | 0.147 | 0.022 | 0.112 | 0.116 | 0.144 | 0.189 | 0.198 | 0.233 | 0.065 | 0.031 | 0.028 | 0.03 | 0.057 | 0.124 | 0.138 | 0 |
| L SalVentAttnA ParOper 1 | 0.173 | 0.034 | 0.121 | 0.126 | 0.17 | 0.235 | 0.247 | 0.114 | 0.063 | 0.03 | 0.028 | 0.029 | 0.055 | 0.121 | 0.132 | 0 |
| L SalVentAttnA Ins 1 | 0.143 | 0.02 | 0.11 | 0.115 | 0.139 | 0.18 | 0.189 | 0.207 | 0.065 | 0.031 | 0.028 | 0.03 | 0.058 | 0.126 | 0.138 | 0.023 |
| L SalVentAttnA FrOper 1 | 0.155 | 0.028 | 0.114 | 0.118 | 0.15 | 0.209 | 0.221 | 0.097 | 0.067 | 0.032 | 0.028 | 0.029 | 0.06 | 0.13 | 0.144 | 0 |
| L SalVentAttnA FrOper 2 | 0.155 | 0.027 | 0.114 | 0.119 | 0.151 | 0.206 | 0.219 | 0.099 | 0.155 | 0.047 | 0.065 | 0.081 | 0.154 | 0.234 | 0.246 | 0.087 |
| L SalVentAttnA ParMed 1 | 0.144 | 0.021 | 0.111 | 0.115 | 0.14 | 0.183 | 0.191 | 0.05 | 0.061 | 0.029 | 0.028 | 0.029 | 0.053 | 0.118 | 0.133 | 0 |
| L SalVentAttnA FrMed 1 | 0.197 | 0.041 | 0.124 | 0.132 | 0.195 | 0.268 | 0.282 | 0.207 | 0.076 | 0.035 | 0.03 | 0.032 | 0.069 | 0.144 | 0.16 | 0 |
| L SalVentAttnA FrMed 2 | 0.191 | 0.037 | 0.126 | 0.133 | 0.19 | 0.254 | 0.267 | 0.183 | 0.076 | 0.036 | 0.029 | 0.031 | 0.069 | 0.144 | 0.16 | 0 |
| L SalVentAttnB IPL 1 | 0.144 | 0.021 | 0.111 | 0.115 | 0.14 | 0.184 | 0.193 | 0 | 0.105 | 0.042 | 0.035 | 0.042 | 0.1 | 0.182 | 0.204 | 0 |
| L SalVentAttnB PFCl 1 | 0.144 | 0.021 | 0.111 | 0.115 | 0.14 | 0.182 | 0.19 | 0.111 | 0.064 | 0.03 | 0.028 | 0.03 | 0.057 | 0.121 | 0.135 | 0 |
| L SalVentAttnB Ins 1 | 0.281 | 0.037 | 0.211 | 0.222 | 0.28 | 0.342 | 0.357 | 0.272 | 0.293 | 0.045 | 0.205 | 0.217 | 0.294 | 0.368 | 0.384 | 0.312 |
| L SalVentAttnB PFCmp 1 | 0.154 | 0.027 | 0.113 | 0.117 | 0.15 | 0.203 | 0.215 | 0.033 | 0.099 | 0.042 | 0.031 | 0.037 | 0.094 | 0.172 | 0.185 | 0.096 |
| L LimbicB OFC 1 | 0.211 | 0.04 | 0.136 | 0.145 | 0.21 | 0.28 | 0.295 | 0.071 | 0.175 | 0.048 | 0.084 | 0.097 | 0.174 | 0.254 | 0.271 | 0.16 |
| L LimbicB OFC 2 | 0.269 | 0.042 | 0.189 | 0.201 | 0.267 | 0.338 | 0.353 | 0 | 0.214 | 0.046 | 0.129 | 0.141 | 0.213 | 0.293 | 0.307 | 0.102 |
| L LimbicA TempPole 1 | 0.269 | 0.042 | 0.189 | 0.201 | 0.267 | 0.338 | 0.353 | 0.306 | 0.214 | 0.046 | 0.129 | 0.141 | 0.213 | 0.293 | 0.307 | 0.22 |
| L LimbicA TempPole 2 | 0.171 | 0.032 | 0.12 | 0.126 | 0.167 | 0.23 | 0.24 | 0.243 | 0.072 | 0.034 | 0.029 | 0.031 | 0.064 | 0.136 | 0.152 | 0 |
| L LimbicA TempPole 3 | 0.471 | 0.034 | 0.405 | 0.415 | 0.472 | 0.525 | 0.536 | 0 | 0.496 | 0.036 | 0.425 | 0.438 | 0.497 | 0.553 | 0.565 | 0 |
| L LimbicA TempPole 4 | 0.333 | 0.039 | 0.256 | 0.269 | 0.332 | 0.397 | 0.409 | 0 | 0.29 | 0.049 | 0.203 | 0.212 | 0.289 | 0.37 | 0.386 | 0 |
| L ContA Temp 1 | 0.15 | 0.024 | 0.113 | 0.117 | 0.146 | 0.193 | 0.203 | 0 | 0.063 | 0.03 | 0.028 | 0.029 | 0.056 | 0.122 | 0.135 | 0.031 |
| L ContA IPS 1 | 0.158 | 0.028 | 0.116 | 0.121 | 0.154 | 0.209 | 0.22 | 0.15 | 0.072 | 0.034 | 0.029 | 0.031 | 0.067 | 0.136 | 0.155 | 0 |
| L ContA IPS 2 | 0.171 | 0.032 | 0.119 | 0.125 | 0.168 | 0.228 | 0.24 | 0.205 | 0.087 | 0.039 | 0.031 | 0.034 | 0.083 | 0.158 | 0.177 | 0.1 |
| L ContA IPS 3 | 0.147 | 0.022 | 0.112 | 0.117 | 0.144 | 0.187 | 0.194 | 0 | 0.072 | 0.034 | 0.029 | 0.031 | 0.065 | 0.137 | 0.155 | 0.012 |
| L ContA PFCd 1 | 0.142 | 0.02 | 0.11 | 0.114 | 0.139 | 0.181 | 0.188 | 0.214 | 0.071 | 0.034 | 0.029 | 0.031 | 0.064 | 0.134 | 0.15 | 0 |
| L ContA PFClv 1 | 0.143 | 0.021 | 0.11 | 0.115 | 0.14 | 0.181 | 0.189 | 0.096 | 0.072 | 0.032 | 0.029 | 0.031 | 0.067 | 0.131 | 0.147 | 0 |
| L ContA PFCl 1 | 0.144 | 0.021 | 0.111 | 0.115 | 0.141 | 0.184 | 0.192 | 0.15 | 0.086 | 0.039 | 0.031 | 0.034 | 0.08 | 0.161 | 0.175 | 0 |
| L ContA PFCl 2 | 0.182 | 0.035 | 0.123 | 0.129 | 0.179 | 0.244 | 0.257 | 0.375 | 0.146 | 0.048 | 0.051 | 0.068 | 0.144 | 0.229 | 0.246 | 0.058 |
| L ContA PFCl 3 | 0.154 | 0.026 | 0.115 | 0.119 | 0.15 | 0.203 | 0.217 | 0.281 | 0.167 | 0.049 | 0.075 | 0.089 | 0.165 | 0.249 | 0.262 | 0.339 |
| L ContA Cingm 1 | 0.186 | 0.037 | 0.126 | 0.13 | 0.184 | 0.252 | 0.265 | 0.16 | 0.245 | 0.047 | 0.158 | 0.169 | 0.244 | 0.327 | 0.339 | 0.093 |
| L ContB Temp 1 | 0.185 | 0.038 | 0.123 | 0.128 | 0.181 | 0.253 | 0.267 | 0.158 | 0.13 | 0.045 | 0.05 | 0.061 | 0.127 | 0.207 | 0.225 | 0.049 |
| L ContB IPL 1 | 0.148 | 0.023 | 0.113 | 0.116 | 0.145 | 0.19 | 0.2 | 0.073 | 0.133 | 0.049 | 0.043 | 0.056 | 0.13 | 0.217 | 0.233 | 0.079 |
| L ContB PFCl 1 | 0.152 | 0.025 | 0.113 | 0.118 | 0.148 | 0.2 | 0.209 | 0.199 | 0.113 | 0.045 | 0.037 | 0.044 | 0.108 | 0.191 | 0.208 | 0.071 |
| L ContB PFClv 1 | 0.225 | 0.04 | 0.152 | 0.162 | 0.224 | 0.291 | 0.308 | 0.139 | 0.182 | 0.047 | 0.097 | 0.11 | 0.18 | 0.263 | 0.284 | 0 |
| L ContB PFClv 2 | 0.147 | 0.022 | 0.111 | 0.116 | 0.143 | 0.187 | 0.198 | 0.069 | 0.081 | 0.038 | 0.03 | 0.032 | 0.075 | 0.15 | 0.169 | 0.075 |
| L ContC pCun 1 | 0.155 | 0.027 | 0.114 | 0.118 | 0.152 | 0.205 | 0.217 | 0 | 0.142 | 0.046 | 0.054 | 0.066 | 0.141 | 0.22 | 0.234 | 0 |
| L ContC pCun 2 | 0.146 | 0.022 | 0.11 | 0.115 | 0.143 | 0.188 | 0.197 | 0 | 0.09 | 0.04 | 0.032 | 0.035 | 0.084 | 0.16 | 0.179 | 0.146 |
| L ContC Cingp 1 | 0.265 | 0.039 | 0.189 | 0.2 | 0.266 | 0.328 | 0.343 | 0.169 | 0.225 | 0.045 | 0.138 | 0.152 | 0.225 | 0.299 | 0.313 | 0.016 |
| L DefaultA IPL 1 | 0.145 | 0.022 | 0.111 | 0.115 | 0.141 | 0.186 | 0.196 | 0 | 0.151 | 0.048 | 0.066 | 0.076 | 0.15 | 0.233 | 0.251 | 0.162 |
| L DefaultA PFCd 1 | 0.146 | 0.022 | 0.111 | 0.115 | 0.142 | 0.186 | 0.195 | 0.161 | 0.078 | 0.036 | 0.029 | 0.031 | 0.072 | 0.147 | 0.161 | 0.09 |
| L DefaultA pCunPCC 1 | 0.16 | 0.029 | 0.116 | 0.121 | 0.155 | 0.213 | 0.223 | 0 | 0.172 | 0.046 | 0.087 | 0.099 | 0.17 | 0.25 | 0.263 | 0 |
| L DefaultA pCunPCC 2 | 0.16 | 0.029 | 0.116 | 0.121 | 0.156 | 0.214 | 0.227 | 0 | 0.174 | 0.047 | 0.088 | 0.099 | 0.171 | 0.252 | 0.268 | 0.065 |
| L DefaultA pCunPCC 3 | 0.144 | 0.021 | 0.11 | 0.115 | 0.141 | 0.183 | 0.194 | 0 | 0.126 | 0.046 | 0.044 | 0.054 | 0.124 | 0.205 | 0.217 | 0.119 |
| L DefaultA PFCm 1 | 0.286 | 0.04 | 0.209 | 0.219 | 0.286 | 0.353 | 0.363 | 0 | 0.296 | 0.049 | 0.203 | 0.217 | 0.295 | 0.379 | 0.392 | 0.099 |
| L DefaultA PFCm 2 | 0.149 | 0.024 | 0.112 | 0.117 | 0.146 | 0.192 | 0.203 | 0 | 0.08 | 0.037 | 0.03 | 0.032 | 0.074 | 0.15 | 0.167 | 0.015 |
| L DefaultA PFCm 3 | 0.148 | 0.023 | 0.112 | 0.116 | 0.144 | 0.191 | 0.201 | 0 | 0.091 | 0.041 | 0.03 | 0.034 | 0.086 | 0.168 | 0.185 | 0.067 |
| L DefaultB Temp 1 | 0.165 | 0.031 | 0.118 | 0.122 | 0.162 | 0.224 | 0.234 | 0.134 | 0.067 | 0.032 | 0.028 | 0.03 | 0.06 | 0.127 | 0.144 | 0.059 |
| L DefaultB Temp 2 | 0.21 | 0.04 | 0.14 | 0.148 | 0.208 | 0.279 | 0.292 | 0.122 | 0.101 | 0.043 | 0.033 | 0.038 | 0.096 | 0.178 | 0.194 | 0.034 |
| L DefaultB Temp 3 | 0.145 | 0.021 | 0.111 | 0.116 | 0.142 | 0.183 | 0.191 | 0.138 | 0.066 | 0.031 | 0.028 | 0.03 | 0.059 | 0.126 | 0.145 | 0.123 |
| L DefaultB Temp 4 | 0.149 | 0.024 | 0.112 | 0.117 | 0.146 | 0.192 | 0.205 | 0.127 | 0.071 | 0.033 | 0.028 | 0.03 | 0.064 | 0.133 | 0.146 | 0.195 |
| L DefaultB IPL 1 | 0.155 | 0.027 | 0.115 | 0.119 | 0.15 | 0.204 | 0.22 | 0.021 | 0.085 | 0.038 | 0.031 | 0.035 | 0.079 | 0.158 | 0.173 | 0.118 |
| L DefaultB PFCd 1 | 0.143 | 0.021 | 0.11 | 0.114 | 0.14 | 0.181 | 0.19 | 0 | 0.074 | 0.035 | 0.029 | 0.031 | 0.067 | 0.14 | 0.157 | 0.01 |
| L DefaultB PFCd 2 | 0.215 | 0.041 | 0.137 | 0.148 | 0.212 | 0.285 | 0.297 | 0.068 | 0.121 | 0.046 | 0.041 | 0.051 | 0.117 | 0.203 | 0.217 | 0.113 |
| L DefaultB PFCd 3 | 0.2 | 0.039 | 0.131 | 0.138 | 0.198 | 0.268 | 0.281 | 0.194 | 0.305 | 0.046 | 0.212 | 0.23 | 0.306 | 0.38 | 0.392 | 0.297 |
| L DefaultB PFCd 4 | 0.15 | 0.025 | 0.112 | 0.117 | 0.146 | 0.196 | 0.206 | 0.198 | 0.222 | 0.046 | 0.132 | 0.146 | 0.221 | 0.298 | 0.314 | 0.21 |
| L DefaultB PFCv 1 | 0.148 | 0.023 | 0.111 | 0.115 | 0.144 | 0.19 | 0.199 | 0.067 | 0.138 | 0.046 | 0.051 | 0.063 | 0.137 | 0.218 | 0.235 | 0.011 |
| L DefaultB PFCv 2 | 0.182 | 0.037 | 0.122 | 0.128 | 0.179 | 0.246 | 0.258 | 0.097 | 0.077 | 0.036 | 0.029 | 0.032 | 0.071 | 0.146 | 0.166 | 0 |
| L DefaultB PFCv 3 | 0.155 | 0.027 | 0.114 | 0.119 | 0.151 | 0.204 | 0.217 | 0.026 | 0.161 | 0.045 | 0.079 | 0.091 | 0.159 | 0.237 | 0.249 | 0.081 |
| L DefaultB PFCv 4 | 0.169 | 0.032 | 0.118 | 0.124 | 0.165 | 0.229 | 0.243 | 0.195 | 0.224 | 0.047 | 0.132 | 0.148 | 0.225 | 0.3 | 0.316 | 0.314 |
| L DefaultC IPL 1 | 0.149 | 0.024 | 0.113 | 0.117 | 0.145 | 0.195 | 0.206 | 0.063 | 0.102 | 0.043 | 0.033 | 0.038 | 0.097 | 0.175 | 0.191 | 0.108 |
| L DefaultC Rsp 1 | 0.203 | 0.037 | 0.133 | 0.144 | 0.202 | 0.268 | 0.28 | 0.006 | 0.235 | 0.045 | 0.15 | 0.161 | 0.235 | 0.31 | 0.324 | 0 |
| L DefaultC PHC 1 | 0.198 | 0.038 | 0.133 | 0.14 | 0.196 | 0.264 | 0.279 | 0.35 | 0.11 | 0.046 | 0.036 | 0.041 | 0.107 | 0.19 | 0.209 | 0.037 |
| L TempPar 1 | 0.146 | 0.022 | 0.112 | 0.116 | 0.143 | 0.188 | 0.195 | 0.069 | 0.068 | 0.033 | 0.028 | 0.03 | 0.06 | 0.131 | 0.146 | 0.073 |
| L TempPar 2 | 0.146 | 0.022 | 0.11 | 0.115 | 0.142 | 0.185 | 0.195 | 0.023 | 0.065 | 0.031 | 0.029 | 0.03 | 0.057 | 0.127 | 0.143 | 0 |
| R VisCent ExStr 1 | 0.149 | 0.024 | 0.113 | 0.117 | 0.145 | 0.193 | 0.203 | 0.082 | 0.063 | 0.03 | 0.028 | 0.03 | 0.056 | 0.119 | 0.133 | 0.016 |
| R VisCent ExStr 2 | 0.181 | 0.035 | 0.123 | 0.129 | 0.178 | 0.245 | 0.258 | 0.088 | 0.063 | 0.03 | 0.028 | 0.029 | 0.056 | 0.122 | 0.138 | 0 |
| R VisCent Striate 1 | 0.267 | 0.038 | 0.194 | 0.206 | 0.267 | 0.333 | 0.343 | 0.076 | 0.189 | 0.047 | 0.102 | 0.116 | 0.188 | 0.267 | 0.28 | 0.011 |
| R VisCent ExStr 3 | 0.257 | 0.044 | 0.17 | 0.186 | 0.256 | 0.331 | 0.346 | 0.202 | 0.209 | 0.047 | 0.12 | 0.135 | 0.208 | 0.288 | 0.307 | 0.096 |
| R VisCent ExStr 4 | 0.146 | 0.022 | 0.112 | 0.116 | 0.142 | 0.185 | 0.196 | 0.036 | 0.064 | 0.03 | 0.028 | 0.03 | 0.056 | 0.122 | 0.138 | 0.125 |
| R VisCent ExStr 5 | 0.164 | 0.031 | 0.116 | 0.122 | 0.161 | 0.218 | 0.231 | 0.253 | 0.071 | 0.033 | 0.029 | 0.03 | 0.064 | 0.134 | 0.147 | 0.205 |
| R VisPeri ExStrInf 1 | 0.146 | 0.022 | 0.111 | 0.116 | 0.143 | 0.188 | 0.198 | 0.125 | 0.098 | 0.042 | 0.032 | 0.036 | 0.094 | 0.171 | 0.189 | 0.151 |
| R VisPeri ExStrInf 2 | 0.192 | 0.035 | 0.129 | 0.137 | 0.189 | 0.253 | 0.263 | 0.059 | 0.17 | 0.045 | 0.086 | 0.095 | 0.169 | 0.246 | 0.262 | 0.136 |
| R VisPeri StriCal 1 | 0.156 | 0.026 | 0.116 | 0.12 | 0.151 | 0.205 | 0.215 | 0.015 | 0.17 | 0.046 | 0.084 | 0.096 | 0.17 | 0.249 | 0.262 | 0.096 |
| R VisPeri ExStrSup 1 | 0.143 | 0.02 | 0.109 | 0.115 | 0.139 | 0.181 | 0.19 | 0 | 0.166 | 0.047 | 0.075 | 0.089 | 0.165 | 0.248 | 0.262 | 0.2 |
| R VisPeri ExStrSup 2 | 0.145 | 0.022 | 0.111 | 0.116 | 0.142 | 0.184 | 0.197 | 0.015 | 0.086 | 0.037 | 0.032 | 0.035 | 0.081 | 0.153 | 0.17 | 0.151 |
| R VisPeri ExStrSup 3 | 0.216 | 0.041 | 0.14 | 0.15 | 0.214 | 0.287 | 0.303 | 0.177 | 0.167 | 0.045 | 0.083 | 0.095 | 0.165 | 0.243 | 0.262 | 0.129 |
| R SomMotA 1 | 0.526 | 0.03 | 0.467 | 0.476 | 0.527 | 0.572 | 0.583 | 0.369 | 0.066 | 0.032 | 0.028 | 0.03 | 0.059 | 0.125 | 0.14 | 0 |
| R SomMotA 2 | 0.445 | 0.035 | 0.377 | 0.387 | 0.446 | 0.503 | 0.512 | 0.263 | 0.076 | 0.035 | 0.029 | 0.031 | 0.07 | 0.143 | 0.157 | 0.044 |
| R SomMotA 3 | 0.265 | 0.041 | 0.187 | 0.198 | 0.264 | 0.331 | 0.344 | 0.1 | 0.064 | 0.03 | 0.028 | 0.029 | 0.057 | 0.124 | 0.138 | 0.046 |
| R SomMotA 4 | 0.727 | 0.016 | 0.694 | 0.7 | 0.727 | 0.753 | 0.758 | 0.369 | 0.198 | 0.05 | 0.104 | 0.117 | 0.197 | 0.28 | 0.293 | 0.049 |
| R SomMotA 5 | 0.29 | 0.041 | 0.214 | 0.224 | 0.291 | 0.359 | 0.37 | 0.143 | 0.09 | 0.041 | 0.032 | 0.036 | 0.084 | 0.166 | 0.183 | 0.066 |
| R SomMotA 6 | 0.552 | 0.03 | 0.49 | 0.501 | 0.553 | 0.601 | 0.609 | 0.258 | 0.087 | 0.04 | 0.031 | 0.034 | 0.08 | 0.162 | 0.179 | 0.059 |
| R SomMotA 7 | 0.259 | 0.04 | 0.185 | 0.194 | 0.258 | 0.324 | 0.34 | 0.166 | 0.063 | 0.03 | 0.028 | 0.029 | 0.056 | 0.122 | 0.136 | 0.027 |
| R SomMotA 8 | 0.188 | 0.035 | 0.127 | 0.134 | 0.187 | 0.249 | 0.259 | 0.141 | 0.064 | 0.03 | 0.028 | 0.029 | 0.056 | 0.122 | 0.137 | 0 |
| R SomMotA 9 | 0.146 | 0.023 | 0.111 | 0.116 | 0.143 | 0.188 | 0.197 | 0 | 0.061 | 0.029 | 0.028 | 0.029 | 0.051 | 0.118 | 0.132 | 0.011 |
| R SomMotA 10 | 0.158 | 0.029 | 0.114 | 0.119 | 0.154 | 0.215 | 0.225 | 0 | 0.062 | 0.03 | 0.028 | 0.029 | 0.054 | 0.121 | 0.135 | 0 |
| R SomMotA 11 | 0.277 | 0.042 | 0.2 | 0.209 | 0.278 | 0.347 | 0.362 | 0.132 | 0.077 | 0.035 | 0.029 | 0.031 | 0.072 | 0.147 | 0.16 | 0.081 |
| R SomMotB Aud 1 | 0.172 | 0.034 | 0.118 | 0.124 | 0.169 | 0.231 | 0.25 | 0.141 | 0.101 | 0.045 | 0.032 | 0.037 | 0.096 | 0.183 | 0.202 | 0.051 |
| R SomMotB Aud 2 | 0.145 | 0.021 | 0.111 | 0.115 | 0.142 | 0.184 | 0.193 | 0.088 | 0.083 | 0.038 | 0.03 | 0.033 | 0.078 | 0.153 | 0.172 | 0.031 |
| R SomMotB S2 1 | 0.189 | 0.037 | 0.122 | 0.131 | 0.186 | 0.254 | 0.264 | 0.025 | 0.063 | 0.03 | 0.028 | 0.029 | 0.055 | 0.121 | 0.134 | 0 |
| R SomMotB S2 2 | 0.233 | 0.043 | 0.148 | 0.163 | 0.233 | 0.303 | 0.32 | 0.186 | 0.125 | 0.045 | 0.049 | 0.055 | 0.122 | 0.201 | 0.219 | 0.087 |
| R SomMotB S2 3 | 0.234 | 0.04 | 0.155 | 0.165 | 0.234 | 0.301 | 0.312 | 0.13 | 0.097 | 0.04 | 0.033 | 0.039 | 0.093 | 0.17 | 0.185 | 0 |
| R SomMotB S2 4 | 0.308 | 0.042 | 0.227 | 0.238 | 0.308 | 0.374 | 0.385 | 0.158 | 0.074 | 0.036 | 0.029 | 0.031 | 0.068 | 0.143 | 0.163 | 0 |
| R SomMotB Cent 1 | 0.183 | 0.038 | 0.12 | 0.127 | 0.18 | 0.249 | 0.262 | 0 | 0.079 | 0.036 | 0.03 | 0.033 | 0.073 | 0.148 | 0.163 | 0.098 |
| R DorsAttnA TempOcc 1 | 0.147 | 0.023 | 0.111 | 0.116 | 0.144 | 0.19 | 0.202 | 0.103 | 0.062 | 0.03 | 0.028 | 0.029 | 0.055 | 0.122 | 0.134 | 0 |
| R DorsAttnA ParOcc 1 | 0.142 | 0.02 | 0.111 | 0.115 | 0.139 | 0.18 | 0.188 | 0 | 0.061 | 0.029 | 0.028 | 0.029 | 0.053 | 0.119 | 0.132 | 0.028 |
| R DorsAttnA SPL 1 | 0.145 | 0.021 | 0.111 | 0.116 | 0.142 | 0.184 | 0.193 | 0.095 | 0.062 | 0.029 | 0.028 | 0.029 | 0.054 | 0.118 | 0.133 | 0.133 |
| R DorsAttnA SPL 2 | 0.192 | 0.038 | 0.128 | 0.135 | 0.19 | 0.261 | 0.272 | 0.009 | 0.149 | 0.046 | 0.063 | 0.076 | 0.147 | 0.228 | 0.242 | 0.086 |
| R DorsAttnA SPL 3 | 0.16 | 0.028 | 0.116 | 0.122 | 0.155 | 0.212 | 0.225 | 0.135 | 0.066 | 0.031 | 0.028 | 0.03 | 0.058 | 0.124 | 0.138 | 0.106 |
| R DorsAttnA SPL 4 | 0.151 | 0.025 | 0.112 | 0.117 | 0.147 | 0.2 | 0.209 | 0 | 0.083 | 0.037 | 0.03 | 0.034 | 0.077 | 0.153 | 0.17 | 0.256 |
| R DorsAttnB PostC 1 | 0.239 | 0.039 | 0.168 | 0.176 | 0.238 | 0.302 | 0.317 | 0.147 | 0.063 | 0.03 | 0.028 | 0.029 | 0.054 | 0.12 | 0.133 | 0 |
| R DorsAttnB PostC 2 | 0.22 | 0.039 | 0.149 | 0.159 | 0.219 | 0.287 | 0.296 | 0.168 | 0.067 | 0.031 | 0.028 | 0.03 | 0.06 | 0.127 | 0.144 | 0 |
| R DorsAttnB PostC 3 | 0.143 | 0.02 | 0.11 | 0.115 | 0.14 | 0.181 | 0.189 | 0 | 0.064 | 0.03 | 0.028 | 0.03 | 0.056 | 0.122 | 0.138 | 0.187 |
| R DorsAttnB PostC 4 | 0.185 | 0.035 | 0.125 | 0.131 | 0.182 | 0.246 | 0.259 | 0.03 | 0.067 | 0.032 | 0.028 | 0.03 | 0.06 | 0.127 | 0.147 | 0.101 |
| R DorsAttnB FEF 1 | 0.22 | 0.036 | 0.155 | 0.162 | 0.219 | 0.28 | 0.294 | 0.179 | 0.064 | 0.031 | 0.028 | 0.029 | 0.055 | 0.122 | 0.139 | 0.175 |
| R SalVentAttnA ParOper 1 | 0.182 | 0.037 | 0.123 | 0.128 | 0.178 | 0.248 | 0.262 | 0.139 | 0.063 | 0.03 | 0.028 | 0.029 | 0.056 | 0.121 | 0.136 | 0 |
| R SalVentAttnA PrC 1 | 0.187 | 0.035 | 0.127 | 0.133 | 0.184 | 0.248 | 0.258 | 0 | 0.079 | 0.037 | 0.03 | 0.032 | 0.074 | 0.147 | 0.163 | 0.053 |
| R SalVentAttnA Ins 1 | 0.185 | 0.038 | 0.123 | 0.131 | 0.181 | 0.251 | 0.265 | 0 | 0.125 | 0.045 | 0.046 | 0.056 | 0.121 | 0.205 | 0.216 | 0.044 |
| R SalVentAttnA Ins 2 | 0.146 | 0.022 | 0.112 | 0.116 | 0.142 | 0.186 | 0.195 | 0.112 | 0.094 | 0.042 | 0.031 | 0.034 | 0.089 | 0.173 | 0.186 | 0.027 |
| R SalVentAttnA FrOper 1 | 0.148 | 0.024 | 0.11 | 0.116 | 0.145 | 0.193 | 0.202 | 0.087 | 0.123 | 0.045 | 0.045 | 0.055 | 0.119 | 0.203 | 0.219 | 0.069 |
| R SalVentAttnA FrMed 1 | 0.157 | 0.028 | 0.114 | 0.119 | 0.153 | 0.209 | 0.22 | 0 | 0.07 | 0.032 | 0.028 | 0.031 | 0.062 | 0.131 | 0.148 | 0.102 |
| R SalVentAttnA ParMed 1 | 0.147 | 0.022 | 0.112 | 0.117 | 0.144 | 0.187 | 0.197 | 0.094 | 0.061 | 0.029 | 0.028 | 0.029 | 0.053 | 0.118 | 0.133 | 0 |
| R SalVentAttnA ParMed 2 | 0.143 | 0.021 | 0.11 | 0.116 | 0.14 | 0.183 | 0.19 | 0 | 0.061 | 0.029 | 0.028 | 0.029 | 0.053 | 0.118 | 0.132 | 0.101 |
| R SalVentAttnA FrMed 2 | 0.155 | 0.027 | 0.114 | 0.119 | 0.151 | 0.203 | 0.218 | 0 | 0.082 | 0.039 | 0.03 | 0.033 | 0.076 | 0.155 | 0.17 | 0.079 |
| R SalVentAttnB IPL 1 | 0.156 | 0.027 | 0.115 | 0.119 | 0.152 | 0.207 | 0.218 | 0.013 | 0.084 | 0.038 | 0.031 | 0.033 | 0.078 | 0.157 | 0.174 | 0.031 |
| R SalVentAttnB PFClv 1 | 0.174 | 0.034 | 0.118 | 0.124 | 0.171 | 0.237 | 0.249 | 0.082 | 0.082 | 0.038 | 0.03 | 0.033 | 0.075 | 0.153 | 0.169 | 0.129 |
| R SalVentAttnB PFCl 1 | 0.148 | 0.023 | 0.111 | 0.116 | 0.145 | 0.189 | 0.198 | 0 | 0.065 | 0.031 | 0.028 | 0.03 | 0.058 | 0.125 | 0.139 | 0.04 |
| R SalVentAttnB Ins 1 | 0.166 | 0.03 | 0.118 | 0.123 | 0.163 | 0.222 | 0.234 | 0.209 | 0.227 | 0.046 | 0.14 | 0.15 | 0.225 | 0.305 | 0.319 | 0.135 |
| R SalVentAttnB Ins 2 | 0.223 | 0.039 | 0.151 | 0.161 | 0.222 | 0.29 | 0.3 | 0.151 | 0.301 | 0.044 | 0.212 | 0.226 | 0.302 | 0.373 | 0.385 | 0.221 |
| R SalVentAttnB PFCmp 1 | 0.151 | 0.025 | 0.113 | 0.117 | 0.148 | 0.197 | 0.207 | 0.048 | 0.083 | 0.038 | 0.03 | 0.034 | 0.077 | 0.15 | 0.168 | 0.179 |
| R LimbicB OFC 2 | 0.286 | 0.042 | 0.208 | 0.219 | 0.285 | 0.357 | 0.368 | 0.183 | 0.119 | 0.044 | 0.043 | 0.051 | 0.116 | 0.197 | 0.214 | 0.016 |
| R LimbicB OFC 3 | 0.261 | 0.04 | 0.184 | 0.194 | 0.26 | 0.33 | 0.342 | 0.048 | 0.251 | 0.049 | 0.15 | 0.165 | 0.252 | 0.328 | 0.343 | 0.164 |
| R LimbicB OFC 4 | 0.167 | 0.031 | 0.117 | 0.123 | 0.164 | 0.224 | 0.236 | 0 | 0.07 | 0.033 | 0.029 | 0.031 | 0.063 | 0.135 | 0.148 | 0 |
| R LimbicA TempPole 1 | 0.272 | 0.041 | 0.194 | 0.206 | 0.272 | 0.34 | 0.35 | 0.006 | 0.29 | 0.05 | 0.192 | 0.209 | 0.29 | 0.372 | 0.392 | 0 |
| R LimbicA TempPole 2 | 0.151 | 0.026 | 0.112 | 0.117 | 0.147 | 0.198 | 0.211 | 0.056 | 0.1 | 0.045 | 0.032 | 0.036 | 0.097 | 0.181 | 0.199 | 0.059 |
| R LimbicA TempPole 3 | 0.508 | 0.034 | 0.441 | 0.451 | 0.509 | 0.563 | 0.57 | 0.018 | 0.424 | 0.042 | 0.338 | 0.354 | 0.425 | 0.49 | 0.504 | 0.084 |
| R LimbicA TempPole 4 | 0.158 | 0.028 | 0.116 | 0.121 | 0.154 | 0.206 | 0.221 | 0.201 | 0.063 | 0.03 | 0.028 | 0.029 | 0.056 | 0.123 | 0.138 | 0 |
| R ContA IPS 1 | 0.186 | 0.037 | 0.124 | 0.131 | 0.183 | 0.253 | 0.265 | 0.17 | 0.083 | 0.037 | 0.03 | 0.033 | 0.078 | 0.15 | 0.168 | 0 |
| R ContA IPS 2 | 0.163 | 0.029 | 0.118 | 0.123 | 0.159 | 0.218 | 0.227 | 0.183 | 0.082 | 0.038 | 0.03 | 0.032 | 0.075 | 0.155 | 0.172 | 0 |
| R ContA PFCd 1 | 0.142 | 0.02 | 0.11 | 0.114 | 0.139 | 0.179 | 0.188 | 0 | 0.072 | 0.034 | 0.029 | 0.03 | 0.065 | 0.138 | 0.155 | 0 |
| R ContA PFCl 1 | 0.147 | 0.022 | 0.113 | 0.117 | 0.144 | 0.187 | 0.195 | 0.073 | 0.116 | 0.045 | 0.038 | 0.046 | 0.112 | 0.194 | 0.209 | 0.101 |
| R ContA PFCl 2 | 0.162 | 0.03 | 0.117 | 0.121 | 0.158 | 0.215 | 0.231 | 0 | 0.179 | 0.048 | 0.087 | 0.102 | 0.177 | 0.26 | 0.277 | 0.162 |
| R ContA Cingm 1 | 0.144 | 0.021 | 0.111 | 0.115 | 0.141 | 0.182 | 0.191 | 0.146 | 0.065 | 0.031 | 0.028 | 0.03 | 0.058 | 0.124 | 0.138 | 0.118 |
| R ContB Temp 1 | 0.233 | 0.041 | 0.153 | 0.166 | 0.233 | 0.3 | 0.312 | 0.117 | 0.197 | 0.05 | 0.1 | 0.117 | 0.196 | 0.279 | 0.296 | 0.111 |
| R ContB Temp 2 | 0.236 | 0.04 | 0.159 | 0.17 | 0.237 | 0.303 | 0.318 | 0.216 | 0.157 | 0.048 | 0.066 | 0.081 | 0.156 | 0.238 | 0.251 | 0.118 |
| R ContB IPL 1 | 0.175 | 0.033 | 0.123 | 0.128 | 0.171 | 0.233 | 0.246 | 0.034 | 0.249 | 0.046 | 0.161 | 0.172 | 0.249 | 0.324 | 0.338 | 0.257 |
| R ContB IPL 2 | 0.21 | 0.039 | 0.141 | 0.151 | 0.209 | 0.277 | 0.289 | 0.226 | 0.173 | 0.047 | 0.087 | 0.097 | 0.171 | 0.25 | 0.266 | 0.173 |
| R ContB PFCld 1 | 0.161 | 0.029 | 0.117 | 0.121 | 0.155 | 0.214 | 0.228 | 0 | 0.142 | 0.047 | 0.057 | 0.069 | 0.14 | 0.219 | 0.236 | 0.172 |
| R ContB PFCld 2 | 0.15 | 0.025 | 0.114 | 0.117 | 0.146 | 0.196 | 0.207 | 0 | 0.084 | 0.04 | 0.03 | 0.033 | 0.079 | 0.161 | 0.177 | 0.103 |
| R ContB PFClv 1 | 0.202 | 0.041 | 0.128 | 0.138 | 0.2 | 0.275 | 0.292 | 0.216 | 0.081 | 0.039 | 0.03 | 0.032 | 0.073 | 0.155 | 0.175 | 0.026 |
| R ContB PFClv 2 | 0.164 | 0.03 | 0.118 | 0.122 | 0.16 | 0.218 | 0.228 | 0.062 | 0.081 | 0.037 | 0.03 | 0.033 | 0.077 | 0.149 | 0.164 | 0.145 |
| R ContB PFCmp 1 | 0.15 | 0.025 | 0.113 | 0.117 | 0.146 | 0.197 | 0.207 | 0.11 | 0.211 | 0.049 | 0.115 | 0.129 | 0.211 | 0.293 | 0.308 | 0.381 |
| R ContB PFCld 3 | 0.152 | 0.025 | 0.113 | 0.117 | 0.148 | 0.199 | 0.209 | 0.247 | 0.089 | 0.039 | 0.031 | 0.035 | 0.082 | 0.162 | 0.18 | 0.019 |
| R ContC pCun 1 | 0.165 | 0.031 | 0.118 | 0.123 | 0.161 | 0.222 | 0.234 | 0.001 | 0.068 | 0.032 | 0.028 | 0.03 | 0.062 | 0.128 | 0.145 | 0 |
| R ContC pCun 2 | 0.152 | 0.026 | 0.113 | 0.118 | 0.148 | 0.198 | 0.214 | 0 | 0.072 | 0.035 | 0.029 | 0.031 | 0.065 | 0.139 | 0.153 | 0 |
| R ContC Cingp 1 | 0.148 | 0.023 | 0.112 | 0.116 | 0.145 | 0.192 | 0.203 | 0 | 0.107 | 0.042 | 0.038 | 0.044 | 0.103 | 0.183 | 0.2 | 0 |
| R DefaultA IPL 1 | 0.146 | 0.022 | 0.112 | 0.117 | 0.143 | 0.186 | 0.196 | 0.042 | 0.063 | 0.03 | 0.028 | 0.029 | 0.056 | 0.121 | 0.139 | 0.086 |
| R DefaultA PFCd 1 | 0.142 | 0.02 | 0.11 | 0.114 | 0.139 | 0.18 | 0.188 | 0.036 | 0.073 | 0.033 | 0.029 | 0.031 | 0.067 | 0.135 | 0.148 | 0 |
| R DefaultA pCunPCC 1 | 0.147 | 0.023 | 0.112 | 0.117 | 0.143 | 0.19 | 0.203 | 0 | 0.13 | 0.045 | 0.05 | 0.058 | 0.128 | 0.208 | 0.224 | 0.121 |
| R DefaultA PFCm 1 | 0.144 | 0.021 | 0.111 | 0.115 | 0.141 | 0.182 | 0.191 | 0 | 0.074 | 0.034 | 0.03 | 0.032 | 0.068 | 0.142 | 0.156 | 0.128 |
| R DefaultA PFCm 2 | 0.144 | 0.021 | 0.111 | 0.115 | 0.141 | 0.184 | 0.192 | 0.07 | 0.077 | 0.035 | 0.029 | 0.032 | 0.07 | 0.143 | 0.159 | 0.116 |
| R DefaultA PFCm 3 | 0.142 | 0.02 | 0.11 | 0.115 | 0.139 | 0.179 | 0.188 | 0 | 0.068 | 0.032 | 0.028 | 0.03 | 0.061 | 0.131 | 0.148 | 0.143 |
| R DefaultB Temp 1 | 0.196 | 0.036 | 0.132 | 0.14 | 0.193 | 0.259 | 0.274 | 0.03 | 0.091 | 0.04 | 0.032 | 0.036 | 0.085 | 0.165 | 0.18 | 0 |
| R DefaultB AntTemp 1 | 0.157 | 0.028 | 0.115 | 0.119 | 0.153 | 0.208 | 0.224 | 0.155 | 0.072 | 0.033 | 0.029 | 0.031 | 0.066 | 0.133 | 0.149 | 0.027 |
| R DefaultB PFCd 1 | 0.154 | 0.026 | 0.114 | 0.119 | 0.151 | 0.203 | 0.216 | 0.056 | 0.12 | 0.045 | 0.04 | 0.049 | 0.117 | 0.2 | 0.216 | 0.157 |
| R DefaultB PFCv 1 | 0.159 | 0.028 | 0.116 | 0.12 | 0.155 | 0.211 | 0.223 | 0.112 | 0.259 | 0.047 | 0.167 | 0.179 | 0.259 | 0.336 | 0.349 | 0.278 |
| R DefaultC IPL 1 | 0.144 | 0.021 | 0.111 | 0.115 | 0.141 | 0.182 | 0.194 | 0.045 | 0.065 | 0.031 | 0.028 | 0.029 | 0.057 | 0.126 | 0.14 | 0 |
| R DefaultC Rsp 1 | 0.168 | 0.032 | 0.118 | 0.124 | 0.164 | 0.227 | 0.24 | 0.005 | 0.135 | 0.047 | 0.052 | 0.062 | 0.131 | 0.219 | 0.238 | 0 |
| R DefaultC PHC 1 | 0.158 | 0.028 | 0.115 | 0.12 | 0.154 | 0.209 | 0.221 | 0.337 | 0.071 | 0.033 | 0.029 | 0.03 | 0.064 | 0.134 | 0.153 | 0.079 |
| R TempPar 1 | 0.142 | 0.02 | 0.112 | 0.115 | 0.14 | 0.18 | 0.188 | 0.111 | 0.104 | 0.044 | 0.034 | 0.039 | 0.1 | 0.182 | 0.199 | 0.046 |
| R TempPar 2 | 0.148 | 0.023 | 0.111 | 0.116 | 0.144 | 0.189 | 0.2 | 0 | 0.076 | 0.035 | 0.029 | 0.032 | 0.07 | 0.142 | 0.159 | 0.108 |
| R TempPar 3 | 0.156 | 0.027 | 0.113 | 0.119 | 0.151 | 0.205 | 0.22 | 0.22 | 0.08 | 0.038 | 0.03 | 0.032 | 0.072 | 0.151 | 0.169 | 0.024 |
| R TempPar 4 | 0.143 | 0.021 | 0.11 | 0.115 | 0.14 | 0.182 | 0.191 | 0.105 | 0.07 | 0.033 | 0.028 | 0.03 | 0.063 | 0.133 | 0.148 | 0 |
| L Thalamus | 0.145 | 0.021 | 0.111 | 0.115 | 0.141 | 0.184 | 0.195 | 0 | 0.073 | 0.033 | 0.03 | 0.032 | 0.067 | 0.135 | 0.149 | 0 |
| L Caudate | 0.163 | 0.03 | 0.117 | 0.122 | 0.158 | 0.218 | 0.229 | 0.13 | 0.178 | 0.05 | 0.089 | 0.1 | 0.176 | 0.261 | 0.279 | 0 |
| L Putamen | 0.153 | 0.026 | 0.115 | 0.118 | 0.149 | 0.201 | 0.217 | 0.168 | 0.105 | 0.046 | 0.035 | 0.04 | 0.099 | 0.188 | 0.206 | 0 |
| L Pallidum | 0.147 | 0.023 | 0.111 | 0.115 | 0.142 | 0.19 | 0.199 | 0.147 | 0.09 | 0.039 | 0.032 | 0.035 | 0.085 | 0.164 | 0.179 | 0.256 |
| L Hippocampus | 0.15 | 0.025 | 0.113 | 0.118 | 0.146 | 0.195 | 0.207 | 0.219 | 0.068 | 0.031 | 0.029 | 0.031 | 0.06 | 0.128 | 0.143 | 0.105 |
| L Amygdala | 0.168 | 0.031 | 0.119 | 0.124 | 0.165 | 0.224 | 0.237 | 0.118 | 0.157 | 0.046 | 0.075 | 0.085 | 0.155 | 0.236 | 0.25 | 0.217 |
| L Accumbens | 0.167 | 0.033 | 0.114 | 0.121 | 0.163 | 0.228 | 0.241 | 0.03 | 0.126 | 0.046 | 0.043 | 0.052 | 0.123 | 0.203 | 0.219 | 0.191 |
| R Thalamus | 0.143 | 0.02 | 0.111 | 0.115 | 0.14 | 0.18 | 0.189 | 0 | 0.063 | 0.03 | 0.028 | 0.029 | 0.055 | 0.12 | 0.138 | 0 |
| R Caudate | 0.146 | 0.023 | 0.111 | 0.115 | 0.142 | 0.189 | 0.2 | 0.025 | 0.223 | 0.047 | 0.131 | 0.146 | 0.223 | 0.299 | 0.318 | 0.006 |
| R Putamen | 0.143 | 0.02 | 0.11 | 0.115 | 0.139 | 0.18 | 0.188 | 0 | 0.105 | 0.045 | 0.033 | 0.039 | 0.1 | 0.187 | 0.207 | 0 |
| R Pallidum | 0.142 | 0.02 | 0.11 | 0.114 | 0.139 | 0.179 | 0.188 | 0.052 | 0.091 | 0.04 | 0.031 | 0.035 | 0.086 | 0.163 | 0.185 | 0 |
| R Hippocampus | 0.145 | 0.021 | 0.111 | 0.116 | 0.141 | 0.183 | 0.193 | 0.255 | 0.062 | 0.03 | 0.028 | 0.029 | 0.054 | 0.119 | 0.135 | 0.006 |
| R Amygdala | 0.166 | 0.031 | 0.118 | 0.124 | 0.161 | 0.222 | 0.234 | 0.102 | 0.117 | 0.044 | 0.04 | 0.048 | 0.113 | 0.194 | 0.211 | 0.176 |
| R Accumbens | 0.156 | 0.027 | 0.115 | 0.12 | 0.152 | 0.203 | 0.216 | 0 | 0.119 | 0.047 | 0.041 | 0.048 | 0.117 | 0.203 | 0.218 | 0.052 |

*Table S13.* ROI-level reliability for the visual search tSNR and task vs. baseline: Bayesian multilevel model (BMM) and conventional linear mixed-effects model (ICC) for 200 cortical parcels and 14 subcortical ROIs. (ICC: blue=<.4, red=.4-.6, orange=.6-.75, yellow=.75-1)

|  | **tSNR** | | | | | | | | **Task vs. Baseline** | | | | | | | |
| --- | --- | --- | --- | --- | --- | --- | --- | --- | --- | --- | --- | --- | --- | --- | --- | --- |
| **Label** | **BMM** | **SD** | **2.50%** | **5%** | **50%** | **95%** | **97.50%** | **ICC** | **BMM** | **SD** | **2.50%** | **5%** | **50%** | **95%** | **97.50%** | **ICC** |
| L VisCent ExStr 1 | 0.677 | 0.021 | 0.633 | 0.64 | 0.679 | 0.71 | 0.715 | 0.721 | 0.64 | 0.022 | 0.598 | 0.605 | 0.64 | 0.675 | 0.681 | 0.362 |
| L VisCent ExStr 2 | 0.762 | 0.016 | 0.728 | 0.733 | 0.763 | 0.785 | 0.79 | 0.702 | 0.642 | 0.021 | 0.601 | 0.607 | 0.643 | 0.676 | 0.683 | 0.406 |
| L VisCent Striate 1 | 0.737 | 0.017 | 0.701 | 0.707 | 0.739 | 0.764 | 0.767 | 0.759 | 0.693 | 0.017 | 0.658 | 0.663 | 0.694 | 0.721 | 0.726 | 0.405 |
| L VisCent ExStr 3 | 0.763 | 0.016 | 0.73 | 0.736 | 0.764 | 0.787 | 0.791 | 0.633 | 0.601 | 0.022 | 0.558 | 0.564 | 0.602 | 0.637 | 0.643 | 0.38 |
| L VisCent ExStr 4 | 0.718 | 0.019 | 0.677 | 0.685 | 0.719 | 0.746 | 0.751 | 0.546 | 0.541 | 0.025 | 0.491 | 0.498 | 0.541 | 0.582 | 0.589 | 0.448 |
| L VisCent ExStr 5 | 0.723 | 0.018 | 0.684 | 0.692 | 0.724 | 0.752 | 0.756 | 0.581 | 0.554 | 0.024 | 0.506 | 0.514 | 0.554 | 0.593 | 0.598 | 0.5 |
| L VisPeri ExStrInf 1 | 0.647 | 0.023 | 0.6 | 0.608 | 0.649 | 0.683 | 0.688 | 0.659 | 0.497 | 0.024 | 0.45 | 0.457 | 0.497 | 0.537 | 0.545 | 0.458 |
| L VisPeri ExStrInf 2 | 0.643 | 0.023 | 0.593 | 0.602 | 0.644 | 0.679 | 0.684 | 0.678 | 0.535 | 0.024 | 0.489 | 0.495 | 0.536 | 0.572 | 0.58 | 0.344 |
| L VisPeri ExStrInf 3 | 0.661 | 0.022 | 0.613 | 0.622 | 0.662 | 0.696 | 0.701 | 0.781 | 0.478 | 0.024 | 0.431 | 0.438 | 0.477 | 0.519 | 0.527 | 0.111 |
| L VisPeri StriCal 1 | 0.668 | 0.022 | 0.622 | 0.63 | 0.67 | 0.701 | 0.707 | 0.748 | 0.596 | 0.021 | 0.552 | 0.56 | 0.595 | 0.63 | 0.637 | 0.383 |
| L VisPeri ExStrSup 1 | 0.718 | 0.019 | 0.679 | 0.686 | 0.718 | 0.746 | 0.751 | 0.729 | 0.461 | 0.024 | 0.413 | 0.422 | 0.46 | 0.499 | 0.506 | 0.568 |
| L VisPeri ExStrSup 2 | 0.785 | 0.014 | 0.755 | 0.759 | 0.785 | 0.807 | 0.81 | 0.718 | 0.583 | 0.022 | 0.537 | 0.544 | 0.585 | 0.618 | 0.624 | 0.559 |
| L SomMotA 1 | 0.63 | 0.024 | 0.579 | 0.587 | 0.631 | 0.668 | 0.673 | 0.61 | 0.398 | 0.019 | 0.363 | 0.368 | 0.397 | 0.43 | 0.437 | 0.205 |
| L SomMotA 2 | 0.702 | 0.02 | 0.66 | 0.669 | 0.703 | 0.732 | 0.737 | 0.563 | 0.474 | 0.025 | 0.426 | 0.434 | 0.473 | 0.515 | 0.525 | 0.262 |
| L SomMotA 3 | 0.657 | 0.023 | 0.61 | 0.617 | 0.658 | 0.691 | 0.697 | 0.62 | 0.532 | 0.024 | 0.485 | 0.494 | 0.533 | 0.571 | 0.578 | 0.304 |
| L SomMotA 4 | 0.663 | 0.022 | 0.613 | 0.624 | 0.664 | 0.697 | 0.703 | 0.561 | 0.428 | 0.023 | 0.384 | 0.391 | 0.427 | 0.466 | 0.474 | 0.197 |
| L SomMotA 5 | 0.73 | 0.018 | 0.693 | 0.699 | 0.731 | 0.757 | 0.763 | 0.71 | 0.395 | 0.018 | 0.362 | 0.366 | 0.394 | 0.424 | 0.43 | 0.051 |
| L SomMotA 6 | 0.667 | 0.022 | 0.62 | 0.629 | 0.668 | 0.701 | 0.706 | 0.555 | 0.399 | 0.019 | 0.365 | 0.369 | 0.398 | 0.434 | 0.439 | 0.226 |
| L SomMotA 7 | 0.674 | 0.022 | 0.627 | 0.637 | 0.675 | 0.707 | 0.713 | 0.55 | 0.394 | 0.018 | 0.36 | 0.365 | 0.393 | 0.424 | 0.43 | 0.11 |
| L SomMotA 8 | 0.684 | 0.021 | 0.638 | 0.648 | 0.685 | 0.717 | 0.721 | 0.665 | 0.395 | 0.018 | 0.362 | 0.367 | 0.395 | 0.426 | 0.433 | 0.119 |
| L SomMotB Aud 1 | 0.677 | 0.021 | 0.632 | 0.64 | 0.678 | 0.709 | 0.714 | 0.69 | 0.401 | 0.02 | 0.362 | 0.369 | 0.4 | 0.437 | 0.444 | 0.117 |
| L SomMotB Aud 2 | 0.675 | 0.022 | 0.629 | 0.637 | 0.677 | 0.709 | 0.713 | 0.73 | 0.393 | 0.018 | 0.361 | 0.365 | 0.392 | 0.423 | 0.428 | 0.245 |
| L SomMotB S2 1 | 0.643 | 0.023 | 0.594 | 0.603 | 0.644 | 0.678 | 0.684 | 0.653 | 0.391 | 0.017 | 0.359 | 0.364 | 0.39 | 0.42 | 0.426 | 0.294 |
| L SomMotB S2 2 | 0.666 | 0.022 | 0.619 | 0.627 | 0.668 | 0.7 | 0.706 | 0.763 | 0.421 | 0.023 | 0.375 | 0.383 | 0.421 | 0.459 | 0.467 | 0.349 |
| L SomMotB Aud 3 | 0.678 | 0.021 | 0.631 | 0.639 | 0.679 | 0.709 | 0.714 | 0.698 | 0.397 | 0.019 | 0.361 | 0.367 | 0.396 | 0.429 | 0.437 | 0.388 |
| L SomMotB S2 3 | 0.69 | 0.02 | 0.648 | 0.655 | 0.691 | 0.721 | 0.726 | 0.714 | 0.405 | 0.022 | 0.367 | 0.371 | 0.403 | 0.441 | 0.449 | 0.254 |
| L SomMotB Cent 1 | 0.695 | 0.02 | 0.651 | 0.659 | 0.696 | 0.726 | 0.731 | 0.764 | 0.425 | 0.024 | 0.38 | 0.386 | 0.425 | 0.465 | 0.472 | 0.325 |
| L SomMotB Cent 2 | 0.678 | 0.021 | 0.63 | 0.641 | 0.679 | 0.71 | 0.716 | 0.734 | 0.402 | 0.021 | 0.364 | 0.37 | 0.402 | 0.437 | 0.445 | 0.288 |
| L DorsAttnA TempOcc 1 | 0.682 | 0.021 | 0.636 | 0.644 | 0.683 | 0.713 | 0.719 | 0.773 | 0.524 | 0.024 | 0.476 | 0.484 | 0.524 | 0.562 | 0.569 | 0.557 |
| L DorsAttnA TempOcc 2 | 0.689 | 0.021 | 0.644 | 0.652 | 0.69 | 0.72 | 0.725 | 0.692 | 0.563 | 0.023 | 0.516 | 0.524 | 0.562 | 0.599 | 0.607 | 0.535 |
| L DorsAttnA ParOcc 1 | 0.703 | 0.02 | 0.662 | 0.669 | 0.705 | 0.732 | 0.738 | 0.668 | 0.421 | 0.023 | 0.376 | 0.383 | 0.42 | 0.461 | 0.467 | 0.201 |
| L DorsAttnA SPL 1 | 0.689 | 0.02 | 0.646 | 0.652 | 0.69 | 0.719 | 0.725 | 0.598 | 0.511 | 0.025 | 0.462 | 0.468 | 0.511 | 0.552 | 0.561 | 0.547 |
| L DorsAttnA SPL 2 | 0.733 | 0.018 | 0.695 | 0.703 | 0.735 | 0.76 | 0.764 | 0.639 | 0.543 | 0.024 | 0.496 | 0.502 | 0.544 | 0.583 | 0.589 | 0.379 |
| L DorsAttnA SPL 3 | 0.732 | 0.018 | 0.694 | 0.7 | 0.734 | 0.76 | 0.764 | 0.575 | 0.548 | 0.025 | 0.498 | 0.506 | 0.548 | 0.587 | 0.594 | 0.313 |
| L DorsAttnB PostC 1 | 0.692 | 0.02 | 0.647 | 0.656 | 0.693 | 0.722 | 0.727 | 0.652 | 0.432 | 0.023 | 0.386 | 0.392 | 0.432 | 0.471 | 0.476 | 0.448 |
| L DorsAttnB PostC 2 | 0.651 | 0.023 | 0.603 | 0.611 | 0.653 | 0.687 | 0.692 | 0.59 | 0.455 | 0.023 | 0.411 | 0.418 | 0.455 | 0.493 | 0.499 | 0.298 |
| L DorsAttnB PostC 3 | 0.711 | 0.019 | 0.67 | 0.677 | 0.713 | 0.74 | 0.746 | 0.512 | 0.427 | 0.023 | 0.383 | 0.39 | 0.428 | 0.465 | 0.471 | 0.223 |
| L DorsAttnB PostC 4 | 0.736 | 0.018 | 0.699 | 0.706 | 0.737 | 0.763 | 0.768 | 0.532 | 0.404 | 0.02 | 0.366 | 0.371 | 0.403 | 0.438 | 0.446 | 0.035 |
| L DorsAttnB FEF 1 | 0.682 | 0.021 | 0.635 | 0.645 | 0.683 | 0.714 | 0.719 | 0.712 | 0.42 | 0.023 | 0.376 | 0.382 | 0.42 | 0.459 | 0.465 | 0.248 |
| L SalVentAttnA ParOper 1 | 0.692 | 0.02 | 0.65 | 0.657 | 0.694 | 0.723 | 0.728 | 0.736 | 0.39 | 0.016 | 0.36 | 0.363 | 0.391 | 0.418 | 0.422 | 0.173 |
| L SalVentAttnA Ins 1 | 0.65 | 0.023 | 0.601 | 0.609 | 0.652 | 0.686 | 0.692 | 0.697 | 0.39 | 0.016 | 0.359 | 0.363 | 0.389 | 0.416 | 0.421 | 0.385 |
| L SalVentAttnA FrOper 1 | 0.678 | 0.021 | 0.633 | 0.641 | 0.68 | 0.711 | 0.716 | 0.677 | 0.413 | 0.023 | 0.372 | 0.377 | 0.413 | 0.452 | 0.459 | 0.434 |
| L SalVentAttnA FrOper 2 | 0.698 | 0.02 | 0.655 | 0.663 | 0.699 | 0.728 | 0.733 | 0.745 | 0.449 | 0.024 | 0.404 | 0.41 | 0.449 | 0.489 | 0.497 | 0.617 |
| L SalVentAttnA ParMed 1 | 0.655 | 0.023 | 0.605 | 0.615 | 0.657 | 0.69 | 0.695 | 0.694 | 0.391 | 0.017 | 0.361 | 0.364 | 0.391 | 0.42 | 0.426 | 0.226 |
| L SalVentAttnA FrMed 1 | 0.646 | 0.023 | 0.598 | 0.606 | 0.648 | 0.682 | 0.687 | 0.534 | 0.404 | 0.021 | 0.367 | 0.372 | 0.402 | 0.44 | 0.448 | 0.114 |
| L SalVentAttnA FrMed 2 | 0.682 | 0.021 | 0.638 | 0.646 | 0.683 | 0.713 | 0.718 | 0.705 | 0.453 | 0.025 | 0.406 | 0.413 | 0.453 | 0.494 | 0.504 | 0.218 |
| L SalVentAttnB IPL 1 | 0.708 | 0.019 | 0.667 | 0.674 | 0.709 | 0.737 | 0.741 | 0.609 | 0.414 | 0.022 | 0.371 | 0.377 | 0.413 | 0.45 | 0.457 | 0.35 |
| L SalVentAttnB PFCl 1 | 0.674 | 0.022 | 0.629 | 0.636 | 0.676 | 0.706 | 0.712 | 0.706 | 0.389 | 0.016 | 0.358 | 0.362 | 0.389 | 0.416 | 0.421 | 0.261 |
| L SalVentAttnB Ins 1 | 0.642 | 0.024 | 0.593 | 0.601 | 0.643 | 0.678 | 0.683 | 0.636 | 0.394 | 0.018 | 0.361 | 0.366 | 0.394 | 0.423 | 0.43 | 0.124 |
| L SalVentAttnB PFCmp 1 | 0.664 | 0.022 | 0.617 | 0.626 | 0.665 | 0.698 | 0.704 | 0.544 | 0.417 | 0.022 | 0.375 | 0.381 | 0.416 | 0.456 | 0.462 | 0.296 |
| L LimbicB OFC 1 | 0.667 | 0.022 | 0.622 | 0.629 | 0.668 | 0.701 | 0.707 | 0.664 | 0.4 | 0.019 | 0.365 | 0.37 | 0.399 | 0.433 | 0.439 | 0 |
| L LimbicB OFC 2 | 0.755 | 0.017 | 0.719 | 0.726 | 0.756 | 0.78 | 0.785 | 0.513 | 0.461 | 0.023 | 0.417 | 0.423 | 0.462 | 0.5 | 0.507 | 0.116 |
| L LimbicA TempPole 1 | 0.699 | 0.02 | 0.658 | 0.665 | 0.7 | 0.73 | 0.734 | 0.61 | 0.469 | 0.024 | 0.423 | 0.429 | 0.47 | 0.51 | 0.517 | 0.035 |
| L LimbicA TempPole 2 | 0.702 | 0.02 | 0.66 | 0.668 | 0.704 | 0.733 | 0.737 | 0.531 | 0.401 | 0.021 | 0.364 | 0.369 | 0.399 | 0.437 | 0.444 | 0 |
| L LimbicA TempPole 3 | 0.669 | 0.022 | 0.623 | 0.631 | 0.671 | 0.702 | 0.708 | 0.756 | 0.509 | 0.025 | 0.46 | 0.469 | 0.509 | 0.548 | 0.557 | 0 |
| L LimbicA TempPole 4 | 0.674 | 0.021 | 0.628 | 0.637 | 0.675 | 0.706 | 0.711 | 0.574 | 0.56 | 0.024 | 0.513 | 0.52 | 0.561 | 0.599 | 0.605 | 0.052 |
| L ContA Temp 1 | 0.7 | 0.02 | 0.658 | 0.666 | 0.702 | 0.731 | 0.735 | 0.696 | 0.5 | 0.024 | 0.453 | 0.46 | 0.5 | 0.541 | 0.547 | 0.466 |
| L ContA IPS 1 | 0.695 | 0.02 | 0.653 | 0.66 | 0.696 | 0.726 | 0.731 | 0.628 | 0.424 | 0.023 | 0.38 | 0.386 | 0.424 | 0.465 | 0.471 | 0.154 |
| L ContA IPS 2 | 0.689 | 0.021 | 0.645 | 0.653 | 0.69 | 0.721 | 0.725 | 0.562 | 0.452 | 0.024 | 0.406 | 0.411 | 0.452 | 0.493 | 0.499 | 0.355 |
| L ContA IPS 3 | 0.682 | 0.021 | 0.637 | 0.644 | 0.683 | 0.713 | 0.717 | 0.576 | 0.448 | 0.023 | 0.404 | 0.412 | 0.448 | 0.488 | 0.496 | 0.303 |
| L ContA PFCd 1 | 0.663 | 0.022 | 0.615 | 0.623 | 0.665 | 0.698 | 0.705 | 0.567 | 0.402 | 0.02 | 0.365 | 0.37 | 0.402 | 0.435 | 0.442 | 0.118 |
| L ContA PFClv 1 | 0.653 | 0.023 | 0.606 | 0.614 | 0.655 | 0.689 | 0.694 | 0.678 | 0.391 | 0.017 | 0.36 | 0.364 | 0.391 | 0.42 | 0.426 | 0.281 |
| L ContA PFCl 1 | 0.667 | 0.022 | 0.62 | 0.628 | 0.668 | 0.701 | 0.706 | 0.673 | 0.408 | 0.022 | 0.367 | 0.374 | 0.408 | 0.445 | 0.452 | 0.017 |
| L ContA PFCl 2 | 0.681 | 0.021 | 0.636 | 0.644 | 0.682 | 0.712 | 0.718 | 0.787 | 0.519 | 0.024 | 0.473 | 0.48 | 0.519 | 0.558 | 0.565 | 0.435 |
| L ContA PFCl 3 | 0.682 | 0.021 | 0.638 | 0.646 | 0.684 | 0.715 | 0.72 | 0.738 | 0.42 | 0.023 | 0.376 | 0.382 | 0.42 | 0.458 | 0.465 | 0.356 |
| L ContA Cingm 1 | 0.721 | 0.018 | 0.682 | 0.69 | 0.722 | 0.749 | 0.754 | 0.444 | 0.444 | 0.023 | 0.398 | 0.406 | 0.444 | 0.483 | 0.491 | 0.13 |
| L ContB Temp 1 | 0.716 | 0.019 | 0.676 | 0.683 | 0.718 | 0.745 | 0.749 | 0.653 | 0.391 | 0.017 | 0.359 | 0.364 | 0.391 | 0.419 | 0.424 | 0.042 |
| L ContB IPL 1 | 0.697 | 0.02 | 0.654 | 0.662 | 0.698 | 0.729 | 0.734 | 0.544 | 0.395 | 0.018 | 0.361 | 0.366 | 0.394 | 0.426 | 0.432 | 0.273 |
| L ContB PFCl 1 | 0.682 | 0.021 | 0.638 | 0.646 | 0.684 | 0.714 | 0.719 | 0.728 | 0.392 | 0.017 | 0.36 | 0.364 | 0.392 | 0.421 | 0.426 | 0.218 |
| L ContB PFClv 1 | 0.701 | 0.02 | 0.658 | 0.667 | 0.703 | 0.731 | 0.736 | 0.493 | 0.426 | 0.023 | 0.38 | 0.388 | 0.426 | 0.465 | 0.473 | 0.12 |
| L ContB PFClv 2 | 0.702 | 0.02 | 0.661 | 0.669 | 0.703 | 0.732 | 0.736 | 0.624 | 0.396 | 0.019 | 0.362 | 0.368 | 0.395 | 0.429 | 0.436 | 0.319 |
| L ContC pCun 1 | 0.682 | 0.021 | 0.637 | 0.645 | 0.684 | 0.714 | 0.719 | 0.696 | 0.456 | 0.023 | 0.409 | 0.417 | 0.456 | 0.492 | 0.499 | 0.246 |
| L ContC pCun 2 | 0.706 | 0.02 | 0.664 | 0.672 | 0.707 | 0.736 | 0.74 | 0.692 | 0.439 | 0.024 | 0.394 | 0.4 | 0.438 | 0.478 | 0.484 | 0.122 |
| L ContC Cingp 1 | 0.646 | 0.024 | 0.594 | 0.604 | 0.647 | 0.682 | 0.688 | 0.742 | 0.394 | 0.018 | 0.361 | 0.366 | 0.393 | 0.424 | 0.43 | 0 |
| L DefaultA IPL 1 | 0.685 | 0.021 | 0.642 | 0.648 | 0.686 | 0.717 | 0.722 | 0.589 | 0.439 | 0.024 | 0.393 | 0.399 | 0.439 | 0.479 | 0.486 | 0.388 |
| L DefaultA PFCd 1 | 0.71 | 0.019 | 0.671 | 0.678 | 0.711 | 0.74 | 0.744 | 0.755 | 0.391 | 0.017 | 0.361 | 0.365 | 0.391 | 0.419 | 0.425 | 0.084 |
| L DefaultA pCunPCC 1 | 0.656 | 0.023 | 0.606 | 0.616 | 0.657 | 0.691 | 0.696 | 0.663 | 0.449 | 0.023 | 0.405 | 0.411 | 0.449 | 0.488 | 0.495 | 0.05 |
| L DefaultA pCunPCC 2 | 0.654 | 0.023 | 0.606 | 0.614 | 0.656 | 0.689 | 0.694 | 0.721 | 0.428 | 0.023 | 0.386 | 0.391 | 0.428 | 0.468 | 0.476 | 0.008 |
| L DefaultA pCunPCC 3 | 0.659 | 0.023 | 0.61 | 0.619 | 0.66 | 0.693 | 0.699 | 0.689 | 0.418 | 0.023 | 0.374 | 0.379 | 0.418 | 0.456 | 0.462 | 0.155 |
| L DefaultA PFCm 1 | 0.683 | 0.021 | 0.638 | 0.646 | 0.684 | 0.715 | 0.719 | 0.646 | 0.515 | 0.025 | 0.466 | 0.473 | 0.514 | 0.556 | 0.563 | 0.183 |
| L DefaultA PFCm 2 | 0.659 | 0.023 | 0.609 | 0.618 | 0.66 | 0.695 | 0.7 | 0.576 | 0.428 | 0.023 | 0.383 | 0.391 | 0.428 | 0.466 | 0.474 | 0.31 |
| L DefaultA PFCm 3 | 0.661 | 0.022 | 0.613 | 0.621 | 0.662 | 0.695 | 0.701 | 0.639 | 0.416 | 0.023 | 0.371 | 0.378 | 0.415 | 0.452 | 0.459 | 0.136 |
| L DefaultB Temp 1 | 0.692 | 0.021 | 0.649 | 0.655 | 0.693 | 0.724 | 0.729 | 0.683 | 0.399 | 0.019 | 0.364 | 0.368 | 0.398 | 0.433 | 0.44 | 0.381 |
| L DefaultB Temp 2 | 0.744 | 0.017 | 0.707 | 0.714 | 0.745 | 0.77 | 0.774 | 0.702 | 0.41 | 0.022 | 0.368 | 0.375 | 0.41 | 0.448 | 0.456 | 0.274 |
| L DefaultB Temp 3 | 0.663 | 0.022 | 0.616 | 0.625 | 0.665 | 0.697 | 0.703 | 0.588 | 0.392 | 0.017 | 0.36 | 0.364 | 0.391 | 0.421 | 0.426 | 0.198 |
| L DefaultB Temp 4 | 0.676 | 0.022 | 0.629 | 0.638 | 0.677 | 0.709 | 0.713 | 0.714 | 0.416 | 0.023 | 0.375 | 0.38 | 0.415 | 0.454 | 0.463 | 0.335 |
| L DefaultB IPL 1 | 0.71 | 0.019 | 0.671 | 0.677 | 0.711 | 0.739 | 0.744 | 0.687 | 0.395 | 0.018 | 0.362 | 0.367 | 0.394 | 0.426 | 0.432 | 0.221 |
| L DefaultB PFCd 1 | 0.657 | 0.023 | 0.609 | 0.619 | 0.658 | 0.691 | 0.698 | 0.521 | 0.461 | 0.026 | 0.412 | 0.418 | 0.461 | 0.502 | 0.51 | 0.37 |
| L DefaultB PFCd 2 | 0.748 | 0.017 | 0.712 | 0.719 | 0.749 | 0.774 | 0.776 | 0.799 | 0.396 | 0.019 | 0.362 | 0.367 | 0.395 | 0.428 | 0.436 | 0.194 |
| L DefaultB PFCd 3 | 0.665 | 0.022 | 0.618 | 0.628 | 0.666 | 0.699 | 0.704 | 0.707 | 0.395 | 0.018 | 0.361 | 0.366 | 0.394 | 0.427 | 0.433 | 0.127 |
| L DefaultB PFCd 4 | 0.7 | 0.02 | 0.658 | 0.666 | 0.702 | 0.731 | 0.735 | 0.7 | 0.393 | 0.017 | 0.36 | 0.364 | 0.392 | 0.421 | 0.429 | 0.165 |
| L DefaultB PFCv 1 | 0.655 | 0.023 | 0.605 | 0.614 | 0.656 | 0.69 | 0.695 | 0.694 | 0.393 | 0.017 | 0.361 | 0.366 | 0.392 | 0.423 | 0.43 | 0.277 |
| L DefaultB PFCv 2 | 0.668 | 0.022 | 0.621 | 0.63 | 0.669 | 0.702 | 0.707 | 0.594 | 0.396 | 0.018 | 0.363 | 0.367 | 0.395 | 0.427 | 0.434 | 0.122 |
| L DefaultB PFCv 3 | 0.694 | 0.02 | 0.652 | 0.66 | 0.696 | 0.724 | 0.731 | 0.836 | 0.393 | 0.018 | 0.361 | 0.364 | 0.393 | 0.425 | 0.431 | 0.181 |
| L DefaultB PFCv 4 | 0.669 | 0.022 | 0.621 | 0.631 | 0.671 | 0.703 | 0.708 | 0.781 | 0.395 | 0.018 | 0.362 | 0.366 | 0.395 | 0.427 | 0.434 | 0.297 |
| L DefaultC IPL 1 | 0.712 | 0.019 | 0.672 | 0.678 | 0.713 | 0.741 | 0.745 | 0.638 | 0.513 | 0.024 | 0.464 | 0.473 | 0.514 | 0.551 | 0.558 | 0.507 |
| L DefaultC Rsp 1 | 0.648 | 0.023 | 0.6 | 0.607 | 0.649 | 0.684 | 0.69 | 0.734 | 0.462 | 0.025 | 0.415 | 0.422 | 0.462 | 0.503 | 0.511 | 0.171 |
| L DefaultC PHC 1 | 0.63 | 0.024 | 0.579 | 0.587 | 0.631 | 0.667 | 0.674 | 0.785 | 0.427 | 0.023 | 0.384 | 0.39 | 0.426 | 0.465 | 0.473 | 0 |
| L TempPar 1 | 0.68 | 0.021 | 0.636 | 0.644 | 0.681 | 0.711 | 0.717 | 0.689 | 0.402 | 0.02 | 0.365 | 0.37 | 0.401 | 0.438 | 0.445 | 0.392 |
| L TempPar 2 | 0.698 | 0.02 | 0.655 | 0.662 | 0.699 | 0.728 | 0.733 | 0.712 | 0.45 | 0.024 | 0.402 | 0.411 | 0.45 | 0.49 | 0.496 | 0.478 |
| R VisCent ExStr 1 | 0.663 | 0.022 | 0.616 | 0.625 | 0.665 | 0.697 | 0.703 | 0.595 | 0.586 | 0.022 | 0.541 | 0.548 | 0.587 | 0.623 | 0.628 | 0.442 |
| R VisCent ExStr 2 | 0.729 | 0.018 | 0.691 | 0.698 | 0.731 | 0.757 | 0.761 | 0.657 | 0.634 | 0.019 | 0.595 | 0.601 | 0.635 | 0.664 | 0.67 | 0.48 |
| R VisCent Striate 1 | 0.698 | 0.02 | 0.657 | 0.663 | 0.7 | 0.728 | 0.733 | 0.639 | 0.608 | 0.023 | 0.562 | 0.57 | 0.609 | 0.644 | 0.651 | 0.183 |
| R VisCent ExStr 3 | 0.753 | 0.016 | 0.719 | 0.725 | 0.754 | 0.778 | 0.781 | 0.647 | 0.673 | 0.018 | 0.636 | 0.642 | 0.674 | 0.702 | 0.708 | 0.399 |
| R VisCent ExStr 4 | 0.695 | 0.021 | 0.651 | 0.66 | 0.696 | 0.727 | 0.731 | 0.367 | 0.604 | 0.021 | 0.56 | 0.569 | 0.605 | 0.638 | 0.643 | 0.541 |
| R VisCent ExStr 5 | 0.755 | 0.017 | 0.721 | 0.727 | 0.756 | 0.78 | 0.785 | 0.66 | 0.593 | 0.022 | 0.548 | 0.556 | 0.594 | 0.629 | 0.635 | 0.407 |
| R VisPeri ExStrInf 1 | 0.653 | 0.023 | 0.604 | 0.611 | 0.654 | 0.689 | 0.694 | 0.682 | 0.461 | 0.024 | 0.416 | 0.423 | 0.461 | 0.501 | 0.507 | 0.34 |
| R VisPeri ExStrInf 2 | 0.64 | 0.023 | 0.591 | 0.599 | 0.642 | 0.677 | 0.682 | 0.719 | 0.459 | 0.024 | 0.412 | 0.421 | 0.459 | 0.5 | 0.507 | 0.15 |
| R VisPeri StriCal 1 | 0.678 | 0.021 | 0.633 | 0.641 | 0.679 | 0.71 | 0.716 | 0.667 | 0.681 | 0.018 | 0.643 | 0.649 | 0.682 | 0.709 | 0.714 | 0.485 |
| R VisPeri ExStrSup 1 | 0.659 | 0.022 | 0.612 | 0.619 | 0.66 | 0.693 | 0.698 | 0.648 | 0.492 | 0.025 | 0.443 | 0.451 | 0.493 | 0.532 | 0.538 | 0.177 |
| R VisPeri ExStrSup 2 | 0.707 | 0.019 | 0.666 | 0.674 | 0.708 | 0.737 | 0.741 | 0.616 | 0.455 | 0.025 | 0.409 | 0.415 | 0.454 | 0.498 | 0.506 | 0.456 |
| R VisPeri ExStrSup 3 | 0.783 | 0.014 | 0.753 | 0.758 | 0.784 | 0.804 | 0.808 | 0.714 | 0.613 | 0.021 | 0.57 | 0.577 | 0.614 | 0.647 | 0.652 | 0.419 |
| R SomMotA 1 | 0.736 | 0.017 | 0.697 | 0.706 | 0.737 | 0.762 | 0.766 | 0.649 | 0.437 | 0.024 | 0.392 | 0.399 | 0.437 | 0.476 | 0.486 | 0.217 |
| R SomMotA 2 | 0.693 | 0.02 | 0.65 | 0.658 | 0.694 | 0.724 | 0.728 | 0.66 | 0.404 | 0.02 | 0.365 | 0.371 | 0.403 | 0.439 | 0.446 | 0.128 |
| R SomMotA 3 | 0.648 | 0.023 | 0.598 | 0.607 | 0.649 | 0.683 | 0.69 | 0.69 | 0.424 | 0.024 | 0.377 | 0.384 | 0.423 | 0.463 | 0.474 | 0.22 |
| R SomMotA 4 | 0.702 | 0.02 | 0.661 | 0.666 | 0.704 | 0.732 | 0.737 | 0.646 | 0.511 | 0.024 | 0.463 | 0.472 | 0.511 | 0.551 | 0.559 | 0.353 |
| R SomMotA 5 | 0.744 | 0.017 | 0.707 | 0.715 | 0.746 | 0.769 | 0.774 | 0.607 | 0.446 | 0.024 | 0.399 | 0.406 | 0.445 | 0.486 | 0.493 | 0.187 |
| R SomMotA 6 | 0.681 | 0.021 | 0.636 | 0.644 | 0.682 | 0.712 | 0.718 | 0.616 | 0.435 | 0.023 | 0.391 | 0.396 | 0.435 | 0.474 | 0.481 | 0.219 |
| R SomMotA 7 | 0.686 | 0.021 | 0.643 | 0.65 | 0.688 | 0.717 | 0.723 | 0.575 | 0.395 | 0.018 | 0.362 | 0.366 | 0.394 | 0.424 | 0.431 | 0.042 |
| R SomMotA 8 | 0.661 | 0.022 | 0.613 | 0.621 | 0.662 | 0.695 | 0.7 | 0.555 | 0.416 | 0.023 | 0.373 | 0.379 | 0.415 | 0.454 | 0.462 | 0.204 |
| R SomMotA 9 | 0.689 | 0.021 | 0.646 | 0.653 | 0.69 | 0.72 | 0.723 | 0.522 | 0.39 | 0.016 | 0.359 | 0.363 | 0.39 | 0.418 | 0.424 | 0 |
| R SomMotA 10 | 0.658 | 0.022 | 0.612 | 0.618 | 0.66 | 0.693 | 0.698 | 0.526 | 0.394 | 0.017 | 0.361 | 0.366 | 0.393 | 0.423 | 0.429 | 0.096 |
| R SomMotA 11 | 0.678 | 0.021 | 0.633 | 0.64 | 0.679 | 0.71 | 0.715 | 0.542 | 0.404 | 0.021 | 0.365 | 0.371 | 0.403 | 0.44 | 0.447 | 0.126 |
| R SomMotB Aud 1 | 0.675 | 0.021 | 0.63 | 0.638 | 0.677 | 0.708 | 0.712 | 0.683 | 0.416 | 0.022 | 0.374 | 0.379 | 0.415 | 0.454 | 0.459 | 0.358 |
| R SomMotB Aud 2 | 0.718 | 0.018 | 0.68 | 0.686 | 0.72 | 0.747 | 0.752 | 0.673 | 0.394 | 0.018 | 0.362 | 0.366 | 0.393 | 0.425 | 0.43 | 0.268 |
| R SomMotB S2 1 | 0.655 | 0.023 | 0.608 | 0.615 | 0.657 | 0.689 | 0.696 | 0.71 | 0.392 | 0.018 | 0.359 | 0.364 | 0.391 | 0.422 | 0.429 | 0.145 |
| R SomMotB S2 2 | 0.671 | 0.022 | 0.625 | 0.633 | 0.673 | 0.704 | 0.71 | 0.663 | 0.426 | 0.023 | 0.382 | 0.388 | 0.425 | 0.464 | 0.473 | 0.392 |
| R SomMotB S2 3 | 0.722 | 0.018 | 0.681 | 0.689 | 0.724 | 0.75 | 0.755 | 0.731 | 0.526 | 0.024 | 0.481 | 0.487 | 0.526 | 0.567 | 0.573 | 0.43 |
| R SomMotB S2 4 | 0.704 | 0.019 | 0.663 | 0.671 | 0.706 | 0.734 | 0.739 | 0.703 | 0.445 | 0.023 | 0.399 | 0.408 | 0.445 | 0.484 | 0.491 | 0.361 |
| R SomMotB Cent 1 | 0.697 | 0.02 | 0.654 | 0.662 | 0.698 | 0.727 | 0.732 | 0.703 | 0.452 | 0.024 | 0.403 | 0.411 | 0.451 | 0.492 | 0.501 | 0.334 |
| R DorsAttnA TempOcc 1 | 0.647 | 0.023 | 0.598 | 0.606 | 0.649 | 0.684 | 0.689 | 0.574 | 0.476 | 0.024 | 0.427 | 0.438 | 0.476 | 0.514 | 0.523 | 0.439 |
| R DorsAttnA ParOcc 1 | 0.676 | 0.022 | 0.629 | 0.637 | 0.677 | 0.709 | 0.714 | 0.525 | 0.503 | 0.025 | 0.455 | 0.463 | 0.503 | 0.544 | 0.55 | 0.6 |
| R DorsAttnA SPL 1 | 0.669 | 0.022 | 0.624 | 0.631 | 0.671 | 0.702 | 0.708 | 0.573 | 0.49 | 0.025 | 0.439 | 0.447 | 0.49 | 0.531 | 0.537 | 0.451 |
| R DorsAttnA SPL 2 | 0.714 | 0.019 | 0.673 | 0.682 | 0.716 | 0.744 | 0.748 | 0.648 | 0.546 | 0.023 | 0.501 | 0.508 | 0.546 | 0.582 | 0.589 | 0.426 |
| R DorsAttnA SPL 3 | 0.682 | 0.021 | 0.639 | 0.646 | 0.683 | 0.715 | 0.719 | 0.611 | 0.47 | 0.024 | 0.421 | 0.431 | 0.47 | 0.508 | 0.517 | 0.511 |
| R DorsAttnA SPL 4 | 0.71 | 0.02 | 0.669 | 0.676 | 0.711 | 0.739 | 0.744 | 0.582 | 0.538 | 0.024 | 0.491 | 0.498 | 0.538 | 0.577 | 0.582 | 0.502 |
| R DorsAttnB PostC 1 | 0.722 | 0.018 | 0.683 | 0.69 | 0.723 | 0.748 | 0.753 | 0.643 | 0.415 | 0.023 | 0.372 | 0.379 | 0.415 | 0.453 | 0.46 | 0.293 |
| R DorsAttnB PostC 2 | 0.677 | 0.021 | 0.632 | 0.641 | 0.679 | 0.709 | 0.715 | 0.596 | 0.446 | 0.024 | 0.4 | 0.408 | 0.446 | 0.485 | 0.493 | 0.39 |
| R DorsAttnB PostC 3 | 0.684 | 0.021 | 0.639 | 0.646 | 0.686 | 0.716 | 0.721 | 0.606 | 0.421 | 0.023 | 0.378 | 0.383 | 0.421 | 0.459 | 0.466 | 0.168 |
| R DorsAttnB PostC 4 | 0.739 | 0.017 | 0.702 | 0.71 | 0.74 | 0.765 | 0.77 | 0.606 | 0.4 | 0.02 | 0.364 | 0.369 | 0.399 | 0.434 | 0.44 | 0.011 |
| R DorsAttnB FEF 1 | 0.67 | 0.022 | 0.623 | 0.633 | 0.672 | 0.704 | 0.708 | 0.735 | 0.42 | 0.024 | 0.375 | 0.382 | 0.42 | 0.46 | 0.466 | 0.436 |
| R SalVentAttnA ParOper 1 | 0.671 | 0.022 | 0.628 | 0.634 | 0.673 | 0.705 | 0.71 | 0.631 | 0.403 | 0.02 | 0.366 | 0.371 | 0.402 | 0.438 | 0.447 | 0.292 |
| R SalVentAttnA PrC 1 | 0.684 | 0.021 | 0.64 | 0.647 | 0.686 | 0.716 | 0.721 | 0.669 | 0.52 | 0.024 | 0.474 | 0.481 | 0.521 | 0.557 | 0.564 | 0.588 |
| R SalVentAttnA Ins 1 | 0.691 | 0.021 | 0.648 | 0.655 | 0.692 | 0.722 | 0.727 | 0.701 | 0.405 | 0.021 | 0.366 | 0.372 | 0.404 | 0.441 | 0.449 | 0.022 |
| R SalVentAttnA Ins 2 | 0.656 | 0.023 | 0.606 | 0.618 | 0.658 | 0.692 | 0.696 | 0.684 | 0.394 | 0.018 | 0.361 | 0.366 | 0.393 | 0.426 | 0.432 | 0.188 |
| R SalVentAttnA FrOper 1 | 0.651 | 0.023 | 0.603 | 0.609 | 0.652 | 0.685 | 0.69 | 0.669 | 0.391 | 0.017 | 0.359 | 0.363 | 0.391 | 0.421 | 0.427 | 0.335 |
| R SalVentAttnA FrMed 1 | 0.643 | 0.024 | 0.591 | 0.603 | 0.644 | 0.678 | 0.686 | 0.531 | 0.399 | 0.02 | 0.363 | 0.367 | 0.399 | 0.434 | 0.439 | 0.253 |
| R SalVentAttnA ParMed 1 | 0.664 | 0.022 | 0.618 | 0.626 | 0.665 | 0.698 | 0.703 | 0.62 | 0.39 | 0.016 | 0.359 | 0.363 | 0.39 | 0.417 | 0.422 | 0.059 |
| R SalVentAttnA ParMed 2 | 0.65 | 0.023 | 0.599 | 0.608 | 0.65 | 0.685 | 0.691 | 0.677 | 0.404 | 0.021 | 0.366 | 0.371 | 0.403 | 0.438 | 0.446 | 0.176 |
| R SalVentAttnA FrMed 2 | 0.672 | 0.022 | 0.625 | 0.634 | 0.673 | 0.705 | 0.71 | 0.724 | 0.403 | 0.021 | 0.365 | 0.37 | 0.402 | 0.44 | 0.448 | 0.212 |
| R SalVentAttnB IPL 1 | 0.724 | 0.018 | 0.685 | 0.692 | 0.725 | 0.752 | 0.756 | 0.639 | 0.447 | 0.024 | 0.4 | 0.407 | 0.448 | 0.487 | 0.495 | 0.485 |
| R SalVentAttnB PFClv 1 | 0.685 | 0.021 | 0.64 | 0.648 | 0.685 | 0.717 | 0.723 | 0.664 | 0.471 | 0.024 | 0.424 | 0.432 | 0.471 | 0.512 | 0.519 | 0.462 |
| R SalVentAttnB PFCl 1 | 0.691 | 0.02 | 0.649 | 0.657 | 0.692 | 0.722 | 0.728 | 0.597 | 0.422 | 0.024 | 0.376 | 0.384 | 0.423 | 0.461 | 0.469 | 0.413 |
| R SalVentAttnB Ins 1 | 0.663 | 0.022 | 0.615 | 0.623 | 0.665 | 0.697 | 0.702 | 0.632 | 0.427 | 0.022 | 0.385 | 0.393 | 0.427 | 0.464 | 0.473 | 0.28 |
| R SalVentAttnB Ins 2 | 0.69 | 0.021 | 0.647 | 0.655 | 0.692 | 0.722 | 0.728 | 0.688 | 0.466 | 0.024 | 0.417 | 0.424 | 0.466 | 0.505 | 0.514 | 0.406 |
| R SalVentAttnB PFCmp 1 | 0.656 | 0.023 | 0.607 | 0.617 | 0.657 | 0.69 | 0.696 | 0.572 | 0.406 | 0.022 | 0.367 | 0.371 | 0.405 | 0.443 | 0.448 | 0.365 |
| R LimbicB OFC 2 | 0.66 | 0.022 | 0.613 | 0.621 | 0.661 | 0.694 | 0.7 | 0.755 | 0.401 | 0.02 | 0.365 | 0.369 | 0.4 | 0.437 | 0.444 | 0.147 |
| R LimbicB OFC 3 | 0.679 | 0.021 | 0.634 | 0.641 | 0.68 | 0.711 | 0.715 | 0.687 | 0.472 | 0.024 | 0.424 | 0.431 | 0.472 | 0.511 | 0.518 | 0.25 |
| R LimbicB OFC 4 | 0.697 | 0.02 | 0.656 | 0.662 | 0.698 | 0.728 | 0.732 | 0.5 | 0.434 | 0.026 | 0.385 | 0.39 | 0.435 | 0.477 | 0.485 | 0.329 |
| R LimbicA TempPole 1 | 0.653 | 0.023 | 0.606 | 0.613 | 0.655 | 0.687 | 0.694 | 0.686 | 0.477 | 0.024 | 0.427 | 0.436 | 0.478 | 0.516 | 0.524 | 0.117 |
| R LimbicA TempPole 2 | 0.717 | 0.019 | 0.677 | 0.684 | 0.718 | 0.747 | 0.751 | 0.564 | 0.435 | 0.025 | 0.387 | 0.395 | 0.434 | 0.476 | 0.483 | 0.42 |
| R LimbicA TempPole 3 | 0.671 | 0.022 | 0.623 | 0.634 | 0.672 | 0.703 | 0.71 | 0.681 | 0.568 | 0.024 | 0.519 | 0.527 | 0.569 | 0.607 | 0.613 | 0.133 |
| R LimbicA TempPole 4 | 0.691 | 0.021 | 0.647 | 0.656 | 0.692 | 0.723 | 0.728 | 0.794 | 0.459 | 0.025 | 0.412 | 0.42 | 0.459 | 0.501 | 0.509 | 0.431 |
| R ContA IPS 1 | 0.713 | 0.019 | 0.672 | 0.68 | 0.715 | 0.742 | 0.747 | 0.686 | 0.487 | 0.024 | 0.44 | 0.447 | 0.486 | 0.527 | 0.534 | 0.465 |
| R ContA IPS 2 | 0.729 | 0.018 | 0.69 | 0.698 | 0.73 | 0.757 | 0.76 | 0.662 | 0.441 | 0.023 | 0.396 | 0.402 | 0.44 | 0.481 | 0.487 | 0.443 |
| R ContA PFCd 1 | 0.662 | 0.022 | 0.615 | 0.622 | 0.663 | 0.697 | 0.701 | 0.731 | 0.462 | 0.024 | 0.416 | 0.423 | 0.462 | 0.502 | 0.51 | 0.412 |
| R ContA PFCl 1 | 0.672 | 0.022 | 0.628 | 0.635 | 0.673 | 0.705 | 0.711 | 0.684 | 0.431 | 0.023 | 0.387 | 0.393 | 0.43 | 0.469 | 0.477 | 0.48 |
| R ContA PFCl 2 | 0.648 | 0.023 | 0.6 | 0.607 | 0.649 | 0.684 | 0.688 | 0.671 | 0.422 | 0.023 | 0.379 | 0.385 | 0.421 | 0.461 | 0.468 | 0.289 |
| R ContA Cingm 1 | 0.715 | 0.019 | 0.676 | 0.681 | 0.716 | 0.743 | 0.748 | 0.342 | 0.394 | 0.018 | 0.36 | 0.365 | 0.393 | 0.424 | 0.431 | 0 |
| R ContB Temp 1 | 0.718 | 0.019 | 0.679 | 0.686 | 0.72 | 0.747 | 0.751 | 0.617 | 0.459 | 0.025 | 0.411 | 0.419 | 0.459 | 0.501 | 0.51 | 0.384 |
| R ContB Temp 2 | 0.685 | 0.021 | 0.639 | 0.647 | 0.686 | 0.717 | 0.721 | 0.554 | 0.428 | 0.023 | 0.385 | 0.391 | 0.428 | 0.466 | 0.474 | 0.285 |
| R ContB IPL 1 | 0.73 | 0.018 | 0.691 | 0.698 | 0.731 | 0.758 | 0.762 | 0.761 | 0.443 | 0.023 | 0.398 | 0.405 | 0.444 | 0.481 | 0.487 | 0.328 |
| R ContB IPL 2 | 0.726 | 0.018 | 0.687 | 0.694 | 0.728 | 0.755 | 0.759 | 0.653 | 0.509 | 0.024 | 0.463 | 0.469 | 0.509 | 0.549 | 0.557 | 0.564 |
| R ContB PFCld 1 | 0.708 | 0.02 | 0.667 | 0.674 | 0.708 | 0.738 | 0.742 | 0.683 | 0.429 | 0.023 | 0.385 | 0.393 | 0.428 | 0.468 | 0.474 | 0.289 |
| R ContB PFCld 2 | 0.66 | 0.022 | 0.614 | 0.62 | 0.662 | 0.695 | 0.7 | 0.691 | 0.438 | 0.024 | 0.393 | 0.399 | 0.438 | 0.477 | 0.485 | 0.354 |
| R ContB PFClv 1 | 0.651 | 0.023 | 0.602 | 0.611 | 0.652 | 0.686 | 0.692 | 0.55 | 0.394 | 0.018 | 0.361 | 0.365 | 0.393 | 0.425 | 0.431 | 0.071 |
| R ContB PFClv 2 | 0.713 | 0.019 | 0.671 | 0.68 | 0.714 | 0.742 | 0.748 | 0.625 | 0.468 | 0.025 | 0.419 | 0.426 | 0.468 | 0.51 | 0.517 | 0.368 |
| R ContB PFCmp 1 | 0.696 | 0.02 | 0.651 | 0.661 | 0.697 | 0.726 | 0.731 | 0.76 | 0.391 | 0.017 | 0.359 | 0.364 | 0.391 | 0.419 | 0.425 | 0.253 |
| R ContB PFCld 3 | 0.677 | 0.022 | 0.631 | 0.639 | 0.679 | 0.71 | 0.716 | 0.686 | 0.398 | 0.019 | 0.363 | 0.367 | 0.396 | 0.431 | 0.438 | 0.088 |
| R ContC pCun 1 | 0.669 | 0.022 | 0.625 | 0.631 | 0.671 | 0.704 | 0.708 | 0.62 | 0.427 | 0.023 | 0.383 | 0.389 | 0.426 | 0.467 | 0.473 | 0.325 |
| R ContC pCun 2 | 0.667 | 0.022 | 0.62 | 0.628 | 0.668 | 0.7 | 0.706 | 0.709 | 0.398 | 0.019 | 0.364 | 0.369 | 0.398 | 0.431 | 0.437 | 0.115 |
| R ContC Cingp 1 | 0.661 | 0.022 | 0.613 | 0.622 | 0.663 | 0.694 | 0.701 | 0.671 | 0.39 | 0.016 | 0.359 | 0.363 | 0.39 | 0.417 | 0.422 | 0 |
| R DefaultA IPL 1 | 0.702 | 0.02 | 0.662 | 0.668 | 0.704 | 0.733 | 0.738 | 0.673 | 0.397 | 0.019 | 0.362 | 0.367 | 0.397 | 0.431 | 0.436 | 0.356 |
| R DefaultA PFCd 1 | 0.683 | 0.021 | 0.639 | 0.646 | 0.684 | 0.715 | 0.719 | 0.665 | 0.39 | 0.016 | 0.359 | 0.363 | 0.39 | 0.418 | 0.423 | 0.189 |
| R DefaultA pCunPCC 1 | 0.649 | 0.023 | 0.6 | 0.607 | 0.65 | 0.685 | 0.69 | 0.7 | 0.391 | 0.017 | 0.36 | 0.364 | 0.391 | 0.42 | 0.426 | 0 |
| R DefaultA PFCm 1 | 0.646 | 0.023 | 0.597 | 0.605 | 0.648 | 0.682 | 0.688 | 0.589 | 0.413 | 0.021 | 0.372 | 0.378 | 0.412 | 0.448 | 0.456 | 0.183 |
| R DefaultA PFCm 2 | 0.697 | 0.02 | 0.654 | 0.661 | 0.698 | 0.727 | 0.732 | 0.53 | 0.462 | 0.024 | 0.414 | 0.422 | 0.461 | 0.501 | 0.51 | 0.232 |
| R DefaultA PFCm 3 | 0.673 | 0.022 | 0.627 | 0.635 | 0.674 | 0.705 | 0.711 | 0.57 | 0.408 | 0.021 | 0.37 | 0.374 | 0.409 | 0.444 | 0.451 | 0.302 |
| R DefaultB Temp 1 | 0.686 | 0.021 | 0.642 | 0.649 | 0.687 | 0.718 | 0.723 | 0.658 | 0.418 | 0.023 | 0.376 | 0.381 | 0.417 | 0.457 | 0.465 | 0.33 |
| R DefaultB AntTemp 1 | 0.687 | 0.02 | 0.642 | 0.65 | 0.688 | 0.718 | 0.723 | 0.624 | 0.393 | 0.018 | 0.361 | 0.365 | 0.393 | 0.423 | 0.43 | 0.052 |
| R DefaultB PFCd 1 | 0.715 | 0.019 | 0.674 | 0.682 | 0.716 | 0.745 | 0.749 | 0.701 | 0.394 | 0.018 | 0.362 | 0.365 | 0.394 | 0.424 | 0.431 | 0.161 |
| R DefaultB PFCv 1 | 0.663 | 0.022 | 0.616 | 0.625 | 0.664 | 0.696 | 0.702 | 0.727 | 0.406 | 0.022 | 0.365 | 0.372 | 0.405 | 0.444 | 0.452 | 0.367 |
| R DefaultC IPL 1 | 0.698 | 0.02 | 0.655 | 0.663 | 0.699 | 0.728 | 0.733 | 0.638 | 0.464 | 0.023 | 0.418 | 0.426 | 0.465 | 0.502 | 0.51 | 0.44 |
| R DefaultC Rsp 1 | 0.645 | 0.023 | 0.595 | 0.603 | 0.647 | 0.681 | 0.686 | 0.715 | 0.411 | 0.022 | 0.37 | 0.375 | 0.409 | 0.449 | 0.456 | 0.075 |
| R DefaultC PHC 1 | 0.639 | 0.024 | 0.59 | 0.598 | 0.64 | 0.675 | 0.681 | 0.708 | 0.4 | 0.02 | 0.364 | 0.369 | 0.399 | 0.435 | 0.441 | 0.303 |
| R TempPar 1 | 0.666 | 0.022 | 0.621 | 0.628 | 0.668 | 0.701 | 0.708 | 0.605 | 0.391 | 0.017 | 0.36 | 0.364 | 0.391 | 0.42 | 0.425 | 0.305 |
| R TempPar 2 | 0.672 | 0.022 | 0.626 | 0.634 | 0.673 | 0.704 | 0.71 | 0.674 | 0.395 | 0.019 | 0.362 | 0.367 | 0.395 | 0.426 | 0.434 | 0.211 |
| R TempPar 3 | 0.73 | 0.018 | 0.692 | 0.699 | 0.731 | 0.757 | 0.761 | 0.676 | 0.48 | 0.024 | 0.432 | 0.439 | 0.479 | 0.519 | 0.526 | 0.57 |
| R TempPar 4 | 0.692 | 0.02 | 0.65 | 0.657 | 0.693 | 0.724 | 0.728 | 0.541 | 0.401 | 0.02 | 0.365 | 0.369 | 0.4 | 0.436 | 0.442 | 0.378 |
| L Thalamus | 0.626 | 0.024 | 0.575 | 0.582 | 0.627 | 0.663 | 0.669 | 0.729 | 0.389 | 0.016 | 0.358 | 0.363 | 0.389 | 0.416 | 0.422 | 0.099 |
| L Caudate | 0.661 | 0.022 | 0.613 | 0.621 | 0.662 | 0.695 | 0.7 | 0.696 | 0.432 | 0.023 | 0.386 | 0.394 | 0.433 | 0.471 | 0.477 | 0.188 |
| L Putamen | 0.634 | 0.024 | 0.583 | 0.591 | 0.636 | 0.671 | 0.678 | 0.658 | 0.393 | 0.018 | 0.36 | 0.365 | 0.393 | 0.423 | 0.429 | 0.206 |
| L Pallidum | 0.651 | 0.023 | 0.601 | 0.61 | 0.652 | 0.687 | 0.691 | 0.637 | 0.393 | 0.017 | 0.36 | 0.366 | 0.392 | 0.422 | 0.428 | 0.068 |
| L Hippocampus | 0.629 | 0.024 | 0.579 | 0.587 | 0.631 | 0.667 | 0.673 | 0.715 | 0.393 | 0.018 | 0.361 | 0.365 | 0.393 | 0.425 | 0.43 | 0.037 |
| L Amygdala | 0.652 | 0.023 | 0.604 | 0.611 | 0.654 | 0.687 | 0.693 | 0.758 | 0.409 | 0.022 | 0.37 | 0.375 | 0.408 | 0.448 | 0.455 | 0.201 |
| L Accumbens | 0.679 | 0.021 | 0.634 | 0.642 | 0.681 | 0.711 | 0.717 | 0.708 | 0.498 | 0.025 | 0.45 | 0.457 | 0.498 | 0.539 | 0.545 | 0.018 |
| R Thalamus | 0.627 | 0.024 | 0.576 | 0.585 | 0.629 | 0.664 | 0.671 | 0.685 | 0.391 | 0.017 | 0.36 | 0.363 | 0.39 | 0.419 | 0.424 | 0.175 |
| R Caudate | 0.655 | 0.023 | 0.607 | 0.615 | 0.656 | 0.69 | 0.695 | 0.68 | 0.406 | 0.02 | 0.368 | 0.373 | 0.405 | 0.441 | 0.449 | 0.091 |
| R Putamen | 0.637 | 0.024 | 0.588 | 0.595 | 0.639 | 0.672 | 0.679 | 0.639 | 0.393 | 0.018 | 0.361 | 0.365 | 0.393 | 0.423 | 0.429 | 0.089 |
| R Pallidum | 0.665 | 0.022 | 0.619 | 0.627 | 0.666 | 0.7 | 0.705 | 0.622 | 0.396 | 0.019 | 0.361 | 0.366 | 0.395 | 0.426 | 0.433 | 0 |
| R Hippocampus | 0.633 | 0.024 | 0.583 | 0.591 | 0.635 | 0.67 | 0.676 | 0.654 | 0.39 | 0.016 | 0.359 | 0.363 | 0.389 | 0.417 | 0.422 | 0 |
| R Amygdala | 0.659 | 0.023 | 0.611 | 0.621 | 0.66 | 0.693 | 0.699 | 0.661 | 0.404 | 0.022 | 0.366 | 0.372 | 0.403 | 0.442 | 0.452 | 0.246 |
| R Accumbens | 0.687 | 0.021 | 0.643 | 0.652 | 0.689 | 0.719 | 0.724 | 0.679 | 0.471 | 0.025 | 0.424 | 0.431 | 0.471 | 0.51 | 0.518 | 0.028 |

*Table S14.* ROI-level reliability for the visual search face vs. scramble and log-transformed slope contrasts: Bayesian multilevel model (BMM) and conventional linear mixed-effects model (ICC) for 200 cortical parcels and 14 subcortical ROIs

|  | **Face vs. Scramble Control** | | | | | | | | **Log-transformed Slope** | | | | | | | |
| --- | --- | --- | --- | --- | --- | --- | --- | --- | --- | --- | --- | --- | --- | --- | --- | --- |
| **Label** | **BMM** | **SD** | **2.50%** | **5%** | **50%** | **95%** | **97.50%** | **ICC** | **BMM** | **SD** | **2.50%** | **5%** | **50%** | **95%** | **97.50%** | **ICC** |
| L VisCent ExStr 1 | 0.189 | 0.044 | 0.101 | 0.115 | 0.189 | 0.259 | 0.272 | 0.094 | 0.561 | 0.026 | 0.508 | 0.518 | 0.562 | 0.601 | 0.609 | 0.267 |
| L VisCent ExStr 2 | 0.268 | 0.043 | 0.183 | 0.196 | 0.268 | 0.338 | 0.351 | 0.026 | 0.568 | 0.026 | 0.514 | 0.524 | 0.57 | 0.61 | 0.618 | 0.386 |
| L VisCent Striate 1 | 0.135 | 0.044 | 0.055 | 0.066 | 0.133 | 0.211 | 0.224 | 0 | 0.572 | 0.027 | 0.52 | 0.528 | 0.573 | 0.614 | 0.621 | 0.217 |
| L VisCent ExStr 3 | 0.193 | 0.046 | 0.104 | 0.118 | 0.194 | 0.272 | 0.285 | 0.128 | 0.6 | 0.022 | 0.557 | 0.564 | 0.601 | 0.635 | 0.642 | 0.455 |
| L VisCent ExStr 4 | 0.064 | 0.031 | 0.027 | 0.028 | 0.056 | 0.125 | 0.138 | 0.131 | 0.462 | 0.03 | 0.404 | 0.413 | 0.462 | 0.511 | 0.518 | 0.264 |
| L VisCent ExStr 5 | 0.042 | 0.017 | 0.025 | 0.026 | 0.038 | 0.076 | 0.085 | 0 | 0.505 | 0.026 | 0.453 | 0.462 | 0.506 | 0.548 | 0.558 | 0.333 |
| L VisPeri ExStrInf 1 | 0.038 | 0.012 | 0.024 | 0.025 | 0.034 | 0.061 | 0.07 | 0 | 0.316 | 0.029 | 0.264 | 0.272 | 0.314 | 0.365 | 0.375 | 0.289 |
| L VisPeri ExStrInf 2 | 0.191 | 0.045 | 0.103 | 0.117 | 0.191 | 0.265 | 0.277 | 0.087 | 0.418 | 0.032 | 0.355 | 0.367 | 0.418 | 0.472 | 0.482 | 0.161 |
| L VisPeri ExStrInf 3 | 0.087 | 0.041 | 0.029 | 0.032 | 0.081 | 0.162 | 0.178 | 0 | 0.331 | 0.032 | 0.27 | 0.28 | 0.329 | 0.384 | 0.399 | 0 |
| L VisPeri StriCal 1 | 0.091 | 0.039 | 0.03 | 0.035 | 0.088 | 0.163 | 0.177 | 0 | 0.432 | 0.03 | 0.372 | 0.384 | 0.432 | 0.481 | 0.489 | 0.297 |
| L VisPeri ExStrSup 1 | 0.051 | 0.023 | 0.025 | 0.026 | 0.044 | 0.1 | 0.113 | 0.08 | 0.244 | 0.017 | 0.218 | 0.221 | 0.242 | 0.274 | 0.285 | 0.123 |
| L VisPeri ExStrSup 2 | 0.069 | 0.033 | 0.026 | 0.028 | 0.063 | 0.134 | 0.148 | 0 | 0.354 | 0.032 | 0.295 | 0.304 | 0.353 | 0.408 | 0.42 | 0.141 |
| L SomMotA 1 | 0.039 | 0.013 | 0.024 | 0.025 | 0.035 | 0.065 | 0.075 | 0 | 0.237 | 0.013 | 0.216 | 0.219 | 0.235 | 0.261 | 0.268 | 0.035 |
| L SomMotA 2 | 0.07 | 0.034 | 0.027 | 0.029 | 0.063 | 0.136 | 0.148 | 0 | 0.237 | 0.013 | 0.216 | 0.218 | 0.235 | 0.262 | 0.269 | 0 |
| L SomMotA 3 | 0.067 | 0.032 | 0.027 | 0.028 | 0.061 | 0.127 | 0.139 | 0.009 | 0.238 | 0.013 | 0.217 | 0.22 | 0.236 | 0.263 | 0.27 | 0 |
| L SomMotA 4 | 0.044 | 0.017 | 0.024 | 0.025 | 0.039 | 0.081 | 0.091 | 0.018 | 0.236 | 0.012 | 0.216 | 0.218 | 0.234 | 0.259 | 0.264 | 0 |
| L SomMotA 5 | 0.038 | 0.013 | 0.024 | 0.025 | 0.035 | 0.063 | 0.072 | 0 | 0.236 | 0.012 | 0.216 | 0.219 | 0.234 | 0.259 | 0.265 | 0 |
| L SomMotA 6 | 0.044 | 0.017 | 0.025 | 0.026 | 0.039 | 0.081 | 0.091 | 0 | 0.236 | 0.013 | 0.216 | 0.219 | 0.235 | 0.261 | 0.267 | 0 |
| L SomMotA 7 | 0.044 | 0.017 | 0.025 | 0.026 | 0.038 | 0.079 | 0.091 | 0 | 0.238 | 0.014 | 0.216 | 0.22 | 0.236 | 0.264 | 0.271 | 0.075 |
| L SomMotA 8 | 0.051 | 0.024 | 0.025 | 0.026 | 0.043 | 0.097 | 0.111 | 0 | 0.238 | 0.014 | 0.215 | 0.219 | 0.235 | 0.263 | 0.27 | 0 |
| L SomMotB Aud 1 | 0.043 | 0.017 | 0.024 | 0.026 | 0.038 | 0.08 | 0.09 | 0 | 0.24 | 0.015 | 0.217 | 0.22 | 0.238 | 0.268 | 0.274 | 0 |
| L SomMotB Aud 2 | 0.039 | 0.012 | 0.024 | 0.025 | 0.035 | 0.062 | 0.07 | 0 | 0.239 | 0.014 | 0.217 | 0.22 | 0.237 | 0.267 | 0.273 | 0.11 |
| L SomMotB S2 1 | 0.047 | 0.02 | 0.025 | 0.026 | 0.042 | 0.085 | 0.1 | 0 | 0.236 | 0.013 | 0.216 | 0.219 | 0.234 | 0.26 | 0.266 | 0 |
| L SomMotB S2 2 | 0.038 | 0.012 | 0.024 | 0.025 | 0.035 | 0.061 | 0.071 | 0 | 0.236 | 0.013 | 0.216 | 0.219 | 0.234 | 0.26 | 0.265 | 0 |
| L SomMotB Aud 3 | 0.038 | 0.012 | 0.024 | 0.025 | 0.035 | 0.061 | 0.068 | 0 | 0.237 | 0.013 | 0.215 | 0.219 | 0.235 | 0.262 | 0.268 | 0 |
| L SomMotB S2 3 | 0.046 | 0.021 | 0.025 | 0.026 | 0.04 | 0.091 | 0.102 | 0 | 0.241 | 0.016 | 0.217 | 0.22 | 0.239 | 0.271 | 0.279 | 0.062 |
| L SomMotB Cent 1 | 0.041 | 0.015 | 0.024 | 0.025 | 0.037 | 0.073 | 0.082 | 0 | 0.241 | 0.015 | 0.217 | 0.221 | 0.238 | 0.269 | 0.277 | 0.085 |
| L SomMotB Cent 2 | 0.038 | 0.012 | 0.024 | 0.025 | 0.035 | 0.063 | 0.07 | 0.039 | 0.236 | 0.012 | 0.215 | 0.218 | 0.234 | 0.259 | 0.266 | 0 |
| L DorsAttnA TempOcc 1 | 0.219 | 0.05 | 0.119 | 0.136 | 0.219 | 0.297 | 0.311 | 0.235 | 0.271 | 0.026 | 0.228 | 0.234 | 0.269 | 0.316 | 0.325 | 0.092 |
| L DorsAttnA TempOcc 2 | 0.127 | 0.043 | 0.042 | 0.054 | 0.126 | 0.199 | 0.217 | 0.12 | 0.378 | 0.03 | 0.322 | 0.331 | 0.378 | 0.427 | 0.435 | 0.228 |
| L DorsAttnA ParOcc 1 | 0.04 | 0.015 | 0.024 | 0.025 | 0.036 | 0.07 | 0.078 | 0 | 0.325 | 0.029 | 0.27 | 0.28 | 0.324 | 0.374 | 0.384 | 0.31 |
| L DorsAttnA SPL 1 | 0.038 | 0.012 | 0.024 | 0.025 | 0.035 | 0.061 | 0.071 | 0.134 | 0.442 | 0.029 | 0.386 | 0.396 | 0.443 | 0.49 | 0.497 | 0.492 |
| L DorsAttnA SPL 2 | 0.103 | 0.043 | 0.032 | 0.038 | 0.101 | 0.176 | 0.192 | 0.006 | 0.567 | 0.026 | 0.512 | 0.521 | 0.568 | 0.609 | 0.617 | 0.509 |
| L DorsAttnA SPL 3 | 0.044 | 0.018 | 0.025 | 0.026 | 0.039 | 0.081 | 0.092 | 0 | 0.511 | 0.03 | 0.449 | 0.46 | 0.512 | 0.56 | 0.567 | 0.438 |
| L DorsAttnB PostC 1 | 0.037 | 0.011 | 0.024 | 0.025 | 0.034 | 0.06 | 0.067 | 0 | 0.236 | 0.013 | 0.215 | 0.218 | 0.234 | 0.259 | 0.265 | 0 |
| L DorsAttnB PostC 2 | 0.04 | 0.015 | 0.024 | 0.025 | 0.037 | 0.07 | 0.08 | 0.048 | 0.243 | 0.017 | 0.218 | 0.221 | 0.24 | 0.274 | 0.284 | 0 |
| L DorsAttnB PostC 3 | 0.044 | 0.017 | 0.025 | 0.026 | 0.039 | 0.08 | 0.087 | 0 | 0.243 | 0.017 | 0.217 | 0.22 | 0.241 | 0.276 | 0.283 | 0 |
| L DorsAttnB PostC 4 | 0.04 | 0.014 | 0.024 | 0.026 | 0.036 | 0.068 | 0.078 | 0 | 0.238 | 0.014 | 0.216 | 0.219 | 0.235 | 0.263 | 0.272 | 0 |
| L DorsAttnB FEF 1 | 0.04 | 0.014 | 0.024 | 0.025 | 0.036 | 0.068 | 0.076 | 0.08 | 0.24 | 0.015 | 0.215 | 0.219 | 0.238 | 0.269 | 0.276 | 0.2 |
| L SalVentAttnA ParOper 1 | 0.038 | 0.012 | 0.024 | 0.025 | 0.035 | 0.061 | 0.07 | 0 | 0.235 | 0.012 | 0.215 | 0.218 | 0.234 | 0.258 | 0.264 | 0 |
| L SalVentAttnA Ins 1 | 0.036 | 0.01 | 0.024 | 0.025 | 0.034 | 0.057 | 0.063 | 0 | 0.236 | 0.012 | 0.216 | 0.218 | 0.234 | 0.258 | 0.263 | 0 |
| L SalVentAttnA FrOper 1 | 0.044 | 0.017 | 0.024 | 0.025 | 0.039 | 0.078 | 0.088 | 0 | 0.236 | 0.012 | 0.215 | 0.219 | 0.234 | 0.258 | 0.264 | 0 |
| L SalVentAttnA FrOper 2 | 0.041 | 0.015 | 0.024 | 0.025 | 0.037 | 0.072 | 0.082 | 0 | 0.236 | 0.013 | 0.216 | 0.219 | 0.234 | 0.26 | 0.264 | 0.086 |
| L SalVentAttnA ParMed 1 | 0.04 | 0.014 | 0.024 | 0.025 | 0.036 | 0.068 | 0.078 | 0.019 | 0.237 | 0.013 | 0.216 | 0.219 | 0.235 | 0.26 | 0.266 | 0.12 |
| L SalVentAttnA FrMed 1 | 0.039 | 0.013 | 0.024 | 0.025 | 0.035 | 0.064 | 0.073 | 0 | 0.25 | 0.02 | 0.22 | 0.223 | 0.248 | 0.288 | 0.297 | 0 |
| L SalVentAttnA FrMed 2 | 0.065 | 0.031 | 0.026 | 0.028 | 0.059 | 0.125 | 0.134 | 0 | 0.237 | 0.013 | 0.216 | 0.219 | 0.235 | 0.261 | 0.267 | 0 |
| L SalVentAttnB IPL 1 | 0.048 | 0.021 | 0.025 | 0.026 | 0.041 | 0.089 | 0.105 | 0.112 | 0.239 | 0.014 | 0.217 | 0.22 | 0.237 | 0.265 | 0.272 | 0.075 |
| L SalVentAttnB PFCl 1 | 0.051 | 0.024 | 0.025 | 0.027 | 0.044 | 0.098 | 0.111 | 0.114 | 0.236 | 0.012 | 0.216 | 0.218 | 0.234 | 0.259 | 0.265 | 0.116 |
| L SalVentAttnB Ins 1 | 0.04 | 0.014 | 0.024 | 0.025 | 0.037 | 0.069 | 0.078 | 0 | 0.236 | 0.013 | 0.215 | 0.218 | 0.234 | 0.26 | 0.265 | 0 |
| L SalVentAttnB PFCmp 1 | 0.083 | 0.038 | 0.028 | 0.031 | 0.078 | 0.153 | 0.17 | 0 | 0.241 | 0.016 | 0.217 | 0.22 | 0.239 | 0.271 | 0.279 | 0.021 |
| L LimbicB OFC 1 | 0.079 | 0.037 | 0.028 | 0.03 | 0.074 | 0.149 | 0.163 | 0.072 | 0.238 | 0.014 | 0.216 | 0.219 | 0.236 | 0.263 | 0.27 | 0 |
| L LimbicB OFC 2 | 0.165 | 0.045 | 0.081 | 0.096 | 0.162 | 0.242 | 0.254 | 0.134 | 0.268 | 0.026 | 0.225 | 0.23 | 0.265 | 0.316 | 0.328 | 0.095 |
| L LimbicA TempPole 1 | 0.107 | 0.043 | 0.03 | 0.037 | 0.106 | 0.181 | 0.195 | 0 | 0.388 | 0.032 | 0.325 | 0.334 | 0.388 | 0.438 | 0.448 | 0.13 |
| L LimbicA TempPole 2 | 0.137 | 0.045 | 0.052 | 0.066 | 0.135 | 0.214 | 0.228 | 0.018 | 0.32 | 0.031 | 0.259 | 0.268 | 0.319 | 0.37 | 0.379 | 0.039 |
| L LimbicA TempPole 3 | 0.302 | 0.042 | 0.219 | 0.233 | 0.302 | 0.368 | 0.381 | 0 | 0.411 | 0.032 | 0.349 | 0.358 | 0.411 | 0.463 | 0.47 | 0 |
| L LimbicA TempPole 4 | 0.221 | 0.044 | 0.133 | 0.149 | 0.221 | 0.293 | 0.307 | 0.001 | 0.41 | 0.032 | 0.348 | 0.358 | 0.409 | 0.463 | 0.469 | 0.145 |
| L ContA Temp 1 | 0.046 | 0.02 | 0.024 | 0.026 | 0.04 | 0.085 | 0.098 | 0 | 0.319 | 0.032 | 0.261 | 0.269 | 0.319 | 0.37 | 0.381 | 0.17 |
| L ContA IPS 1 | 0.053 | 0.025 | 0.025 | 0.027 | 0.046 | 0.103 | 0.115 | 0.052 | 0.336 | 0.031 | 0.279 | 0.286 | 0.336 | 0.387 | 0.399 | 0.359 |
| L ContA IPS 2 | 0.047 | 0.021 | 0.025 | 0.026 | 0.04 | 0.09 | 0.101 | 0.031 | 0.263 | 0.024 | 0.225 | 0.228 | 0.261 | 0.304 | 0.313 | 0.152 |
| L ContA IPS 3 | 0.045 | 0.019 | 0.024 | 0.026 | 0.039 | 0.082 | 0.094 | 0.063 | 0.261 | 0.026 | 0.221 | 0.226 | 0.257 | 0.308 | 0.322 | 0.258 |
| L ContA PFCd 1 | 0.048 | 0.022 | 0.025 | 0.026 | 0.042 | 0.096 | 0.109 | 0.007 | 0.237 | 0.013 | 0.216 | 0.219 | 0.235 | 0.261 | 0.267 | 0.028 |
| L ContA PFClv 1 | 0.038 | 0.012 | 0.024 | 0.025 | 0.035 | 0.062 | 0.068 | 0.057 | 0.238 | 0.013 | 0.216 | 0.219 | 0.236 | 0.263 | 0.269 | 0.241 |
| L ContA PFCl 1 | 0.053 | 0.024 | 0.026 | 0.027 | 0.046 | 0.102 | 0.116 | 0.117 | 0.24 | 0.015 | 0.217 | 0.22 | 0.237 | 0.269 | 0.276 | 0.094 |
| L ContA PFCl 2 | 0.043 | 0.017 | 0.025 | 0.025 | 0.038 | 0.079 | 0.087 | 0.018 | 0.289 | 0.028 | 0.233 | 0.243 | 0.288 | 0.337 | 0.347 | 0.293 |
| L ContA PFCl 3 | 0.04 | 0.014 | 0.024 | 0.025 | 0.036 | 0.066 | 0.076 | 0.083 | 0.257 | 0.023 | 0.222 | 0.225 | 0.254 | 0.3 | 0.309 | 0.293 |
| L ContA Cingm 1 | 0.122 | 0.044 | 0.04 | 0.052 | 0.122 | 0.196 | 0.21 | 0 | 0.398 | 0.033 | 0.338 | 0.346 | 0.398 | 0.454 | 0.465 | 0.241 |
| L ContB Temp 1 | 0.096 | 0.038 | 0.031 | 0.037 | 0.094 | 0.163 | 0.175 | 0.123 | 0.247 | 0.019 | 0.22 | 0.222 | 0.244 | 0.281 | 0.291 | 0.155 |
| L ContB IPL 1 | 0.044 | 0.018 | 0.025 | 0.026 | 0.038 | 0.08 | 0.094 | 0 | 0.24 | 0.015 | 0.217 | 0.219 | 0.238 | 0.266 | 0.273 | 0.106 |
| L ContB PFCl 1 | 0.049 | 0.022 | 0.025 | 0.026 | 0.043 | 0.094 | 0.107 | 0.112 | 0.238 | 0.014 | 0.216 | 0.219 | 0.236 | 0.263 | 0.27 | 0 |
| L ContB PFClv 1 | 0.106 | 0.04 | 0.036 | 0.041 | 0.103 | 0.175 | 0.187 | 0.01 | 0.264 | 0.026 | 0.224 | 0.228 | 0.262 | 0.31 | 0.319 | 0.04 |
| L ContB PFClv 2 | 0.131 | 0.044 | 0.05 | 0.061 | 0.129 | 0.203 | 0.221 | 0.141 | 0.246 | 0.017 | 0.219 | 0.222 | 0.243 | 0.278 | 0.287 | 0 |
| L ContC pCun 1 | 0.178 | 0.045 | 0.09 | 0.102 | 0.178 | 0.253 | 0.267 | 0 | 0.321 | 0.029 | 0.267 | 0.274 | 0.32 | 0.37 | 0.381 | 0.165 |
| L ContC pCun 2 | 0.185 | 0.046 | 0.096 | 0.109 | 0.184 | 0.263 | 0.277 | 0.087 | 0.282 | 0.029 | 0.233 | 0.239 | 0.281 | 0.334 | 0.345 | 0.07 |
| L ContC Cingp 1 | 0.193 | 0.043 | 0.111 | 0.122 | 0.193 | 0.264 | 0.277 | 0.067 | 0.303 | 0.031 | 0.242 | 0.251 | 0.302 | 0.356 | 0.368 | 0.187 |
| L DefaultA IPL 1 | 0.061 | 0.03 | 0.026 | 0.027 | 0.053 | 0.122 | 0.138 | 0 | 0.271 | 0.029 | 0.223 | 0.228 | 0.268 | 0.322 | 0.335 | 0.161 |
| L DefaultA PFCd 1 | 0.041 | 0.014 | 0.024 | 0.026 | 0.037 | 0.068 | 0.078 | 0.021 | 0.238 | 0.013 | 0.216 | 0.219 | 0.236 | 0.262 | 0.269 | 0.007 |
| L DefaultA pCunPCC 1 | 0.314 | 0.043 | 0.226 | 0.243 | 0.316 | 0.383 | 0.398 | 0.068 | 0.351 | 0.031 | 0.292 | 0.3 | 0.351 | 0.402 | 0.412 | 0 |
| L DefaultA pCunPCC 2 | 0.179 | 0.045 | 0.09 | 0.104 | 0.179 | 0.253 | 0.266 | 0.036 | 0.281 | 0.03 | 0.23 | 0.236 | 0.28 | 0.333 | 0.343 | 0.11 |
| L DefaultA pCunPCC 3 | 0.137 | 0.046 | 0.054 | 0.066 | 0.135 | 0.216 | 0.232 | 0.114 | 0.261 | 0.026 | 0.22 | 0.225 | 0.258 | 0.307 | 0.317 | 0.03 |
| L DefaultA PFCm 1 | 0.368 | 0.039 | 0.289 | 0.304 | 0.369 | 0.428 | 0.442 | 0.342 | 0.29 | 0.03 | 0.234 | 0.243 | 0.289 | 0.343 | 0.349 | 0.036 |
| L DefaultA PFCm 2 | 0.08 | 0.036 | 0.028 | 0.031 | 0.077 | 0.146 | 0.162 | 0.151 | 0.237 | 0.013 | 0.216 | 0.219 | 0.235 | 0.262 | 0.268 | 0 |
| L DefaultA PFCm 3 | 0.179 | 0.043 | 0.097 | 0.109 | 0.179 | 0.25 | 0.264 | 0.053 | 0.24 | 0.015 | 0.217 | 0.22 | 0.237 | 0.268 | 0.274 | 0 |
| L DefaultB Temp 1 | 0.059 | 0.027 | 0.026 | 0.028 | 0.052 | 0.113 | 0.126 | 0 | 0.247 | 0.018 | 0.22 | 0.223 | 0.244 | 0.28 | 0.292 | 0 |
| L DefaultB Temp 2 | 0.074 | 0.036 | 0.027 | 0.029 | 0.068 | 0.144 | 0.16 | 0 | 0.243 | 0.017 | 0.218 | 0.221 | 0.24 | 0.276 | 0.283 | 0 |
| L DefaultB Temp 3 | 0.039 | 0.013 | 0.024 | 0.025 | 0.035 | 0.065 | 0.073 | 0 | 0.236 | 0.012 | 0.215 | 0.218 | 0.234 | 0.259 | 0.265 | 0 |
| L DefaultB Temp 4 | 0.042 | 0.017 | 0.025 | 0.025 | 0.037 | 0.074 | 0.086 | 0 | 0.236 | 0.013 | 0.215 | 0.219 | 0.234 | 0.26 | 0.266 | 0.03 |
| L DefaultB IPL 1 | 0.037 | 0.012 | 0.024 | 0.025 | 0.034 | 0.06 | 0.068 | 0 | 0.237 | 0.013 | 0.216 | 0.219 | 0.236 | 0.262 | 0.269 | 0 |
| L DefaultB PFCd 1 | 0.074 | 0.036 | 0.027 | 0.028 | 0.067 | 0.144 | 0.156 | 0 | 0.238 | 0.014 | 0.216 | 0.219 | 0.236 | 0.264 | 0.27 | 0.046 |
| L DefaultB PFCd 2 | 0.078 | 0.036 | 0.027 | 0.03 | 0.074 | 0.145 | 0.159 | 0.109 | 0.249 | 0.02 | 0.22 | 0.223 | 0.246 | 0.287 | 0.296 | 0 |
| L DefaultB PFCd 3 | 0.039 | 0.013 | 0.024 | 0.025 | 0.035 | 0.064 | 0.071 | 0 | 0.237 | 0.013 | 0.216 | 0.219 | 0.235 | 0.261 | 0.269 | 0.181 |
| L DefaultB PFCd 4 | 0.058 | 0.028 | 0.025 | 0.027 | 0.05 | 0.113 | 0.127 | 0.01 | 0.236 | 0.013 | 0.215 | 0.218 | 0.235 | 0.26 | 0.265 | 0 |
| L DefaultB PFCv 1 | 0.039 | 0.013 | 0.024 | 0.025 | 0.035 | 0.064 | 0.074 | 0 | 0.237 | 0.013 | 0.216 | 0.219 | 0.235 | 0.261 | 0.268 | 0.042 |
| L DefaultB PFCv 2 | 0.041 | 0.015 | 0.024 | 0.025 | 0.036 | 0.071 | 0.079 | 0 | 0.238 | 0.013 | 0.217 | 0.219 | 0.236 | 0.262 | 0.268 | 0 |
| L DefaultB PFCv 3 | 0.05 | 0.022 | 0.025 | 0.026 | 0.044 | 0.095 | 0.106 | 0.009 | 0.249 | 0.02 | 0.219 | 0.222 | 0.246 | 0.288 | 0.296 | 0.054 |
| L DefaultB PFCv 4 | 0.048 | 0.022 | 0.025 | 0.026 | 0.042 | 0.094 | 0.11 | 0.079 | 0.238 | 0.013 | 0.216 | 0.219 | 0.236 | 0.262 | 0.268 | 0.008 |
| L DefaultC IPL 1 | 0.076 | 0.037 | 0.028 | 0.03 | 0.069 | 0.146 | 0.163 | 0.134 | 0.324 | 0.029 | 0.269 | 0.278 | 0.323 | 0.373 | 0.382 | 0.098 |
| L DefaultC Rsp 1 | 0.302 | 0.047 | 0.209 | 0.224 | 0.302 | 0.38 | 0.396 | 0.1 | 0.304 | 0.032 | 0.243 | 0.253 | 0.303 | 0.36 | 0.37 | 0 |
| L DefaultC PHC 1 | 0.091 | 0.041 | 0.029 | 0.032 | 0.089 | 0.164 | 0.176 | 0 | 0.244 | 0.017 | 0.217 | 0.221 | 0.241 | 0.275 | 0.284 | 0 |
| L TempPar 1 | 0.038 | 0.012 | 0.024 | 0.025 | 0.035 | 0.06 | 0.069 | 0 | 0.236 | 0.013 | 0.215 | 0.218 | 0.234 | 0.26 | 0.264 | 0.027 |
| L TempPar 2 | 0.044 | 0.018 | 0.024 | 0.026 | 0.04 | 0.079 | 0.089 | 0.043 | 0.258 | 0.023 | 0.222 | 0.226 | 0.256 | 0.3 | 0.309 | 0.219 |
| R VisCent ExStr 1 | 0.096 | 0.039 | 0.031 | 0.037 | 0.092 | 0.165 | 0.183 | 0.053 | 0.472 | 0.028 | 0.415 | 0.427 | 0.472 | 0.517 | 0.527 | 0.328 |
| R VisCent ExStr 2 | 0.316 | 0.041 | 0.23 | 0.248 | 0.317 | 0.38 | 0.392 | 0.248 | 0.391 | 0.03 | 0.335 | 0.342 | 0.39 | 0.44 | 0.451 | 0.071 |
| R VisCent Striate 1 | 0.245 | 0.044 | 0.159 | 0.173 | 0.244 | 0.316 | 0.329 | 0.032 | 0.595 | 0.024 | 0.547 | 0.555 | 0.596 | 0.634 | 0.641 | 0.182 |
| R VisCent ExStr 3 | 0.375 | 0.04 | 0.293 | 0.306 | 0.376 | 0.442 | 0.452 | 0.179 | 0.528 | 0.028 | 0.473 | 0.481 | 0.529 | 0.573 | 0.58 | 0.273 |
| R VisCent ExStr 4 | 0.112 | 0.042 | 0.041 | 0.05 | 0.109 | 0.186 | 0.204 | 0.207 | 0.428 | 0.03 | 0.37 | 0.378 | 0.428 | 0.476 | 0.486 | 0.083 |
| R VisCent ExStr 5 | 0.164 | 0.045 | 0.082 | 0.092 | 0.163 | 0.239 | 0.255 | 0.167 | 0.546 | 0.025 | 0.495 | 0.503 | 0.547 | 0.586 | 0.594 | 0.333 |
| R VisPeri ExStrInf 1 | 0.045 | 0.019 | 0.025 | 0.026 | 0.039 | 0.084 | 0.094 | 0 | 0.321 | 0.031 | 0.264 | 0.271 | 0.321 | 0.372 | 0.381 | 0.157 |
| R VisPeri ExStrInf 2 | 0.069 | 0.033 | 0.026 | 0.028 | 0.063 | 0.132 | 0.145 | 0 | 0.3 | 0.03 | 0.243 | 0.251 | 0.298 | 0.35 | 0.358 | 0.051 |
| R VisPeri StriCal 1 | 0.129 | 0.047 | 0.038 | 0.053 | 0.127 | 0.209 | 0.223 | 0.108 | 0.444 | 0.031 | 0.383 | 0.392 | 0.445 | 0.493 | 0.503 | 0.197 |
| R VisPeri ExStrSup 1 | 0.052 | 0.025 | 0.025 | 0.026 | 0.044 | 0.099 | 0.116 | 0 | 0.246 | 0.018 | 0.219 | 0.223 | 0.243 | 0.281 | 0.288 | 0 |
| R VisPeri ExStrSup 2 | 0.04 | 0.015 | 0.024 | 0.026 | 0.036 | 0.069 | 0.081 | 0 | 0.247 | 0.019 | 0.219 | 0.222 | 0.244 | 0.283 | 0.294 | 0.142 |
| R VisPeri ExStrSup 3 | 0.142 | 0.044 | 0.06 | 0.072 | 0.14 | 0.219 | 0.233 | 0.043 | 0.409 | 0.03 | 0.349 | 0.36 | 0.409 | 0.458 | 0.467 | 0.14 |
| R SomMotA 1 | 0.064 | 0.031 | 0.026 | 0.028 | 0.058 | 0.123 | 0.136 | 0 | 0.24 | 0.015 | 0.217 | 0.22 | 0.237 | 0.269 | 0.277 | 0 |
| R SomMotA 2 | 0.076 | 0.035 | 0.027 | 0.03 | 0.071 | 0.143 | 0.156 | 0 | 0.241 | 0.016 | 0.217 | 0.22 | 0.238 | 0.271 | 0.278 | 0 |
| R SomMotA 3 | 0.039 | 0.014 | 0.024 | 0.025 | 0.035 | 0.066 | 0.074 | 0 | 0.236 | 0.013 | 0.215 | 0.218 | 0.234 | 0.261 | 0.267 | 0.011 |
| R SomMotA 4 | 0.099 | 0.041 | 0.031 | 0.035 | 0.097 | 0.171 | 0.186 | 0 | 0.244 | 0.018 | 0.217 | 0.221 | 0.242 | 0.276 | 0.285 | 0 |
| R SomMotA 5 | 0.066 | 0.033 | 0.026 | 0.028 | 0.057 | 0.129 | 0.142 | 0 | 0.25 | 0.021 | 0.22 | 0.223 | 0.246 | 0.292 | 0.301 | 0 |
| R SomMotA 6 | 0.055 | 0.025 | 0.026 | 0.027 | 0.048 | 0.104 | 0.118 | 0 | 0.238 | 0.013 | 0.216 | 0.219 | 0.236 | 0.263 | 0.269 | 0 |
| R SomMotA 7 | 0.043 | 0.017 | 0.025 | 0.026 | 0.038 | 0.078 | 0.089 | 0 | 0.236 | 0.013 | 0.215 | 0.218 | 0.234 | 0.259 | 0.266 | 0 |
| R SomMotA 8 | 0.046 | 0.02 | 0.025 | 0.026 | 0.041 | 0.085 | 0.096 | 0 | 0.235 | 0.012 | 0.215 | 0.218 | 0.234 | 0.257 | 0.263 | 0 |
| R SomMotA 9 | 0.04 | 0.013 | 0.024 | 0.026 | 0.037 | 0.066 | 0.073 | 0 | 0.238 | 0.014 | 0.216 | 0.219 | 0.236 | 0.263 | 0.269 | 0 |
| R SomMotA 10 | 0.039 | 0.013 | 0.024 | 0.025 | 0.035 | 0.064 | 0.071 | 0 | 0.237 | 0.013 | 0.216 | 0.219 | 0.234 | 0.261 | 0.267 | 0.076 |
| R SomMotA 11 | 0.042 | 0.016 | 0.024 | 0.025 | 0.037 | 0.075 | 0.084 | 0 | 0.237 | 0.014 | 0.216 | 0.219 | 0.235 | 0.263 | 0.269 | 0 |
| R SomMotB Aud 1 | 0.059 | 0.028 | 0.026 | 0.028 | 0.052 | 0.116 | 0.13 | 0 | 0.241 | 0.016 | 0.217 | 0.22 | 0.239 | 0.271 | 0.281 | 0.023 |
| R SomMotB Aud 2 | 0.044 | 0.018 | 0.024 | 0.026 | 0.038 | 0.078 | 0.092 | 0 | 0.239 | 0.014 | 0.217 | 0.22 | 0.237 | 0.264 | 0.273 | 0.139 |
| R SomMotB S2 1 | 0.04 | 0.015 | 0.024 | 0.025 | 0.036 | 0.069 | 0.08 | 0 | 0.239 | 0.015 | 0.217 | 0.22 | 0.237 | 0.267 | 0.275 | 0.14 |
| R SomMotB S2 2 | 0.044 | 0.018 | 0.025 | 0.026 | 0.04 | 0.079 | 0.089 | 0 | 0.246 | 0.019 | 0.218 | 0.222 | 0.242 | 0.282 | 0.292 | 0.037 |
| R SomMotB S2 3 | 0.056 | 0.028 | 0.025 | 0.026 | 0.049 | 0.111 | 0.127 | 0 | 0.263 | 0.026 | 0.222 | 0.226 | 0.26 | 0.311 | 0.321 | 0 |
| R SomMotB S2 4 | 0.07 | 0.035 | 0.026 | 0.028 | 0.063 | 0.138 | 0.155 | 0 | 0.243 | 0.017 | 0.218 | 0.22 | 0.241 | 0.276 | 0.285 | 0 |
| R SomMotB Cent 1 | 0.055 | 0.026 | 0.025 | 0.027 | 0.047 | 0.107 | 0.118 | 0 | 0.239 | 0.014 | 0.216 | 0.219 | 0.236 | 0.266 | 0.271 | 0 |
| R DorsAttnA TempOcc 1 | 0.214 | 0.045 | 0.127 | 0.14 | 0.214 | 0.286 | 0.302 | 0.394 | 0.249 | 0.02 | 0.219 | 0.222 | 0.245 | 0.287 | 0.297 | 0.148 |
| R DorsAttnA ParOcc 1 | 0.044 | 0.018 | 0.024 | 0.026 | 0.038 | 0.08 | 0.092 | 0.069 | 0.258 | 0.025 | 0.22 | 0.224 | 0.256 | 0.305 | 0.317 | 0.159 |
| R DorsAttnA SPL 1 | 0.042 | 0.016 | 0.024 | 0.025 | 0.038 | 0.073 | 0.082 | 0.011 | 0.362 | 0.029 | 0.303 | 0.313 | 0.361 | 0.41 | 0.418 | 0.197 |
| R DorsAttnA SPL 2 | 0.147 | 0.044 | 0.066 | 0.075 | 0.145 | 0.22 | 0.235 | 0 | 0.442 | 0.029 | 0.384 | 0.394 | 0.441 | 0.489 | 0.498 | 0.188 |
| R DorsAttnA SPL 3 | 0.041 | 0.015 | 0.025 | 0.026 | 0.037 | 0.069 | 0.082 | 0.07 | 0.321 | 0.028 | 0.266 | 0.274 | 0.321 | 0.369 | 0.378 | 0.218 |
| R DorsAttnA SPL 4 | 0.053 | 0.024 | 0.026 | 0.027 | 0.045 | 0.102 | 0.116 | 0.022 | 0.435 | 0.031 | 0.375 | 0.385 | 0.434 | 0.486 | 0.494 | 0.244 |
| R DorsAttnB PostC 1 | 0.044 | 0.018 | 0.024 | 0.026 | 0.039 | 0.082 | 0.092 | 0 | 0.238 | 0.014 | 0.216 | 0.219 | 0.236 | 0.264 | 0.271 | 0 |
| R DorsAttnB PostC 2 | 0.043 | 0.017 | 0.024 | 0.025 | 0.038 | 0.075 | 0.086 | 0.028 | 0.24 | 0.015 | 0.217 | 0.22 | 0.238 | 0.269 | 0.275 | 0 |
| R DorsAttnB PostC 3 | 0.04 | 0.014 | 0.024 | 0.025 | 0.036 | 0.067 | 0.075 | 0 | 0.251 | 0.022 | 0.219 | 0.223 | 0.248 | 0.293 | 0.302 | 0.071 |
| R DorsAttnB PostC 4 | 0.042 | 0.016 | 0.024 | 0.025 | 0.037 | 0.074 | 0.088 | 0 | 0.24 | 0.015 | 0.218 | 0.22 | 0.238 | 0.269 | 0.278 | 0 |
| R DorsAttnB FEF 1 | 0.054 | 0.026 | 0.025 | 0.027 | 0.047 | 0.107 | 0.12 | 0.128 | 0.24 | 0.015 | 0.216 | 0.22 | 0.238 | 0.269 | 0.275 | 0.118 |
| R SalVentAttnA ParOper 1 | 0.051 | 0.023 | 0.025 | 0.027 | 0.045 | 0.099 | 0.115 | 0 | 0.239 | 0.014 | 0.217 | 0.22 | 0.237 | 0.267 | 0.273 | 0 |
| R SalVentAttnA PrC 1 | 0.038 | 0.012 | 0.024 | 0.025 | 0.035 | 0.061 | 0.067 | 0 | 0.305 | 0.029 | 0.251 | 0.258 | 0.304 | 0.355 | 0.366 | 0.051 |
| R SalVentAttnA Ins 1 | 0.078 | 0.035 | 0.028 | 0.03 | 0.074 | 0.141 | 0.156 | 0 | 0.245 | 0.018 | 0.218 | 0.221 | 0.242 | 0.279 | 0.288 | 0 |
| R SalVentAttnA Ins 2 | 0.039 | 0.013 | 0.024 | 0.025 | 0.036 | 0.065 | 0.071 | 0 | 0.237 | 0.013 | 0.216 | 0.219 | 0.235 | 0.261 | 0.268 | 0 |
| R SalVentAttnA FrOper 1 | 0.042 | 0.016 | 0.024 | 0.025 | 0.037 | 0.074 | 0.082 | 0 | 0.236 | 0.012 | 0.216 | 0.219 | 0.234 | 0.259 | 0.265 | 0 |
| R SalVentAttnA FrMed 1 | 0.038 | 0.013 | 0.024 | 0.025 | 0.035 | 0.065 | 0.075 | 0 | 0.245 | 0.017 | 0.219 | 0.222 | 0.242 | 0.277 | 0.286 | 0.152 |
| R SalVentAttnA ParMed 1 | 0.044 | 0.018 | 0.024 | 0.026 | 0.039 | 0.08 | 0.088 | 0.067 | 0.237 | 0.013 | 0.217 | 0.219 | 0.235 | 0.263 | 0.27 | 0.031 |
| R SalVentAttnA ParMed 2 | 0.039 | 0.013 | 0.024 | 0.025 | 0.036 | 0.063 | 0.073 | 0 | 0.236 | 0.012 | 0.216 | 0.219 | 0.234 | 0.259 | 0.263 | 0.01 |
| R SalVentAttnA FrMed 2 | 0.044 | 0.018 | 0.025 | 0.026 | 0.038 | 0.08 | 0.091 | 0.02 | 0.236 | 0.012 | 0.216 | 0.219 | 0.234 | 0.259 | 0.264 | 0 |
| R SalVentAttnB IPL 1 | 0.04 | 0.014 | 0.024 | 0.025 | 0.036 | 0.069 | 0.077 | 0 | 0.244 | 0.017 | 0.218 | 0.221 | 0.241 | 0.277 | 0.285 | 0 |
| R SalVentAttnB PFClv 1 | 0.087 | 0.038 | 0.028 | 0.032 | 0.082 | 0.155 | 0.17 | 0.108 | 0.247 | 0.018 | 0.22 | 0.223 | 0.243 | 0.282 | 0.29 | 0 |
| R SalVentAttnB PFCl 1 | 0.045 | 0.019 | 0.025 | 0.026 | 0.039 | 0.083 | 0.095 | 0.076 | 0.245 | 0.018 | 0.218 | 0.221 | 0.242 | 0.277 | 0.283 | 0.15 |
| R SalVentAttnB Ins 1 | 0.083 | 0.038 | 0.028 | 0.03 | 0.079 | 0.152 | 0.167 | 0 | 0.241 | 0.016 | 0.217 | 0.22 | 0.239 | 0.271 | 0.279 | 0.033 |
| R SalVentAttnB Ins 2 | 0.065 | 0.032 | 0.026 | 0.028 | 0.058 | 0.127 | 0.144 | 0 | 0.273 | 0.027 | 0.23 | 0.234 | 0.271 | 0.32 | 0.329 | 0.171 |
| R SalVentAttnB PFCmp 1 | 0.049 | 0.021 | 0.025 | 0.026 | 0.042 | 0.09 | 0.103 | 0 | 0.242 | 0.015 | 0.218 | 0.221 | 0.24 | 0.271 | 0.279 | 0.11 |
| R LimbicB OFC 2 | 0.05 | 0.022 | 0.025 | 0.026 | 0.044 | 0.094 | 0.111 | 0 | 0.247 | 0.019 | 0.218 | 0.222 | 0.244 | 0.284 | 0.292 | 0.049 |
| R LimbicB OFC 3 | 0.261 | 0.042 | 0.179 | 0.19 | 0.26 | 0.329 | 0.341 | 0.2 | 0.242 | 0.016 | 0.217 | 0.221 | 0.24 | 0.272 | 0.28 | 0 |
| R LimbicB OFC 4 | 0.057 | 0.027 | 0.025 | 0.027 | 0.051 | 0.11 | 0.126 | 0.007 | 0.241 | 0.015 | 0.218 | 0.22 | 0.238 | 0.269 | 0.276 | 0 |
| R LimbicA TempPole 1 | 0.125 | 0.044 | 0.046 | 0.055 | 0.124 | 0.199 | 0.216 | 0 | 0.267 | 0.025 | 0.226 | 0.23 | 0.265 | 0.311 | 0.321 | 0 |
| R LimbicA TempPole 2 | 0.041 | 0.015 | 0.024 | 0.025 | 0.037 | 0.07 | 0.079 | 0 | 0.259 | 0.025 | 0.222 | 0.226 | 0.255 | 0.304 | 0.315 | 0 |
| R LimbicA TempPole 3 | 0.439 | 0.037 | 0.36 | 0.374 | 0.441 | 0.498 | 0.507 | 0 | 0.449 | 0.032 | 0.388 | 0.396 | 0.45 | 0.5 | 0.506 | 0.112 |
| R LimbicA TempPole 4 | 0.213 | 0.046 | 0.125 | 0.139 | 0.211 | 0.287 | 0.301 | 0.211 | 0.288 | 0.031 | 0.229 | 0.237 | 0.288 | 0.342 | 0.35 | 0.096 |
| R ContA IPS 1 | 0.041 | 0.016 | 0.024 | 0.025 | 0.036 | 0.074 | 0.084 | 0 | 0.37 | 0.031 | 0.308 | 0.318 | 0.371 | 0.42 | 0.429 | 0.251 |
| R ContA IPS 2 | 0.038 | 0.012 | 0.024 | 0.025 | 0.035 | 0.061 | 0.068 | 0 | 0.239 | 0.014 | 0.217 | 0.22 | 0.236 | 0.265 | 0.273 | 0 |
| R ContA PFCd 1 | 0.053 | 0.025 | 0.025 | 0.027 | 0.046 | 0.105 | 0.121 | 0.098 | 0.241 | 0.015 | 0.217 | 0.221 | 0.238 | 0.269 | 0.275 | 0.075 |
| R ContA PFCl 1 | 0.038 | 0.013 | 0.024 | 0.025 | 0.035 | 0.062 | 0.069 | 0 | 0.251 | 0.02 | 0.22 | 0.223 | 0.248 | 0.288 | 0.298 | 0.175 |
| R ContA PFCl 2 | 0.044 | 0.018 | 0.024 | 0.025 | 0.039 | 0.081 | 0.09 | 0 | 0.254 | 0.022 | 0.222 | 0.225 | 0.251 | 0.294 | 0.304 | 0.288 |
| R ContA Cingm 1 | 0.054 | 0.025 | 0.025 | 0.027 | 0.047 | 0.105 | 0.119 | 0 | 0.307 | 0.03 | 0.25 | 0.259 | 0.307 | 0.356 | 0.367 | 0.115 |
| R ContB Temp 1 | 0.097 | 0.04 | 0.032 | 0.037 | 0.094 | 0.169 | 0.187 | 0.062 | 0.247 | 0.018 | 0.22 | 0.223 | 0.244 | 0.282 | 0.289 | 0.226 |
| R ContB Temp 2 | 0.134 | 0.043 | 0.053 | 0.064 | 0.134 | 0.208 | 0.225 | 0.034 | 0.244 | 0.017 | 0.219 | 0.222 | 0.241 | 0.276 | 0.284 | 0.007 |
| R ContB IPL 1 | 0.074 | 0.035 | 0.026 | 0.029 | 0.068 | 0.139 | 0.155 | 0 | 0.308 | 0.029 | 0.254 | 0.262 | 0.306 | 0.357 | 0.366 | 0 |
| R ContB IPL 2 | 0.039 | 0.014 | 0.024 | 0.025 | 0.035 | 0.066 | 0.076 | 0 | 0.293 | 0.028 | 0.241 | 0.248 | 0.293 | 0.341 | 0.351 | 0.093 |
| R ContB PFCld 1 | 0.045 | 0.018 | 0.025 | 0.026 | 0.04 | 0.081 | 0.091 | 0 | 0.255 | 0.022 | 0.221 | 0.224 | 0.252 | 0.296 | 0.307 | 0.11 |
| R ContB PFCld 2 | 0.038 | 0.012 | 0.024 | 0.025 | 0.035 | 0.061 | 0.069 | 0 | 0.237 | 0.013 | 0.216 | 0.219 | 0.235 | 0.261 | 0.265 | 0.03 |
| R ContB PFClv 1 | 0.045 | 0.018 | 0.025 | 0.026 | 0.039 | 0.083 | 0.093 | 0 | 0.238 | 0.013 | 0.216 | 0.219 | 0.236 | 0.264 | 0.268 | 0 |
| R ContB PFClv 2 | 0.127 | 0.045 | 0.046 | 0.057 | 0.123 | 0.206 | 0.221 | 0.025 | 0.244 | 0.018 | 0.218 | 0.221 | 0.241 | 0.276 | 0.287 | 0 |
| R ContB PFCmp 1 | 0.037 | 0.011 | 0.024 | 0.025 | 0.034 | 0.058 | 0.064 | 0 | 0.236 | 0.013 | 0.216 | 0.219 | 0.235 | 0.26 | 0.267 | 0.19 |
| R ContB PFCld 3 | 0.039 | 0.013 | 0.024 | 0.025 | 0.036 | 0.067 | 0.076 | 0 | 0.239 | 0.014 | 0.216 | 0.219 | 0.237 | 0.264 | 0.27 | 0.139 |
| R ContC pCun 1 | 0.044 | 0.018 | 0.025 | 0.026 | 0.039 | 0.08 | 0.09 | 0 | 0.256 | 0.023 | 0.222 | 0.225 | 0.253 | 0.3 | 0.31 | 0.021 |
| R ContC pCun 2 | 0.115 | 0.043 | 0.036 | 0.046 | 0.113 | 0.191 | 0.206 | 0.032 | 0.252 | 0.021 | 0.219 | 0.223 | 0.248 | 0.289 | 0.299 | 0.157 |
| R ContC Cingp 1 | 0.05 | 0.022 | 0.025 | 0.026 | 0.043 | 0.092 | 0.107 | 0 | 0.318 | 0.035 | 0.234 | 0.255 | 0.319 | 0.373 | 0.387 | 0.395 |
| R DefaultA IPL 1 | 0.045 | 0.018 | 0.025 | 0.026 | 0.039 | 0.083 | 0.093 | 0 | 0.246 | 0.018 | 0.219 | 0.223 | 0.244 | 0.279 | 0.288 | 0.19 |
| R DefaultA PFCd 1 | 0.039 | 0.013 | 0.024 | 0.025 | 0.035 | 0.063 | 0.071 | 0 | 0.236 | 0.012 | 0.215 | 0.218 | 0.234 | 0.258 | 0.263 | 0.193 |
| R DefaultA pCunPCC 1 | 0.124 | 0.043 | 0.047 | 0.055 | 0.123 | 0.199 | 0.215 | 0.089 | 0.261 | 0.025 | 0.223 | 0.227 | 0.258 | 0.307 | 0.317 | 0.109 |
| R DefaultA PFCm 1 | 0.136 | 0.043 | 0.048 | 0.066 | 0.135 | 0.21 | 0.224 | 0.125 | 0.238 | 0.014 | 0.217 | 0.219 | 0.235 | 0.264 | 0.269 | 0 |
| R DefaultA PFCm 2 | 0.222 | 0.045 | 0.135 | 0.15 | 0.222 | 0.296 | 0.313 | 0 | 0.248 | 0.02 | 0.218 | 0.222 | 0.245 | 0.285 | 0.294 | 0 |
| R DefaultA PFCm 3 | 0.045 | 0.018 | 0.024 | 0.026 | 0.04 | 0.081 | 0.095 | 0 | 0.238 | 0.014 | 0.216 | 0.219 | 0.236 | 0.265 | 0.273 | 0.094 |
| R DefaultB Temp 1 | 0.065 | 0.032 | 0.026 | 0.028 | 0.057 | 0.13 | 0.141 | 0.009 | 0.237 | 0.013 | 0.216 | 0.219 | 0.236 | 0.263 | 0.268 | 0 |
| R DefaultB AntTemp 1 | 0.043 | 0.017 | 0.025 | 0.026 | 0.038 | 0.077 | 0.088 | 0 | 0.239 | 0.014 | 0.218 | 0.22 | 0.237 | 0.265 | 0.272 | 0 |
| R DefaultB PFCd 1 | 0.057 | 0.027 | 0.025 | 0.027 | 0.05 | 0.109 | 0.122 | 0.067 | 0.242 | 0.016 | 0.217 | 0.22 | 0.24 | 0.272 | 0.28 | 0.013 |
| R DefaultB PFCv 1 | 0.051 | 0.024 | 0.025 | 0.026 | 0.045 | 0.102 | 0.117 | 0 | 0.237 | 0.013 | 0.216 | 0.219 | 0.235 | 0.262 | 0.268 | 0 |
| R DefaultC IPL 1 | 0.092 | 0.039 | 0.029 | 0.034 | 0.088 | 0.161 | 0.177 | 0.142 | 0.248 | 0.019 | 0.22 | 0.223 | 0.245 | 0.283 | 0.293 | 0.057 |
| R DefaultC Rsp 1 | 0.152 | 0.045 | 0.066 | 0.08 | 0.151 | 0.231 | 0.248 | 0 | 0.25 | 0.021 | 0.22 | 0.223 | 0.247 | 0.288 | 0.296 | 0 |
| R DefaultC PHC 1 | 0.043 | 0.017 | 0.025 | 0.026 | 0.039 | 0.078 | 0.088 | 0 | 0.249 | 0.02 | 0.22 | 0.223 | 0.245 | 0.287 | 0.295 | 0.123 |
| R TempPar 1 | 0.041 | 0.016 | 0.024 | 0.025 | 0.037 | 0.072 | 0.081 | 0.053 | 0.235 | 0.012 | 0.216 | 0.218 | 0.234 | 0.258 | 0.263 | 0 |
| R TempPar 2 | 0.039 | 0.013 | 0.024 | 0.025 | 0.035 | 0.064 | 0.072 | 0.032 | 0.236 | 0.012 | 0.215 | 0.218 | 0.234 | 0.258 | 0.263 | 0.023 |
| R TempPar 3 | 0.055 | 0.027 | 0.025 | 0.027 | 0.047 | 0.11 | 0.126 | 0.052 | 0.239 | 0.014 | 0.217 | 0.22 | 0.237 | 0.267 | 0.274 | 0.133 |
| R TempPar 4 | 0.037 | 0.011 | 0.024 | 0.025 | 0.034 | 0.06 | 0.067 | 0.107 | 0.237 | 0.013 | 0.216 | 0.219 | 0.235 | 0.26 | 0.267 | 0.044 |
| L Thalamus | 0.038 | 0.012 | 0.024 | 0.025 | 0.035 | 0.061 | 0.068 | 0.002 | 0.237 | 0.013 | 0.216 | 0.219 | 0.235 | 0.26 | 0.267 | 0.019 |
| L Caudate | 0.169 | 0.043 | 0.091 | 0.101 | 0.167 | 0.24 | 0.253 | 0.204 | 0.241 | 0.016 | 0.217 | 0.221 | 0.239 | 0.271 | 0.278 | 0.023 |
| L Putamen | 0.04 | 0.014 | 0.024 | 0.025 | 0.036 | 0.067 | 0.077 | 0 | 0.236 | 0.012 | 0.215 | 0.219 | 0.234 | 0.258 | 0.264 | 0.106 |
| L Pallidum | 0.096 | 0.041 | 0.03 | 0.035 | 0.093 | 0.168 | 0.183 | 0.17 | 0.24 | 0.015 | 0.217 | 0.22 | 0.238 | 0.269 | 0.276 | 0.131 |
| L Hippocampus | 0.064 | 0.032 | 0.026 | 0.028 | 0.057 | 0.124 | 0.139 | 0 | 0.239 | 0.014 | 0.216 | 0.22 | 0.236 | 0.265 | 0.271 | 0 |
| L Amygdala | 0.079 | 0.037 | 0.028 | 0.031 | 0.073 | 0.145 | 0.165 | 0 | 0.24 | 0.014 | 0.216 | 0.22 | 0.238 | 0.266 | 0.272 | 0 |
| L Accumbens | 0.24 | 0.044 | 0.155 | 0.17 | 0.24 | 0.31 | 0.325 | 0.002 | 0.318 | 0.029 | 0.265 | 0.272 | 0.317 | 0.367 | 0.377 | 0 |
| R Thalamus | 0.036 | 0.01 | 0.024 | 0.025 | 0.033 | 0.055 | 0.062 | 0 | 0.237 | 0.013 | 0.215 | 0.219 | 0.235 | 0.261 | 0.266 | 0.11 |
| R Caudate | 0.112 | 0.043 | 0.038 | 0.046 | 0.11 | 0.184 | 0.203 | 0.133 | 0.244 | 0.018 | 0.217 | 0.221 | 0.241 | 0.278 | 0.285 | 0.108 |
| R Putamen | 0.04 | 0.014 | 0.024 | 0.025 | 0.036 | 0.068 | 0.079 | 0 | 0.237 | 0.013 | 0.216 | 0.219 | 0.235 | 0.261 | 0.266 | 0.147 |
| R Pallidum | 0.072 | 0.034 | 0.027 | 0.029 | 0.066 | 0.136 | 0.147 | 0.02 | 0.237 | 0.014 | 0.215 | 0.218 | 0.235 | 0.263 | 0.269 | 0 |
| R Hippocampus | 0.051 | 0.024 | 0.025 | 0.026 | 0.044 | 0.1 | 0.115 | 0 | 0.237 | 0.013 | 0.215 | 0.219 | 0.235 | 0.261 | 0.267 | 0 |
| R Amygdala | 0.064 | 0.032 | 0.026 | 0.028 | 0.057 | 0.13 | 0.14 | 0.142 | 0.236 | 0.012 | 0.215 | 0.218 | 0.234 | 0.258 | 0.265 | 0 |
| R Accumbens | 0.274 | 0.045 | 0.183 | 0.2 | 0.275 | 0.347 | 0.361 | 0.002 | 0.272 | 0.026 | 0.228 | 0.233 | 0.269 | 0.319 | 0.329 | 0 |
